# Carbon-phosphorus cycle models overestimate CO_2_ enrichment response in a mature *Eucalyptus* forest

**DOI:** 10.1126/sciadv.adl5822

**Published:** 2024-07-03

**Authors:** Mingkai Jiang, Belinda E. Medlyn, David Wårlind, Jürgen Knauer, Katrin Fleischer, Daniel S. Goll, Stefan Olin, Xiaojuan Yang, Lin Yu, Sönke Zaehle, Haicheng Zhang, He Lv, Kristine Y. Crous, Yolima Carrillo, Catriona Macdonald, Ian Anderson, Matthias M. Boer, Mark Farrell, Andrew Gherlenda, Laura Castañeda-Gómez, Shun Hasegawa, Klaus Jarosch, Paul Milham, Raúl Ochoa-Hueso, Varsha Pathare, Johanna Pihlblad, Juan Piñeiro Nevado, Jeff Powell, Sally A. Power, Peter Reich, Markus Riegler, David S. Ellsworth, Benjamin Smith

**Affiliations:** ^1^ College of Life Sciences, Zhejiang University, Hangzhou, Zhejiang 310000, China.; ^2^ Hawkesbury Institute for the Environment, Western Sydney University, Locked Bag 1797, Penrith 2751, Australia.; ^3^ Department of Physical Geography and Ecosystem Science, Faculty of Science, Lund University, Lund, Sweden.; ^4^ CSIRO Environment Canberra, Canberra, ACT, Australia.; ^5^ Max Planck Institute for Biogeochemistry, Hans-Knöll-Str. 10, 07745 Jena, Germany.; ^6^ Laboratoire des Sciences du Climat et de l’Environnement, CEA-CNRS-UVSQ-Université Paris-Saclay, Gif-sur-Yvette, France.; ^7^ Environmental Sciences Division, Oak Ridge National Laboratory, Oak Ridge, TN 37831, USA.; ^8^ Department of Earth System Sciences, Hamburg University, Allende-Platz 2, 20146 Hamburg, Germany.; ^9^ Carbon-Water Research Station in Karst Regions of Northern Guangdong, School of Geography and Planning, Sun Yat-Sen University, Guangzhou 510006, China.; ^10^ CSIRO Agriculture and Food, Kaurna Country, Locked Bag 2, Glen Osmond, SA 5064, Australia.; ^11^ SouthPole Environmental Services, Zurich, Switzerland.; ^12^ Department of Forestry and Climate, Norwegian Institute of Bioeconomy Research (NIBIO), Ås, Norway.; ^13^ Agroecology and Environment, Agroscope, Zürich-Reckenholz, Switzerland.; ^14^ Soil Science, Institute of Geography, University of Bern, Bern, Switzerland.; ^15^ Oeschger Centre for Climate Change Research, University of Bern, 3012 Bern, Switzerland.; ^16^ Department of Biology, IVAGRO, University of Cádiz, Campus de Excelencia Internacional Agroalimentario (CeiA3), Campus del Rio San Pedro, 11510 Puerto Real, Cádiz, Spain.; ^17^ Department of Terrestrial Ecology, Netherlands Institute of Ecology (NIOO-KNAW), P.O. Box 50, 6700 AB, Wageningen, Netherlands.; ^18^ Institute of Genomic Biology, University of Illinois at Urbana-Champaign, Champaign, IL 61801, USA.; ^19^ Birmingham Institute for Forest Research, University of Birmingham, Edgbaston B15 2TT, UK.; ^20^ School of Geography, University of Birmingham, Edgbaston B15 2TT, UK.; ^21^ ETSI Montes, Forestal y del Medio Natural, Universidad Politécnica de Madrid, Ciudad Universitaria s/n, 28040 Madrid, Spain.; ^22^ Department of Forest Resources, University of Minnesota, St. Paul, MN 55108, USA.; ^23^ Institute for Global Change Biology, and School for the Environment and Sustainability, University of Michigan, Ann Arbor, MI 48109, USA.

## Abstract

The importance of phosphorus (P) in regulating ecosystem responses to climate change has fostered P-cycle implementation in land surface models, but their CO_2_ effects predictions have not been evaluated against measurements. Here, we perform a data-driven model evaluation where simulations of eight widely used P-enabled models were confronted with observations from a long-term free-air CO_2_ enrichment experiment in a mature, P-limited *Eucalyptus* forest. We show that most models predicted the correct sign and magnitude of the CO_2_ effect on ecosystem carbon (C) sequestration, but they generally overestimated the effects on plant C uptake and growth. We identify leaf-to-canopy scaling of photosynthesis, plant tissue stoichiometry, plant belowground C allocation, and the subsequent consequences for plant-microbial interaction as key areas in which models of ecosystem C-P interaction can be improved. Together, this data-model intercomparison reveals data-driven insights into the performance and functionality of P-enabled models and adds to the existing evidence that the global CO_2_-driven carbon sink is overestimated by models.

## Teaser

A data-model intercomparison identifies key model deficiencies to make mechanistic CO_2_ predictions in a phosphorus-limited forest.

## INTRODUCTION

Land surface models and their predictions have a key role in providing the evidence to guide climate and emissions policy (*1*, *2*). The capacity of these models to realistically resolve biogeochemical processes and make accurate predictions of ecosystem responses to changing environmental conditions thus underpins our actions to mitigate climate change (*3*, *4*). Phosphorus (P), an element essential for plant growth and metabolism (*5*), is increasingly recognized as having globally substantial effects on biogeochemistry via its role in regulating terrestrial ecosystem productivity (*6*, *7*) and its response to rising atmospheric CO_2_ concentrations (*3*, *4*). As a result, P-cycle processes are now being implemented in land surface models (*8*–*13*), and these models generally predicted reduced global land C sink relative to those without P-cycle representations (*14*, *15*). This stands in broad agreement with findings of P fertilization experiments that demonstrate that P limitation is globally widespread (*6*). However, whether P-enabled models can make accurate predictions on correct mechanistic grounds of the effects of rising atmospheric CO_2_ on plant growth and ecosystem C sequestration remains untested. This critical knowledge gap leads to uncertainty in estimating the land C sink and the C-climate feedback under climate change (*16*).

Multimodel intercomparisons show that P-enabled models diverge in their predictions of the CO_2_ fertilization effect, reflecting, in part, different model assumptions on plant P-use and acquisition strategies (*17*, *18*). In one such intercomparison performed for a P-limited *Eucalyptus* forest (*17*), the two P-enabled models predicted lower CO_2_ effects on growth than models lacking P-cycle processes, but no observations were available to evaluate the model predictions. Nevertheless, this study highlighted the need to develop an increased understanding of the processes related to plant P uptake, plant stoichiometry, and their interactions with soil microbial communities (*17*) and sparked the increased interests in developing P-enabled models (*10*–*13*, *19*, *20*). A more recent model intercomparison using a larger suite of P-enabled models tested predictions of the CO_2_ responses of a tropical rainforest growing on low-P soils (*18*). The limiting role of P was again demonstrated, but predictions of the CO_2_ effect still varied widely among models with no data to constrain the prediction uncertainties. Specifically, while some models predicted no additional growth under elevated CO_2_ (eCO_2_), others predicted larger responses, facilitated by (i) plasticity in plant stoichiometry and allocation, (ii) additional fine root production, (iii) greater P mobilization via P desorption, and/or (iv) extra biochemical mineralization of soil organic P (*18*). Given the large spread of predictions among P-enabled models, an evaluation against data is now urgently needed to constrain alternative model assumptions.

The *Eucalyptus* forest free-air CO_2_ enrichment experiment (EucFACE) provides a unique opportunity to evaluate model simulations with data. EucFACE is an ecosystem-scale field experiment where three plots (490 m^2^ each) are subjected to ambient and eCO_2_ treatments (+150 μmol mol^−1^) in a natural, mature *Eucalyptus* forest on soils of low fertility. This work uses data collected over the first 7 years of the experiment (2012 to 2018). A P fertilization experiment in the adjacent forest stand has demonstrated that soil P availability limits tree productivity at the site (*21*, *22*). In the EucFACE experiment, multiple independent data streams show that net ecosystem production (NEP) did not increase under eCO_2_ (*23*). More specifically, eCO_2_ led to an enhanced photosynthetic uptake by trees (*21*, *24*), but they did not grow extra biomass under eCO_2_ over the first 4 years of the CO_2_ enrichment (*23*). Instead, it appears likely that the extra C was deployed by the trees to facilitate P acquisition through possible increased belowground labile C allocation (*23*). This mechanism (known as priming) has been widely suggested to assist soil microbial and mycorrhizal communities to release nutrients that would otherwise be unavailable to plants (*25*). At EucFACE, it was associated with enhanced heterotrophic respiration (R_het_) under eCO_2_ (*23*, *26*). These results suggest that capturing the full spectrum of plant-soil interactions involving C-P feedback is important for models to predict the extra potential for C sequestration with CO_2_ fertilization and provide a unique opportunity to evaluate the recent developments in P-enabled models.

**Fig. 1.**
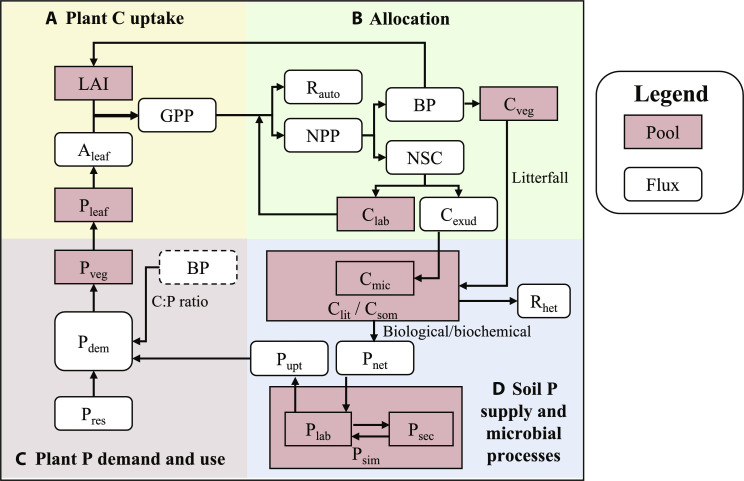
Schematic diagrams of the key ecosystem components and processes evaluated in this data-model intercomparison. (**A**) Plant carbon (C) uptake processes, including leaf physiological (carbon assimilation rate per leaf area; A_leaf_) and structural controls (LAI) on GPP. The physiological control can be further related to how leaf phosphorus content (P_leaf_) affects A_leaf_. (**B**) Plant C allocation processes. GPP is partitioned into autotrophic respiration (R_auto_) and NPP, with the latter further partitioned into BP and nonstructural carbohydrate (NSC) fluxes. Plant C allocation is resource-dependent, controlled by model assumptions on plant tissue stoichiometry and the corresponding growth demand. BP leads to C storage in vegetation (C_veg_), and NSC flux leads to either C accumulation in plant labile C pool (C_lab_) or plant root exudation flux (C_exud_). C_exud_ can be considered as plant C cost for nutrient acquisition. (**C**) Plant P demand and use processes. Plant P demand (P_dem_) is driven by BP and modulated by plant C:P ratios to build the vegetation P pool (P_veg_). P_dem_ can be met by plant P resorption (P_res_) and uptake (P_upt_) fluxes. The dashed box is a surrogate of the BP flux as seen in the allocation subpanel. (**D**) Soil P supply and microbial processes. Plant-sourced C enters soil via litterfall and C_exud_ fluxes, and the microbial turnover of these organic matter releases C via R_het_. Organic P is mineralized (P_net_) via both biological and biochemical processes, which then enters soil labile P pool (P_lab_) to meet plant P uptake (P_upt_). P_sim_ and P_sec_ represent P pools of soil inorganic matter and secondary inorganic matter, respectively.

**Fig. 2.**
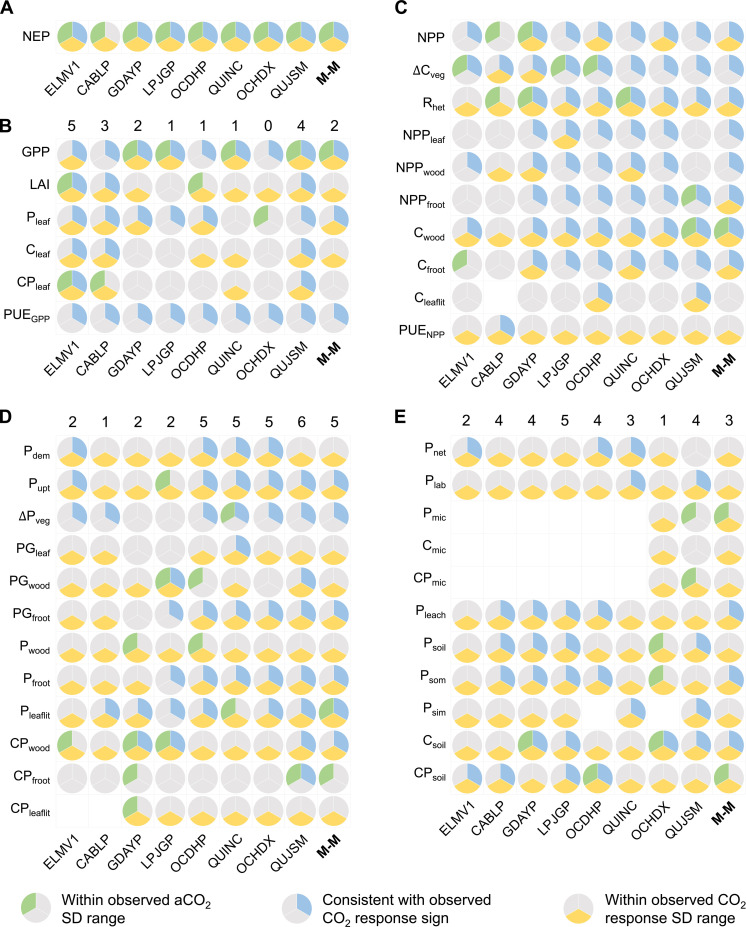
Data-model intercomparisons for the CO_2_ responses of key groups of ecosystem carbon (C) and phosphorus (P) variables. (**A**) NEP, indicating the overall ecosystem carbon sequestration potential; (**B** to **E**) plant C uptake, plant C allocation, plant P demand and use, and soil P process variables, respectively. The three colors in the pie chart show different assessment categories: green indicates that the model prediction is within the range of observational uncertainty (expressed as the SD of the data) under ambient CO_2_ (aCO_2_) treatment; blue and yellow indicate that the model prediction is consistent with observation in terms of the sign and the magnitude of the CO_2_ effect, respectively. The former indicator provides a baseline understanding of the model performance under aCO_2_ treatment, and the latter two indicators assess the correctness of the model prediction in terms of the direction and accuracy of the CO_2_ response, respectively. The gray color in the pie chart indicates data-model inconsistency, and the white space indicates no model output for the particular variable. M-M represents multimodel means. Variable abbreviations are: GPP, NPP, annual incremental change in plant C pool (∆C_veg_), LAI, and R_het_; NPP, C pools, P pools, and C:P ratios of different plant and ecosystem compartments, with froot, leaflit, lab, mic, som, and sim indicating fine root, leaflitter, soil labile P, soil microbes, soil organic matter, and soil inorganic matter, respectively; plant P-use efficiency to support GPP and NPP (PUE_GPP_ and PUE_NPP_, respectively); plant P demand and uptake fluxes (P_dem_ and P_upt_, respectively); incremental changes in plant P pool (∆P_veg_); plant P demand fluxes driven by leaf, wood, and fine root production (PG_leaf_, PG_wood_, and PG_froot_, respectively); and soil net P mineralization and P leaching fluxes (P_net_ and P_leach_, respectively).

**Fig. 3.**
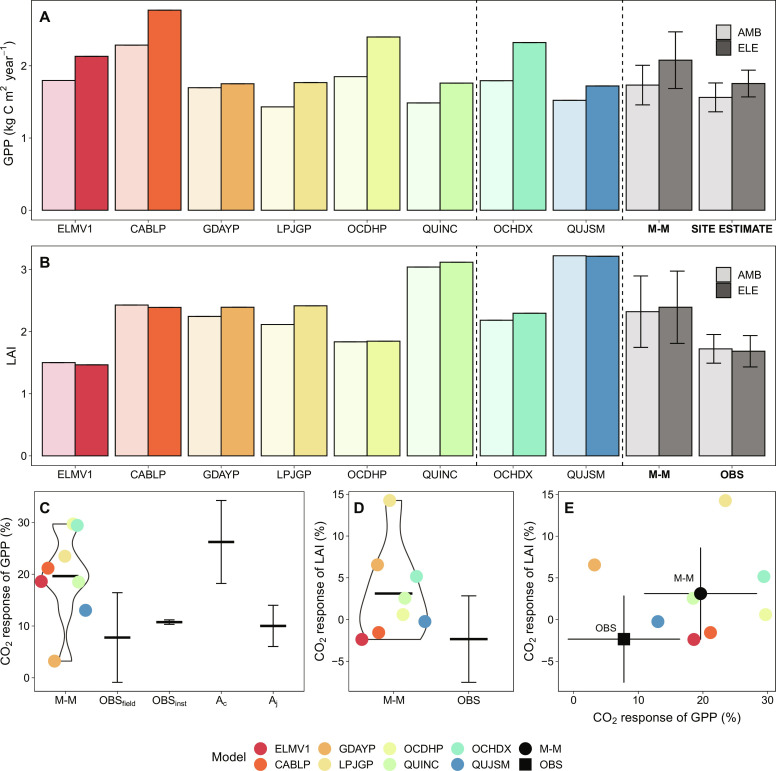
Data-model intercomparisons for the CO_2_ responses of plant carbon uptake variables. M-M, SITE ESTIMATE, OBS, GPP, and LAI represent multimodel means, site estimate of GPP was based on a detailed stand-scale tree physiology model (MAESPA) because no direct measurement of canopy photosynthesis is available (*24*), observations, GPP, and LAI, respectively. OBS_field_ represents field-based estimate of the CO_2_ effect on GPP, with treatment-level variation in LAI included, whereas OBS_inst_ represents the normalized estimate of the CO_2_ effect on GPP (i.e., treatment-level variation in LAI normalized to site-averaged LAI). A_c_ and A_j_ represent the response of Rubisco-limited and RuBP-regeneration-limited leaf photosynthesis, respectively, as estimated by the detailed stand-scale canopy model MAESPA in (*24*). (**A**) Annual GPP under ambient and eCO_2_ treatment. (**B**) LAI under ambient and eCO_2_ treatment (unitless). (**C**) CO_2_ effect of GPP. (**D**) CO_2_ effect of LAI. (**E**) Emergent constraint on the CO_2_ response of GPP and the CO_2_ response of LAI. Error bars indicate the SD of the multimodel means (*n* = 8) and observation-based treatment means (*n* = 3).

**Fig. 4.**
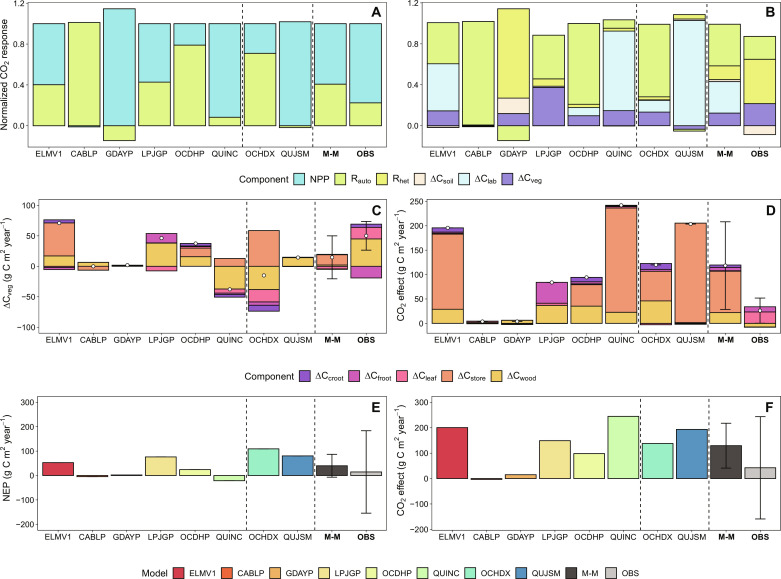
Data-model intercomparisons on plant carbon (C) allocation, BP, and NEP responses to eCO_2_. (**A**) Fate of the additional C under eCO_2_ in the ecosystem (unitless), with all fluxes normalized to model-specific CO_2_ effect on GPP. The GPP response is partitioned into NPP and autotrophic respiration (R_auto_) responses. (**B**) The normalized GPP response (unitless) is partitioned into ecosystem respiration and net ecosystem C storage, with the former including R_auto_ and R_het_ and the latter including annual C increment in vegetation biomass (ΔC_veg_), plant storage carbon pools (ΔC_store_), and soil carbon pools (ΔC_soil_). (**C**) BP under ambient CO_2_ treatment (g C m^−2^ year^−1^), split into annual incremental changes in leaf, wood, fine root, coarse root, and plant labile C pools (ΔC_leaf_, ΔC_wood_, ΔC_froot_, ΔC_croot_, and ΔC_lab_, respectively). (**D**) CO_2_ effect on BP (g C m^−2^ year^−1^). (**E**) NEP under ambient CO_2_ treatment (g C m^−2^ year^−1^). (**F**) CO_2_ effect on NEP (g C m^−2^ year^−1^). Error bars indicate the SD of the multimodel means (*n* = 8) and observation-based treatment means (*n* = 3). The error bars for site-specific NEP estimates take into account the uncertainties associated with the three independent ways of estimating NEP (i.e., total ecosystem influx minus outflux, NPP minus soil R_het_, and the sum of annual incremental change in all ecosystem C pools), as reported in (*23*).

**Fig. 5.**
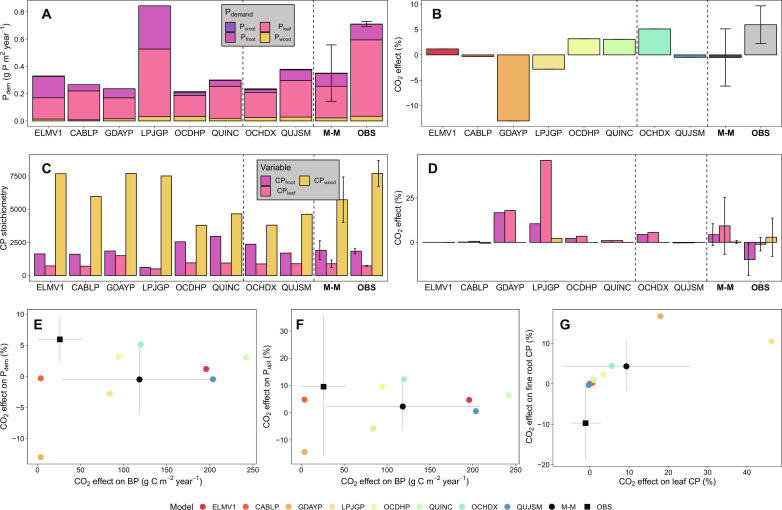
Data-model intercomparison of key plant phosphorus-cycle variables and their responses to eCO_2_. (**A**) Plant P demand flux (P_dem_) under ambient CO_2_ treatment, calculated as the sum of annual production fluxes of leaf, wood, fine root, and coarse root (PG_leaf_, PG_wood_, PG_froot_, and PG_croot_, respectively) in data and models. (**B**) CO_2_ effect on P_dem_. (**C**) Plant C:P ratios (CP stoichiometry) under ambient CO_2_ treatment, split into C:P ratios in leaf, wood, and fine root (CP_leaf_, CP_wood_, and CP_froot_, respectively). (**D**) CO_2_ effect on CP stoichiometry. (**E** and **F**) Emergent constraints of the CO_2_ effects on BP (g C m^−2^ year^−1^) and the CO_2_ effects (%) on P_dem_ and plant P uptake flux (P_upt_). (**G**) Emergent constraint of the CO_2_ effects on leaf C:P ratio (%) and fine root C:P ratio (%). Error bars indicate the SD of the multimodel means (*n* = 8) and observation-based treatment means (*n* = 3).

**Fig. 6.**
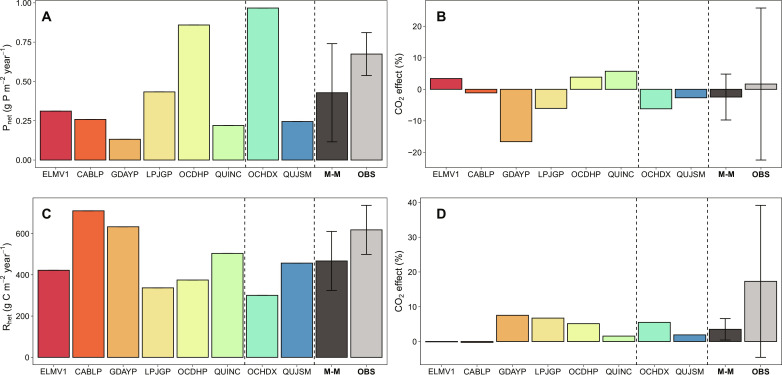
Data-model intercomparisons of key soil phosphorus-cycle variables under ambient CO_2_ treatment and their responses to eCO_2_. (**A**) Net soil P mineralization (P_net_) under ambient CO_2_ treatment. (**B**) CO_2_ effect on P_net_. (**C**) Soil labile soil R_het_ under ambient CO_2_ treatment. (**D**) CO_2_ effect on R_het_. M-M and OBS represent multimodel mean and observation, respectively. Error bars indicate the SD of the multimodel means (*n* = 8) and observation-based treatment means (*n* = 3).

Here, we confront the predictions of eight widely used models that explicitly simulate P-cycle processes using data collected from EucFACE. The set of models is diverse, ranging from stand-scale ecosystem models (*27*, *28*) to global land surface models with the capacity to simulate C-, nitrogen (N)-, and P-cycle processes (Table 1, Supplementary Information section 1, and tables S1 to S6) (*10*–*13*, *19*, *20*, *29*, *30*). Our analysis takes an assumption-based approach (*31*), meaning that we focus on the key underlying assumptions leading to the prediction rather than the prediction accuracy alone. We address the following four process-centered questions (Fig. 1 and fig. S1): (i) How do leaf physiology and leaf area jointly affect tree gross primary production (GPP) response to eCO_2_? (ii) how does eCO_2_ affect plant C allocation? (iii) how does eCO_2_ affect plant P demand (P_dem_) and use? and (iv) how does eCO_2_ affect plant P uptake (P_upt_) and soil P supply? We evaluate model predictions by assessing their prediction accuracy against measurements (Fig. 2) and the underlying mechanisms leading to the prediction (Figs. 3 to 6). Our work represents a crucial observation-based evaluation of ecosystem C-P interactions and their responses to eCO_2_ in an ensemble of P-enabled models, a necessary step to further constrain the uncertainty in the CO_2_ fertilization effect on forests globally.

**Table 1.** Overview of the mechanistic models included in this study and their key phosphorus-related model representation assumptions.

Model	CABLE-POP	ELM	GDAY-CNP	LPJ-GUESS- CNP	ORCHIDEE- CNP (v1.2)	QUINCY	ORCHIDEE- CNP (v1.3)	QUINCY-JSM
Abbreviation	CABLP	ELMV1	GDAYP	LPJGP	OCHDP	QUINC	OCHDX	QUJSM
Model type	Land surface model with a woody demography module	Land surface model	Stand-scale model	Global dynamic vegetation model	Land surface model	Land surface model	Land surface model with a MIMICS- type microbial submodule	Land surface model coupled to the Jena Soil Model
**P effect on key C-cycle processes**								
**Photosynthesis**	Down -regulation of *V*_cmax_ and *J*_max_ via leaf N:P (*8*)	Photosynthetic capacity func tion of leaf N content (*70*)	Down- regulation of *V*_cmax_ and *J*_max_ via leaf N and P (*67*)	Down- regulation of *V*_cmax_ and *J*_max_ via leaf N and P (*53*)	Down- regulation of *V*_cmax_ and *J*_max_ via leaf N and P (*53*)	Down- regulation of *V*_cmax_ and *J*_max_ via leaf N:P (*65*)	Same as OCHDP	Same as QUINC
**Growth**	Reduction of growth efficiency and direct down- regulation of NPP (excess C is lost via autotrophic respiration)	Direct down- regulation of NPP (excess C enters storage pool and lost via its turn over)	Direct down- regulation of NPP	Direct down- regulation of NPP	Direct down- regulation of growth using the min of plant labile C, N, and P (ex cess elements are stored)	Sink limitation of plant labile pool (*66*, *71*)	Same as OCHDP	Same as QUINC
**C allocation**	Fixed fractions to leaf, wood, and fine root	Dynamic allocation	Functional allometric relationship based on the pipe model and resource dependency					
**Soil decomposition**	Decompo sition con strained by soil labile P pool	Decom position con strained by soil solution P	None	Decom position constrained by inorganic soil P pool	Decom position constrained by dissolved labile P pool	None	Soil mineral P affects mi crobial C use efficiency	Soil mineral P affects microbial C use efficiency, microbial en zyme allocation, and competition for soluble P
**P-cycle processes**								
**P weathering**	Prescribed parameter (soil-type specific)	Function of soil primary mineral P pool and soil order	Prescribed	Depend on soil layer min eral to organic fraction, T, moisture, and root density	Set to zero for this site	Function of soil prima ry P pool, temperature, moisture, and root density	Same as OCHDP	Similar as QUINC, with additional con trol of microbial biomass
**P leaching**	Function of in organic labile P pool	Function of solution P pool, drainage, and runoff	Function of soil inorganic labile P pool	Mineral leaching is a function of PO_4_ pool, drainage, and runoff. Organic leaching also depends on soil sand fraction	Function of solution P pool, drainage, and runoff	Function of solution P pool, drainage, and runoff	Same as OCHDP	Similar as QUINC, with additional P leaching from DOM
**Soil P pools specific to P cycle**	Three pools (labile, sorbed, and strongly sorbed)	Five pools (solution, labile, second ary mineral, occluded, and primary mineral)	Five pools (parent, labile, sorbed, strong ly sorbed, and occluded)	Four pools (PO_4_, labile, sorbed, oc cluded)	Two pools (la bile dissolved and labile sorbed)	Five pools (soluble, adsorbed, absorbed, occluded, and primary)	Same as OCHDP	Same as QUINC
**Plant P retranslocation**	Constant coef ficients for leaf, wood, and fine root pools	Constant coefficient for leaf only	Constant coefficient for leaf only	Max coeffi cients for leaf, sapwood, and root. Actual depend on plant P limi tation	Constant coefficient for leaf and root	Constant coef ficients for leaf, wood, and fine root pools	Same as OCHDP	Same as QUINC
**Plant P uptake**	Function of plant P de mand and soil labile P	Function of plant P de mand and soil solution P	Function of plant P de mand, root C, and inorganic labile P pool	Function of plant P de mand and sta tus, root C, soil mineral P pool, and T. Cohort partitioning based on fine root surface	Function of plant P demand, root C, root uptake capacity, dis solved labile P pool, and soil diffusivity	Function of plant P demand scalar, root C, root up take capacity, soluble P pool, regulated by soil T and moisture	Same as OCHDP	Similar as QUINC, further regulated by competition between soil microbes and mineral surface
**Plant P demand**	Function of growth rates and tissue C: P ratios	Function of growth rate of tissue C:P ratios	Function of growth and tis sue C:P ratios	Function to optimization *V*_cmax_ in leaves (optimal C:P ratio)	Function of growth rates and tissue CP ratios	Function of growth rates and target growth NP ratio, which is dependent on the plant labile N&P pool	Same as OCHDP	Same as QUINC
**Soil P biochemical mineralization**	Dynamic function of soil organic P turnover rate (slow, passive pool)	Function of soil organic P, the extent of N limitation and P limitation	Function of soil organic P turnover rate (slow and passive pool)	Function of soil layer organic P pool (slow pool), PO_4_, temperature, moisture, and root density	Dynamic function of leaf N:P imbalance and substrate availability	Function of soil layer organic P pool (slow pool), temperature, and moisture	Same as OCHDP	Function of P in soil layer organic pool (microbial residue, mineral- associated OC), microbial phosphatase abundance, soil organic pool C:P ratio, T, and moisture
**P desorption of secondary P**	None	Fixed desorp tion rate	Function of soil pH	Function of soil layer tem perature	None	Function of soil temper ature and moisture	Same as OCHDP	Same as QUINC
**P occlusion**	Fixed fraction of strongly sorbed P pool	Fixed occlu sion rate	Fixed fraction of strongly sorbed P pool	Fixed fraction of sorbed P pool	Fixed fraction of labile sorbed P	Fixed fraction of strongly sorbed P pool	Same as OCHDP	Same as QUINC

## RESULTS

### No single model could predict all observed eCO_2_ responses

Phosphorus-enabled models varied in their skills in reproducing the observed C- and P-cycle dynamics under ambient CO_2_ treatment, and they differed in their ability to match the observed sign and magnitude of eCO_2_ responses at EucFACE (Fig. 2). The data-model intercomparison under ambient CO_2_ treatment provides a baseline understanding of model performance, whereas the comparisons of the sign and magnitude of the eCO_2_ predictions evaluate the direction and accuracy of the predicted CO_2_ responses. In general, no single model performed consistently well against the observed CO_2_ effects for all simulated variables (Fig. 2).

Most models were capable of predicting the sign and magnitude of the CO_2_ effect on NEP within the uncertainty bound of the observations (Fig. 2A). However, the predicted CO_2_ effects on NEP varied considerably among models (−4 to 245 g C m^−2^ year^−1^), and overall, they tended to estimate a positive CO_2_ effect (multimodel mean and SD of 129 ± 83 g C m^−2^ year^−1^). In comparison, none of the three independent observation-based datasets of NEP (*23*) indicated a significant CO_2_ effect. Moreover, although model predictions were within the uncertainty bounds of the correct CO_2_ effect on NEP—an aggregate variable that reflects the combined responses of plant C assimilation and a range of return fluxes of C to the atmosphere—they performed less well against observations on the individual component fluxes or on other variables related to the processes controlling these fluxes (Fig. 2, B to E). In sum, although NEP predictions are not inconsistent with data, the underlying process representations leading to the NEP predictions are unlikely to be supported in full for any of the models.

The inclusion of P-cycle processes improved model realism, in that the P-enabled models predicted lower biomass sequestration and CO_2_ responses when compared to their corresponding CN versions (fig. S2). The simulated down-regulation effect of P on growth was in line with P fertilization responses observed in the same forest ecosystem that demonstrates that P availability limits tree growth (*22*). Additions of P-cycle processes also allowed a more explicit and process-oriented approach to simulate the complex ecosystem feedback and interaction (tables S2 to S4). Nonetheless, the multimodel mean and spread of the P-enabled models were not greatly different from the multimodel predictions made in advance of the experiment where most models lacked a P-cycle representation (fig. S3) (*17*). Thus, although addition of P-enabled processes resulted in more comprehensive and mechanistic representations of ecosystem biogeochemistry, it did not reduce overall uncertainty in model predictions in terms of the multimodel spread.

### The predicted plant C uptake was overly sensitive to eCO_2_

Mechanistically, the diverging effects of CO_2_ on NEP across models reflect different embedded assumptions on plant C uptake (Fig. 3), allocation (Fig. 4), and their interaction with P-cycle processes (Figs. 5 and 6). Model estimates of GPP response to eCO_2_ ranged from 5 to 30%, with a multimodel mean of 20%, suggesting a strong CO_2_ fertilization effect on C uptake by trees (Fig. 3, A and C, and fig. S4). The modeled GPP response reflects both leaf physiological (Fig. 3C) and leaf area responses to eCO_2_ (Fig. 3, B and D). Most models (except GDAYP and LPJGP) predicted a lack of response in leaf area index (LAI) to eCO_2_ (Fig. 3, B and D), in line with data (*23*, *24*, *32*). However, only QUJSM predicted the correct magnitude of both GPP and LAI responses to eCO_2_, whereas the other models (except GDAYP) generally overestimated these responses (Fig. 3E). Hence, the multimodel mean response of GPP (~20%) is substantially larger than the responses independently estimated based on site data [i.e., OBS_field_ and OBS_inst_; 6 and 11%, with and without accounting for variation in LAI among treatment plots (*24*); Fig. 3C].

This overestimation of GPP response is unlikely to be related to nutrient cycling assumptions. Most models allowed leaf nutrient concentrations to vary within bounds, resulting in a dilution effect of eCO_2_ on leaf P and N concentrations, which was mostly within the range of data-based uncertainty (fig. S5). Although models incorporated different empirical relationships to represent the leaf nutrient effect on photosynthesis and its response to eCO_2_ (Table 1), the dilution effect of eCO_2_ was small in most models (<5%), consistent with data.

Model assumptions regarding leaf-to-canopy scaling played a larger role in data-model divergence. The predicted strong CO_2_ response is comparable to the 19% light-saturated leaf-level response estimated for the site (*21*). This result suggests that models may have unrealistic representations of the leaf-to-canopy scaling of photosynthesis, especially the relative limitation role of Rubisco (A_c_) and electron transport (A_j_) on canopy photosynthesis (Fig. 3C). In particular, A_c_ limitation appears to dominate the simulated GPP response to eCO_2_ in most models, whereas the site estimates indicate a much lower sensitivity of GPP response to eCO_2_ (i.e., 11% at the canopy scale, with pretreatment differences in leaf area taken into account), and this lower sensitivity is predominately explained by the prevalence of A_j_-limited leaf photosynthesis within the canopy (*24*).

### The CO_2_ effects on plant growth and ecosystem C sequestration were overestimated

Models differed in allocating the extra photosynthates assimilated under eCO_2_, leading to different predictions of plant growth and NEP responses to eCO_2_ (Fig. 4 and fig. S6). GDAYP, ELMV1, LPJGP, QUINC, and QUJSM predicted increases in net primary production (NPP) with eCO_2_ (Fig. 4A). This prediction is generally in line with data, but these models differed with regard to the fate of the extra C along the plant-soil continuum (Fig. 4B). LPJGP predicted that most of this extra C led to a larger annual increment in plant biomass (ΔC_veg_), possibly because of its highly flexible plant stoichiometry (fig. S7). By comparison, ELMV1, QUINC, and QUJSM predicted extra C accumulation in the plant storage pool (fig. S6). Consequently, these models predicted strong CO_2_ fertilization effects on ΔC_veg_ and NEP (Fig. 4, C and D), inconsistent with the observations (*21*, *23*). CABLP, OCHDP, and OCHDX simulated a large eCO_2_-induced increase in plant autotrophic respiration (Fig. 4A), apparently as an algorithmic workaround avoiding unrealistic C accumulation in plant biomass. The data show no evidence for an increase in autotrophic respiration (*23*), demonstrating that this assumption is incorrect. Instead, data from the site point to an increased belowground C allocation into fast turnover pools, possibly via root exudates or mycorrhizal associations, and an enhanced soil R_het_ under eCO_2_ (Fig. 4B and fig. S6) (*23*, *33*–*35*). A similar response has also been observed in other eCO_2_ experiments (*36*, *37*). However, among the models, this response only occurred in GDAYP, and this model was modified deliberately to correctly emulate this site-based observation (*28*). Thus, introducing an assumption of greater belowground allocation flux to stimulate soil microbial activity could improve model capacity to capture plant growth response to eCO_2_ when soil nutrient is limiting.

### Plant P demand and uptake responses to eCO_2_ were underestimated

Despite model estimates of the CO_2_ effect on ΔC_veg_ being generally greater than the observations, all models underestimated the CO_2_ effect on P_dem_ and the annual incremental changes in plant P pool (ΔP_veg_; Fig. 5 and fig. S7). Plant P demand is driven by the annual biomass production (BP) fluxes of the different plant tissue compartments (Fig. 5A), modulated by flexibility in tissue C:P ratios (Fig. 5C). Observations suggest a small increase in P_dem_, largely driven by a small decrease in fine root C:P ratio (*38*, *39*). Two models were clearly inconsistent with this observation: GDAYP and LPJGP exhibited large eCO_2_-induced reductions in P_dem,_ likely due to their highly flexible leaf and fine root C:P ratios (Fig. 5, D and G). In three models (CABLP, QUJSM, and ELMV1), the magnitude of the P_dem_ response to eCO_2_ was near zero, smaller than but not statistically distinguishable from observations (Fig. 5B). In these models, there was little change in either plant C:P ratio (Fig. 5D) or tissue production (Fig. 4D). The three remaining models (OCHDP, OCHDX, and QUINC) showed reasonable agreement with data on the magnitude of the eCO_2_-induced increase in P_dem_ (Fig. 5E) but not via the correct mechanism: They predicted increased ΔC_veg_, rather than changes in stoichiometry (Fig. 5D).

Plant P demand is met by the combination of plant internal P recycling (e.g., leaf P resorption) and uptake fluxes (P_res_ and P_upt_, respectively; fig. S7A). All models assumed fixed plant P resorption coefficients regardless of the CO_2_ treatment (Table 1), and this lack of CO_2_ effect on P_res_ is in line with the empirical evidence from EucFACE (*40*). Thus, model predictions of P_upt_ response to eCO_2_ were similar to those of the P_dem_ responses, in that the eCO_2_-driven increase in P_upt_ was clearly underestimated by two models and possibly underestimated by three more (Fig. 5F and fig. S7).

### Representations of soil C-P responses to eCO_2_ remain a major uncertainty

In all models, P_upt_ is functionally related to both plant P demand and the size of the most readily available soil P pool (Table 1). Soil P supply depends on soil net P mineralization flux (P_net_), which is the net balance between gross P mineralization (biological and biochemical) and microbial immobilization (Fig. 6 and figs. S8 and S9). EucFACE data indicate a tendency for an eCO_2_-induced increase in P_net_, but the effect size is dwarfed by the large range of uncertainty, giving a relatively poor constraint on model predictions and highlighting the need for increased efforts to quantify this flux. Nonetheless, ELMV1, OCHDP, and QUINC predicted the correct sign of CO_2_ effect on P_net_ on average, whereas the other models made the opposite predictions, in that P_net_ reduced with eCO_2_ (Fig. 6A). In LPJGP and GDAYP, plant C:P ratio increased with eCO_2_, resulting in poorer litter quality (Fig. 5D), which increased microbial P immobilization. In GDAYP, the eCO_2_-induced increase in root exudation also led to a higher soil P immobilization rather than greater mineralization due to the stoichiometrically driven demand for additional P in the active soil pool to match the additional C entering this pool (*28*). While the increased belowground allocation is supported experimentally, a new model formulation is required to ensure that it does not drive increased P immobilization in GDAYP.

It has been proposed that more realistic representations of soil microbial activity may be important to capture the P-cycle feedback in models, and this avenue seems promising given the importance of soil microbial competition for P at the site (*39*). However, the two models with more advanced microbial representations (OCHDX and QUJSM) simulated an eCO_2_-induced decrease in P_net_, in the opposite direction of their default models (OCHDP and QUINC) and disagree with the data (Fig. 6B). Both models simulated slightly larger microbial C and P pools under eCO_2_ (C_mic_ and P_mic_; fig. S9), in contrast to the data. The increases in C_mic_ and P_mic_ mean that, in both models, the gross P mineralization (and microbial P uptake) increases under eCO_2_ but P_net_ decreases. This indicates that both models underestimate microbial P limitation in the system. Increasing root exudation would potentially alleviate microbial P stress, likely decreasing P_net_ further. Hence, the missing process may be the role of root exudation in directly stimulating P mineralization, for example, via the release of labile C and acid phosphatase (*41*). Alternatively, the belowground allocation may support mycorrhizal symbiosis to facilitate extra nutrient acquisition and mineralization (*25*, *41*), which was not explicitly considered in any of the models tested here. Therefore, none of the models were capable of reproducing the eCO_2_-induced increase in soil R_het_ (Fig. 6, C and D).

## DISCUSSION

To improve the capability of models to realistically capture ecosystem processes and accurately simulate ecosystem dynamics has been one of the central goals driving development of ecosystem and land surface models (*42*–*44*). The inclusion of P-cycle processes has led to more complex model structures and more comprehensive representations of ecosystem biogeochemistry (*45*–*47*). These are important steps toward adequately accounting for C-P interactions, but to what degree they have so far led to more robust predictions of the global land C sink under future climate change remains unclear (*18*). By testing the ability of a suite of models to simulate the observed CO_2_ responses for a P-limited forest subject to long-term CO_2_ enrichment, this data-model intercomparison provides a novel and comprehensive assessment of the predictive capacity of the current generation P-enabled models. Our results show substantial disagreement among models and inconsistency between models and data. By taking an assumption-based approach (*31*), here we identified a number of key sets of assumptions where EucFACE data can guide future model improvement, namely, leaf-to-canopy scaling of photosynthesis, plant C allocation for nutrient acquisition, plant stoichiometric flexibility, and the belowground processes governing soil nutrient mineralization. The importance and deficiencies of these nutrient-dependent processes in models have been repeatedly demonstrated in previous model-based assessments (*17*, *18*, *48*–*51*), although the main focus has been on N rather than P to reflect the model development history. Our work builds further on these findings, providing concrete recommendations to reduce model uncertainty with a particular focus on C-P interactions.

One key recommendation to modelers is a renewed focus on leaf-to-canopy scaling. Although this topic has been extensively explored in previous research (*52*), it nonetheless remains a major source of uncertainty in the eCO_2_ predictions, both in this study and in previous model-based intercomparisons (*51*). Here, most models predicted a GPP response consistent with a large proportion of photosynthesis limited by Rubisco activity, in contrast to the site-based inference that the electron transport limitation dominates (*24*). This discrepancy likely relates to model assumption regarding the leaf-level ratio of maximum electron transport rate to maximum Rubisco activity (*J*_max_:*V*_cmax_). Empirical evidence indicates that this ratio is reduced in plants growing in low-P soils (*53*). This empirical relationship was incorporated into some of the models we tested (e.g., OCHDP), but these models still predicted strong CO_2_ effects on GPP at EucFACE (Fig. 3). Empirical data also suggest a small increase in this ratio with eCO_2_, possibly linked to a dilution effect of leaf P or a reallocation of N away from Rubisco, but the evidence from EucFACE suggests that this reduction has only a small effect at the canopy scale (*24*). The critical assumption may therefore be the way in which leaf-level limitations to photosynthesis are scaled to the canopy (*52*), and our study indicates the need for this assumption to be reexamined.

A second area for model improvement is plant stoichiometric flexibility, which has been repeatedly identified as a key mechanism to enable a positive CO_2_ effect on productivity under nutrient limitation (*18*, *47*, *54*). In general, growth in models is constrained by empirical C:N:P ratios, with higher flexibility leading to a larger growth response to eCO_2_. Analogous with previous studies (*31*, *51*), our work identified discrepancy in the stoichiometric flexibility of eCO_2_ responses between observations and model simulations. Here, we show that the two models with the highest stoichiometric flexibility (GDAYP and LPJGP) predicted an increase in plant C:P ratio, beyond the range of uncertainty in the experimental evidence. It is possible that plants subject to long-term adaptation to P-deprived soils may have limited capacity to further increase their C:P ratios in response to eCO_2_. However, this does not mean that it would be logical to assume a fixed stoichiometry in models; instead, our results suggest a more stringent upper bound for the C:P ratio of plant tissues, especially for the aboveground component. It is observed that the largest change in C:P ratio was in the fine root, but none of the models reproduced this eCO_2_-induced decrease in fine root C:P ratio. Thus, as previously suggested (*31*), this data-model discrepancy indicates the need to include a more appropriate representation of the functional trade-off governing nutrient allocation in plant tissues, which relies on more concrete experimental evidence.

Our results also indicated the need to incorporate an increased belowground allocation pathway to short turnover pools of soil organic matter under eCO_2_ (Fig. 4). Most models assumed one of the three major pathways to “expend” the additional photosynthetic C uptake (i.e., autotrophic respiration, growth, or storage). All of these potential responses are ruled out by observational data at the site (*23*). Instead, the major pathway for additional C was increased belowground allocation in the possible form of flux to mycorrhiza, associated with higher R_het_ (*23*, *33*). Observations from other FACE experiments also support such a belowground allocation pathway (priming effect) (*36*, *37*). In comparison, some models do simulate increased belowground BP under eCO_2_ (*18*), but they typically do not simulate root exudates. While both pathways could result in increased plant nutrient acquisition under eCO_2_, they would lead to different consequences for microbial C-use efficiency, soil organic matter stability, and nutrient acquisition efficiency, with different resulting effects on NEP and ecosystem C storage (*25*, *41*, *47*, *55*).

Consequences of the increased belowground C flux under eCO_2_ should form a key focus for further model development and experimental research. The belowground allocation pathway better connects the C-nutrient feedback between plants and microbes (*28*, *56*). This flux may be part of an active plant strategy under eCO_2_, which has been observed or inferred from eCO_2_ experiments in both N-limited (*36*) and P-limited ecosystems (*23*). Such a process may be particularly adaptive in P-limited ecosystems, where a large fraction of soil P exists in the less available forms (e.g., organic P) and could potentially be remobilized via this process (e.g., inorganic P desorption or organic P mineralization) (*57*). Recent studies using the Fixation and Uptake of Nitrogen (FUN) model have demonstrated that this C-cost effect could provide an effective pathway for plant nutrient acquisition (*58*, *59*). However, most models tested here are not adequate in representing this process (Fig. 6, B and D). Thus, a suggested avenue for model improvement is to include direct effects of C flux on P availability and uptake to plants via phosphatases mineralizing organic P or via mycorrhizal symbiosis (*41*). Additional empirical data are critically needed to understand the extent to which this C flux would affect soil nutrient availability in the presence of microbial competition and how it, in turn, would affect ecosystem C sequestration. Microbially explicit models may be considered helpful in this regard, but more generalizable evidence on the C-nutrient feedback between plant and microbes is needed to better characterize their functional dependence and possible responses to eCO_2_.

Last, our results highlight the benefits of an integrated data-model intercomparison approach as part and parcel of a long-term ecosystem experiment. The a priori predictions made using a suite of models at the outset of the experiment (*17*) enabled more targeted data collections, providing critical information to constrain models in the subsequent data-model intercomparison. A further stage of the experiment is now underway, in which experimental plots are being fertilized with additional P, and models have once again been used to predict the outcome, guiding the focus and scope of observations needed to evaluate the predictions. Previous data-model (*48*–*51*, *60*) or multimodel intercomparisons (*17*, *18*) have been hampered by the lack of integration between models and experimentation. We strongly advocate an iterative data-model intercomparison framework, in which data-model intercomparison works in tandem with data collection. This activity could be considered as part of the global efforts that use a suite of best available in situ, remote sensing and reanalysis datasets to evaluate model performance, essentially allowing models to carry different weight toward the multimodel mean based on their capacity to reproduce the observations [e.g., ILAMB (*61*)]. Such an integrated framework is an invaluable approach to advance the predictive capacity of process-based models when addressing future scenarios of climate change.

In conclusion, this data-model intercomparison provides an important test to understand the predictions of the land C sink under rising CO_2_ made by the P-enabled models. The P limitation of forest productivity at EucFACE can be considered broadly representative of forests growing on P-poor soils globally (*39*), including extensive parts of the moist tropics and low-latitude drylands (*7*). As such, the model deficiencies identified here highlight crucial model-based uncertainties regarding the C sequestration potential of low-P forests under rising CO_2_. In particular, we find that the models are generally overly sensitive to eCO_2_ in their C uptake and sequestration predictions. It is thus possible that model estimates of the CO_2_ fertilization driver of the future land C sink may be overestimated, although P limitation has already reduced the magnitude of the CO_2_ effect when compared to simulations without P cycle processes (*4*, *15*, *16*). Hence, climate change mitigation strategies that rely on a strong CO_2_ fertilization effect as a major future driver of increased land C sink should be considered with caution. Nonetheless, it is necessary for future model development and experimentation to resolve the process-based discrepancies identified in this study. Our work represents a solid step forward and will contribute to a more concrete prediction of the land C sink in the context of the global C balance under climate change.

## MATERIALS AND METHODS

### Site and experimental data description

The EucFACE experiment is located in a mature evergreen *Eucalyptus* forest on phosphorus-deprived alluvial spodosol soils near Sydney, Australia (33°36′S, 150°44′E). The site is characterized by a humid temperate–subtropical transitional climate with a mean annual temperature of 17.5°C and a mean annual precipitation of 800 mm. The site has remained unmanaged for at least over 90 years and is dominated by *Eucalyptus tereticornis* Sm. in the overstorey. The understorey is dominated by the C3 perennial grass *Microlaena stipoides*. Six circular plots of 490 m^2^ each were established for the FACE experiment, with three subject to CO_2_ enrichment of +150 parts per million (ppm) starting from 6 February 2013 during daylight hours on all days of the year (i.e., *n* = 3) (*33*).

EucFACE provides long-term, in situ and ecosystem-scale experimental data of ecosystem dynamics under both ambient and eCO_2_ treatment. We compiled a site-specific, high-frequency (half-hourly and daily), time-series meteorological dataset over the period of 2012 to 2018 to drive the model simulation. We synthesized comprehensive C (*23*) and P budgets (*39*) covering major plant and soil pools and fluxes over the period of 2012 to 2018 to parameterize and evaluate the model performance (details in Modeling protocol and Analysis and Supplementary Information sections 2, 3, and 4). Detailed interpretations to these observations have been provided elsewhere [e.g., (*21*, *23*, *32*–*35*, *39*, *40*)], and therefore in this study, we only focus on reporting the data-model comparisons.

### Model descriptions

#### Overview

This data-model intercomparison includes six state-of-the-art, P-enabled terrestrial ecosystem models, two of which have the additional capacity to simulate microbial processes by coupling to their corresponding microbial submodules (Supplementary Information section 1, tables S1 to S6, and figs. S10 to S17). The selection of this list of models takes into consideration the P-enabled models available at the time this work started, the knowledge gathered from the previous multimodel intercomparison works (*17*, *18*), and the principle to include a variety of model-based assumptions to compare with data. The models are a stand-scale ecosystem model GDAY [Generic Decomposition And Yield model (*27*, *28*); abbreviated in this study as GDAYP], five land surface models CABLE-POP [Community Atmosphere Biosphere Land Emulator coupled with the Populations-Order-Physiology module simulating woody demography but with POP switched off in this study (*10*); abbreviated as CABLP], ELM [Energy Exascale Earth System Model land model v1 (*20*); abbreviated as ELMV1], LPJ-GUESS [Lund-Potsdam-Jena General Ecosystem Simulator (*29*); abbreviated as LPJGP], ORCHIDEE-CNP [ORganizing Carbon and Hydrology in Dynamic Ecosystems, version 1.2 (*11*, *30*); abbreviated as ORCHD], and QUINCY [QUantifying the effects of INteracting nutrient CYcles on terrestrial biosphere dynamics and their climate feedbacks (*13*); abbreviated as QUINC for the P-enabled model]. The microbial-coupled models are ORCHIDEE-CNP coupled with a Microbial-Mineral Carbon Stabilization (MIMICS) model-type (*30*, *62*) microbial module (abbreviated as OCHDX) and QUINCY coupled with the Jena Soil Model (*19*) (abbreviated as QUJSM). All eight models include C-, N-, and water-cycle processes, and all eight models include a prognostic P cycle but with different degrees of detail and mechanistic assumptions on plant and soil processes (Supplementary Information section 1). To quantify the effect of the P cycle alone, we ran a subset of the models, GDAY and LPJ-GUESS, without the P cycle turned on (abbreviated as GDAYN and LPJGN) and made model-specific comparisons (fig. S2). This approach is different from the previous modeling work (*18*) where the ensemble means of CN and CNP models were compared. We suggest that our model-specific comparison may be more useful because it isolates the effect of the P cycle for these models rather than structural differences among different models.

#### Model structure

Models included in this study share common representation of vegetation and soil structure (Supplementary Information section 1 and tables S2 to S6). In short, all models have fast turnover leaf and fine root pools and at least one slow turnover plant tissue pool to represent the woody component. In addition, models generally implement at least one nonstructural carbohydrate pool as a way to store that excess C that is not immediately used for plant growth (e.g., CABLP, ELMV1, ORCHD, and QUINC). For the representation of soil organic matter, all default models assume a multipool structure, with turnover rates varying across the pools and controlled by soil physical factors such as temperature, moisture, and/or clay content. The microbial-explicit models have additional complexities that are described in the section Microbial dynamics. Some models have vertically resolved soil profile, including biogeochemistry (e.g., QUINC and LPJGP).

#### Plant physiology, allocation, and growth

Models differ in their representation of plant photosynthesis (Supplementary Information section 1). Most models adopted the Farquhar formulation for photosynthesis (e.g., CABLP, ELMV1, GDAYP, OCHDP, and OCHDX) (*63*), but there are additional variations of this form. For example, CABLP additionally implemented a coordination theory where canopy-level photosynthesis is colimited by *V*_cmax_ and *J*_max_ (*10*), and LPJGP implemented the Collatz formulation (*64*), while QUINC implemented the Kull and Kruijt relationship (*65*). Nutrient limitation on photosynthesis is realized via leaf tissue nutrient effect on photosynthetic capacity, but the exact forms of this relationship vary among models (Table 1). Some assumed no direct effect of leaf P [e.g., QUINC (*65*, *66*)], while others assumed direct regulation of *V*_cmax_ (maximum rate for carboxylation) and/or *J*_max_ (maximum rate for electron transport) via the dynamics of leaf N and P, but the exact form of this relationship varies (*8*, *53*, *67*).

For plant C allocation, models generally adopt functional allometric relationships, which subsequently depend on nutrient availability (Supplementary Information section 1 and Table 1). CABLP assumed fixed allocation fractions to leaf, wood, and root. Extra C acquired by plants under eCO_2_ not used for additional growth could be lost via autotrophic respiration (CABLP, QUINC, and QUJSM), stored in plants as nonstructural carbohydrates or is respired (OCHDP and OCHDX), or allocated into soil as root exudates [GDAYP (*28*)]. Plant growth is determined by the relative limitation of N and P in most models (i.e., the Liebig’s law of minimum approach). Because most models consider nonstructural carbohydrates as part of plant biomass, NPP equals BP.

#### Phosphorus cycling

Because this work focuses on the P cycle, here we only describe the P cycle not the N cycle. An overview of the major model-based assumptions for the N cycle is available in Supplementary Information section 1 (especially table S4).

Soil P is represented in the models as pools of different bioavailability, e.g., inferred based on the soil Hedley fractionation method (*68*), with the number of pools varying among models (Table 1). In general, plants take up P from the most labile soil P pool (e.g., solution P pool as in ELMV1 and labile P pool as in GDAYP), and this pool is in dynamic equilibrium with a sorbed P pool (*8*). A large proportion of inorganic P is locked up in less available forms, e.g., occluded P pool, and most models assume that this pool does not release P back to the more available pools. Soil P mineralization occurs in two forms: biochemical and biological P mineralization, with biological P mineralization typically follow the same assumption as N mineralization (i.e., the net balance between gross mineralization and immobilization as driven by microbial activities), whereas biochemical P mineralization typically relates to phosphatase production and, in some models, the N costs of P uptake (*8*).

Plant P uptake is generally represented in models as a function of plant nutrient demand, root size, and soil nutrient availability, but there are more advanced model forms, such as those additional driven by the competition between soil microbes and mineral surface (i.e., QUJSM), and soil P diffusion (OCHDP and OCHDX). Plant P demand is driven by plant production and the tissue-specific CP ratios, which vary among models. All models represent plant P resorption using a predetermined fixed rate that varies among models (Table 1). LPJGP further assumes that the actual rates of resorption depend on plant P stress. Most models consider plant nutrient resorption for the leaf, wood, and root pools, but some only consider leaf (e.g., GDAYP).

#### Microbial dynamics

Microbial processes are explicitly represented by OCHDX and QUJSM, with different assumptions (Supplementary Information section 1). OCHDX implemented a MIMICS-type microbial scheme (*62*) that splits soil microbes into two different strategy groups that compete for resources with varying carbon use efficiency dynamics. In comparison, QUJSM incorporates representation of enzyme allocation to different depolymerization sources based on the microbial adaptation approach as well as that of nutrient acquisition competition based on the equilibrium chemistry approximation approach (*19*). Both models assume nonlinear decomposition rates of organic matter, which are regulated by the microbial biomass. The microbial growth is limited by the availability of C, N, and P. Microbes can adjust their carbon use efficiency (CUE) in response to changes of available C or nutrients. In the P-deprived soil, QUJSM can increase the production of phosphatase to mobilize P from more stable SOM, which also benefits plant P acquisitions.

### Modeling protocol

All models followed the same modeling protocol. Model spin-up was based on the randomized, repeated meteorological forcing data collected from the site over the period of 2013 to 2018, under a preindustrial atmospheric CO_2_ concentration of 280 ppm. We ran models over the period of 1750 to 2012 to build up the vegetation and soil pools with the same randomized, repeated meteorological forcing under transient historic CO_2_ and N and P deposition (*69*). Because the site is a mature forest that remained unmanaged for, at least, the past 90 years, we did not impose any land use change scenarios in the modeling. We then forced the models with site-specific, time sequence meteorological data under both ambient and eCO_2_ concentrations over the experimental period of 2013 to 2018. The details of the modeling protocol are available in Supplementary Information section 2 (especially table S7).

A set of site-based observational dataset under ambient CO_2_ treatment was provided to modelers (Supplementary Information section 3 and tables S8 to S17) so that models can parameterize their respective plant functional type that is most appropriate to represent the dominant tree species at EucFACE (i.e., broadleaf evergreen tree *E. tereticornis*). This set of observational dataset covers all major ecosystem variables, and therefore models were relatively well constrained in terms of measurable parameters under the ambient CO_2_ treatment. Each model was calibrated separately, based on the model’s specific calibration procedure, but partner models (i.e., GDAYN and GDAYP, LPJGN and LPJGP, QUINC and QUJSM, and OCHDP and OCHDX) shared commonality in parameters. For example, QUINC and QUJSM used the same parameters except the soil component, where they were based on different submodules. Hence, although models were provided with the same benchmarking dataset, they may still differ in tunable parameters that are not directly measurable, especially for those that are specific to each model. However, given that this study focuses on evaluating model assumptions regarding the underpinning ecological processes, we did not perform a systematic sensitivity test on model parameters and acknowledge that this lacking represents a potential caveat of this study. Model output protocols are available in Supplementary Information section 4 (especially tables S18 and S19). We then checked the mass balance of all essential C, N, P, and water fluxes and pools, and all models passed these quality controls and therefore were included in this study.

### Analysis

We evaluated predictions made by these P-enabled models against data, with a particular focus on their ability to accurately predict the CO_2_ responses as observed at EucFACE. We acknowledged the possibility that models could yield quantitatively good predictions based on incorrect underlying mechanisms (*42*). We therefore adopted an assumption-centric approach (*31*) to investigate if the underlying mechanisms leading to the prediction are in broad agreement with those revealed by the data (Fig. 1). We focused our evaluations on individual models over multimodel means because the latter typically do not reveal process-based uncertainties that are specific to each model, but we still calculated the multimodel means and their associated uncertainties (i.e., SD of the multimodel prediction, *n* = 8). We reported the observational means and uncertainties at the treatment level, i.e., calculating the means and SDs based on data collected from the three ambient and eCO_2_ plots (*n* = 3).

Moreover, a set of model simulations was made available in advance of the EucFACE experiment (*17*). This a priori prediction provided some likely trajectories of the ecosystem responses to planned CO_2_ enrichment at the site based on plausible model-based assumptions and hypothetical meteorological forcing data (i.e., wet-year fixed climate with daily variation). These model simulations included 6 CN coupled models and 2 CNP models. Our work differed to that of (*17*) in that this work included more P-enabled models to reflect the recent community efforts in incorporating P-cycle processes into models and that models in this work were provided with site-specific datasets under ambient CO_2_ treatment for parameterization purpose. Here, we briefly compared the two simulations to understand if the inclusion of P-cycle processes into more models can reduce the multimodel uncertainty as reflected by the spread of the multimodel predictions (fig. S3).

## Supplementary Materials

### This PDF file includes:

Supplementary Text Figs. S1 to S17 Tables S1 to S19 References

## References

[1] V. K. Arora, A. Katavouta, R. G. Williams, C. D. Jones, V. Brovkin, P. Friedlingstein, J. Schwinger, L. Bopp, O. Boucher, P. Cadule, M. A. Chamberlain, J. R. Christian, C. Delire, R. A. Fisher, T. Hajima, T. Ilyina, E. Joetzjer, M. Kawamiya, C. D. Koven, J. P. Krasting, R. M. Law, D. M. Lawrence, A. Lenton, K. Lindsay, J. Pongratz, T. Raddatz, R. Séférian, K. Tachiiri, J. F. Tjiputra, A. Wiltshire, T. Wu, T. Ziehn, Carbon–concentration and carbon–climate feedbacks in CMIP6 models and their comparison to CMIP5 models. Biogeosciences 17, 4173–4222 (2020).

[2] P. Friedlingstein, M. W. Jones, M. O’Sullivan, R. M. Andrew, D. C. E. Bakker, J. Hauck, C. Le Quéré, G. P. Peters, W. Peters, J. Pongratz, S. Sitch, J. G. Canadell, P. Ciais, R. B. Jackson, S. R. Alin, P. Anthoni, N. R. Bates, M. Becker, N. Bellouin, L. Bopp, T. T. T. Chau, F. Chevallier, L. P. Chini, M. Cronin, K. I. Currie, B. Decharme, L. M. Djeutchouang, X. Dou, W. Evans, R. A. Feely, L. Feng, T. Gasser, D. Gilfillan, T. Gkritzalis, G. Grassi, L. Gregor, N. Gruber, Ö. Gürses, I. Harris, R. A. Houghton, G. C. Hurtt, Y. Iida, T. Ilyina, I. T. Luijkx, A. Jain, S. D. Jones, E. Kato, D. Kennedy, K. Klein Goldewijk, J. Knauer, J. I. Korsbakken, A. Körtzinger, P. Landschützer, S. K. Lauvset, N. Lefèvre, S. Lienert, J. Liu, G. Marland, P. C. McGuire, J. R. Melton, D. R. Munro, J. E. M. S. Nabel, S.-I. Nakaoka, Y. Niwa, T. Ono, D. Pierrot, B. Poulter, G. Rehder, L. Resplandy, E. Robertson, C. Rödenbeck, T. M. Rosan, J. Schwinger, C. Schwingshackl, R. Séférian, A. J. Sutton, C. Sweeney, T. Tanhua, P. P. Tans, H. Tian, B. Tilbrook, F. Tubiello, G. R. van der Werf, N. Vuichard, C. Wada, R. Wanninkhof, A. J. Watson, D. Willis, A. J. Wiltshire, W. Yuan, C. Yue, X. Yue, S. Zaehle, J. Zeng, Global Carbon Budget 2021. Earth Syst. Sci. Data 14, 1917–2005 (2022).

[3] W. R. Wieder, C. C. Cleveland, W. K. Smith, K. Todd-Brown, Future productivity and carbon storage limited by terrestrial nutrient availability. Nat. Geosci. 8, 441–444 (2015).

[4] Q. Zhang, Y. P. Wang, R. J. Matear, A. J. Pitman, Y. J. Dai, Nitrogen and phosphorous limitations significantly reduce future allowable CO_2_ emissions. Geophys. Res. Lett. 41, 632–637 (2014).

[5] M. Hawkesford, I. Cakmak, D. Coskun, L. J. De Kok, H. Lambers, J. K. Schjoerring, P. J. White, Chapter 6: Functions of macronutrients in *Marschner’s Mineral Nutrition of Plants* (Elsevier, ed. 4, 2023), pp. 201–228.

[6] E. Hou, Y. Luo, Y. Kuang, C. Chen, X. Lu, L. Jiang, X. Luo, D. Wen, Global meta-analysis shows pervasive phosphorus limitation of aboveground plant production in natural terrestrial ecosystems. Nat. Commun. 11, 637 (2020). 32005808 10.1038/s41467-020-14492-w PMC6994524

[7] E. Du, C. Terrer, A. F. A. Pellegrini, A. Ahlström, C. J. van Lissa, X. Zhao, N. Xia, X. Wu, R. B. Jackson, Global patterns of terrestrial nitrogen and phosphorus limitation. Nat. Geosci. 13, 221–226 (2020).

[8] Y.-P. Wang, B. Z. Houlton, C. B. Field, A model of biogeochemical cycles of carbon, nitrogen, and phosphorus including symbiotic nitrogen fixation and phosphatase production. Global Biogeochem. Cycles 21, GB1018 (2007).

[9] X. Yang, P. E. Thornton, D. M. Ricciuto, W. M. Post, The role of phosphorus dynamics in tropical forests—A modeling study using CLM-CNP. Biogeosciences 11, 1667–1681 (2014).

[10] V. Haverd, B. Smith, L. Nieradzik, P. R. Briggs, W. Woodgate, C. M. Trudinger, J. G. Canadell, M. Cuntz, A new version of the CABLE land surface model (Subversion revision r4601) incorporating land use and land cover change, woody vegetation demography, and a novel optimisation-based approach to plant coordination of photosynthesis. Geosci. Model Dev. 11, 2995–3026 (2018).

[11] D. S. Goll, N. Vuichard, F. Maignan, A. Jornet-Puig, J. Sardans, A. Violette, S. Peng, Y. Sun, M. Kvakic, M. Guimberteau, B. Guenet, S. Zaehle, J. Penuelas, I. Janssens, P. Ciais, A representation of the phosphorus cycle for ORCHIDEE (revision 4520). Geosci. Model Dev. 10, 3745–3770 (2017).

[12] Q. Zhu, W. J. Riley, J. Tang, N. Collier, F. M. Hoffman, X. Yang, G. Bisht, Representing nitrogen, phosphorus, and carbon interactions in the E3SM land model: Development and global benchmarking. J. Adv. Model. Earth Syst. 11, 2238–2258 (2019).

[13] T. Thum, S. Caldararu, J. Engel, M. Kern, M. Pallandt, R. Schnur, L. Yu, S. Zaehle, A new model of the coupled carbon, nitrogen, and phosphorus cycles in the terrestrial biosphere (QUINCY v1.0; revision 1996). Geosci. Model Dev. 12, 4781–4802 (2019).

[14] Q. Zhang, Y. P. Wang, A. J. Pitman, Y. J. Dai, Limitations of nitrogen and phosphorous on the terrestrial carbon uptake in the 20th century. Geophys. Res. Lett. 38, L22701 (2011).

[15] D. S. Goll, V. Brovkin, B. R. Parida, C. H. Reick, J. Kattge, P. B. Reich, P. M. van Bodegom, Ü. Niinemets, Nutrient limitation reduces land carbon uptake in simulations with a model of combined carbon, nitrogen and phosphorus cycling. Biogeosciences 9, 3547–3569 (2012).

[16] T. Ziehn, Y.-P. Wang, Y. Huang, Land carbon-concentration and carbon-climate feedbacks are significantly reduced by nitrogen and phosphorus limitation. Environ. Res. Lett. 16, 074043 (2021).

[17] B. E. Medlyn, M. G. De Kauwe, S. Zaehle, A. P. Walker, R. A. Duursma, K. Luus, M. Mishurov, B. Pak, B. Smith, Y. Wang, X. Yang, K. Y. Crous, J. E. Drake, T. E. Gimeno, C. A. Macdonald, R. J. Norby, S. A. Power, M. G. Tjoelker, D. S. Ellsworth, Using models to guide field experiments: A priori predictions for the CO_2_ response of a nutrient- and water-limited native Eucalypt woodland. Glob. Chang. Biol. 22, 2834–2851 (2016). 26946185 10.1111/gcb.13268

[18] K. Fleischer, A. Rammig, M. G. De Kauwe, A. P. Walker, T. F. Domingues, L. Fuchslueger, S. Garcia, D. S. Goll, A. Grandis, M. Jiang, V. Haverd, F. Hofhansl, J. A. Holm, B. Kruijt, F. Leung, B. E. Medlyn, L. M. Mercado, R. J. Norby, B. Pak, C. von Randow, C. A. Quesada, K. J. Schaap, O. J. Valverde-Barrantes, Y.-P. Wang, X. Yang, S. Zaehle, Q. Zhu, D. M. Lapola, Amazon forest response to CO_2_ fertilization dependent on plant phosphorus acquisition. Nat. Geosci. 12, 736–741 (2019).

[19] L. Yu, B. Ahrens, T. Wutzler, M. Schrumpf, S. Zaehle, Jena Soil Model (JSM v1.0; revision 1934): A microbial soil organic carbon model integrated with nitrogen and phosphorus processes. Geosci. Model Dev. 13, 783–803 (2020).

[20] X. Yang, D. M. Ricciuto, P. E. Thornton, X. Shi, M. Xu, F. Hoffman, R. J. Norby, The effects of phosphorus cycle dynamics on carbon sources and sinks in the Amazon region: A modeling study using ELM v1. J. Geophys. Res. Biogeo. 124, 3686–3698 (2019).

[21] D. S. Ellsworth, I. C. Anderson, K. Y. Crous, J. Cooke, J. E. Drake, A. N. Gherlenda, T. E. Gimeno, C. A. Macdonald, B. E. Medlyn, J. R. Powell, M. G. Tjoelker, P. B. Reich, Elevated CO_2_ does not increase eucalypt forest productivity on a low-phosphorus soil. Nat. Clim. Change 7, 279–282 (2017).

[22] K. Y. Crous, A. Ósvaldsson, D. S. Ellsworth, Is phosphorus limiting in a mature Eucalyptus woodland? Phosphorus fertilisation stimulates stem growth. Plant Soil 391, 293–305 (2015).

[23] M. Jiang, B. E. Medlyn, J. E. Drake, R. A. Duursma, I. C. Anderson, C. V. M. Barton, M. M. Boer, Y. Carrillo, L. Castañeda-Gómez, L. Collins, K. Y. Crous, M. G. De Kauwe, B. M. dos Santos, K. M. Emmerson, S. L. Facey, A. N. Gherlenda, T. E. Gimeno, S. Hasegawa, S. N. Johnson, A. Kännaste, C. A. Macdonald, K. Mahmud, B. D. Moore, L. Nazaries, E. H. J. Neilson, U. N. Nielsen, Ü. Niinemets, N. J. Noh, R. Ochoa-Hueso, V. S. Pathare, E. Pendall, J. Pihlblad, J. Piñeiro, J. R. Powell, S. A. Power, P. B. Reich, A. A. Renchon, M. Riegler, R. Rinnan, P. D. Rymer, R. L. Salomón, B. K. Singh, B. Smith, M. G. Tjoelker, J. K. M. Walker, A. Wujeska-Klause, J. Yang, S. Zaehle, D. S. Ellsworth, The fate of carbon in a mature forest under carbon dioxide enrichment. Nature 580, 227–231 (2020). 32269351 10.1038/s41586-020-2128-9

[24] J. Yang, B. E. Medlyn, M. G. De Kauwe, R. A. Duursma, M. Jiang, D. Kumarathunge, K. Y. Crous, T. E. Gimeno, A. Wujeska-Klause, D. S. Ellsworth, Low sensitivity of gross primary production to elevated CO_2_ in a mature eucalypt woodland. Biogeosciences 17, 265–279 (2020).

[25] C. E. Prescott, S. J. Grayston, H.-S. Helmisaari, E. Kaštovská, C. Körner, H. Lambers, I. C. Meier, P. Millard, I. Ostonen, Surplus carbon drives allocation and plant–soil interactions. Trends Ecol. Evol. 35, 1110–1118 (2020). 32928565 10.1016/j.tree.2020.08.007

[26] L. Castañeda-Gómez, J. K. M. Walker, J. R. Powell, D. S. Ellsworth, E. Pendall, Y. Carrillo, Impacts of elevated carbon dioxide on carbon gains and losses from soil and associated microbes in a Eucalyptus woodland. Soil Biol. Biochem. 143, 107734 (2020).

[27] H. N. Comins, R. E. McMurtrie, Long-term response of nutrient-limited forests to CO_2_ enrichment; equilibrium behavior of plant-soil models. Ecol. Appl. 3, 666–681 (1993). 27759289 10.2307/1942099

[28] M. Jiang, S. Zaehle, M. G. De Kauwe, A. P. Walker, S. Caldararu, D. S. Ellsworth, B. E. Medlyn, The quasi-equilibrium framework revisited: Analyzing long-term CO_2_ enrichment responses in plant–soil models. Geosci. Model Dev. 12, 2069–2089 (2019).

[29] B. Smith, D. Wårlind, A. Arneth, T. Hickler, P. Leadley, J. Siltberg, S. Zaehle, Implications of incorporating N cycling and N limitations on primary production in an individual-based dynamic vegetation model. Biogeosciences 11, 2027–2054 (2014).

[30] H. Zhang, D. S. Goll, Y. Wang, P. Ciais, W. R. Wieder, R. Abramoff, Y. Huang, B. Guenet, A. Prescher, R. A. Viscarra Rossel, P. Barré, C. Chenu, G. Zhou, X. Tang, Microbial dynamics and soil physicochemical properties explain large-scale variations in soil organic carbon. Glob. Chang. Biol. 26, 2668–2685 (2020). 31926046 10.1111/gcb.14994

[31] B. E. Medlyn, S. Zaehle, M. G. De Kauwe, A. P. Walker, M. C. Dietze, P. J. Hanson, T. Hickler, A. K. Jain, Y. Luo, W. Parton, I. C. Prentice, P. E. Thornton, S. Wang, Y.-P. Wang, E. Weng, C. M. Iversen, H. R. McCarthy, J. M. Warren, R. Oren, R. J. Norby, Using ecosystem experiments to improve vegetation models. Nat. Clim. Change 5, 528–534 (2015).

[32] R. A. Duursma, T. E. Gimeno, M. M. Boer, K. Y. Crous, M. G. Tjoelker, D. S. Ellsworth, Canopy leaf area of a mature evergreen Eucalyptus woodland does not respond to elevated atmospheric [CO_2_] but tracks water availability. Glob. Chang. Biol. 22, 1666–1676 (2016). 26546378 10.1111/gcb.13151

[33] J. E. Drake, C. A. Macdonald, M. G. Tjoelker, K. Y. Crous, T. E. Gimeno, B. K. Singh, P. B. Reich, I. C. Anderson, D. S. Ellsworth, Short-term carbon cycling responses of a mature eucalypt woodland to gradual stepwise enrichment of atmospheric CO_2_ concentration. Glob. Chang. Biol. 22, 380–390 (2016). 26426394 10.1111/gcb.13109

[34] S. Hasegawa, C. A. Macdonald, S. A. Power, Elevated carbon dioxide increases soil nitrogen and phosphorus availability in a phosphorus-limited Eucalyptus woodland. Glob. Chang. Biol. 22, 1628–1643 (2016). 26546164 10.1111/gcb.13147

[35] R. Ochoa-Hueso, J. Hughes, M. Delgado-Baquerizo, J. E. Drake, M. G. Tjoelker, J. Piñeiro, S. A. Power, Rhizosphere-driven increase in nitrogen and phosphorus availability under elevated atmospheric CO_2_ in a mature Eucalyptus woodland. Plant Soil 416, 283–295 (2017).

[36] J. E. Drake, A. Gallet-Budynek, K. S. Hofmockel, E. S. Bernhardt, S. A. Billings, R. B. Jackson, K. S. Johnsen, J. Lichter, H. R. McCarthy, M. L. McCormack, D. J. P. Moore, R. Oren, S. Palmroth, R. P. Phillips, J. S. Pippen, S. G. Pritchard, K. K. Treseder, W. H. Schlesinger, E. H. DeLucia, A. C. Finzi, Increases in the flux of carbon belowground stimulate nitrogen uptake and sustain the long-term enhancement of forest productivity under elevated CO_2_. Ecol. Lett. 14, 349–357 (2011). 21303437 10.1111/j.1461-0248.2011.01593.x

[37] R. P. Phillips, I. C. Meier, E. S. Bernhardt, A. S. Grandy, K. Wickings, A. C. Finzi, Roots and fungi accelerate carbon and nitrogen cycling in forests exposed to elevated CO_2_. Ecol. Lett. 15, 1042–1049 (2012). 22776588 10.1111/j.1461-0248.2012.01827.x

[38] J. Piñeiro, R. Ochoa-Hueso, J. E. Drake, M. G. Tjoelker, S. A. Power, Water availability drives fine root dynamics in a Eucalyptus woodland under elevated atmospheric CO_2_ concentration. Funct. Ecol. 34, 2389–2402 (2020).

[39] M. Jiang, K. Y. Crous, Y. Carrillo et al, Microbial competition for phosphorus limits CO_2_ response of a mature forest. Nature 630, 660–665 (2024). 10.1038/s41586-024-07491-0 38839955 PMC11186757

[40] K. Y. Crous, A. Wujeska-Klause, M. Jiang, B. E. Medlyn, D. S. Ellsworth, Nitrogen and phosphorus retranslocation of leaves and stemwood in a mature Eucalyptus forest exposed to 5 years of elevated CO_2_. Front. Plant Sci. 10, 664 (2019). 31214212 10.3389/fpls.2019.00664 PMC6554339

[41] T. Reichert, A. Rammig, L. Fuchslueger, L. F. Lugli, C. A. Quesada, K. Fleischer, Plant phosphorus-use and -acquisition strategies in Amazonia. New Phytol. 234, 1126–1143 (2022). 35060130 10.1111/nph.17985

[42] I. C. Prentice, X. Liang, B. E. Medlyn, Y.-P. Wang, Reliable, robust and realistic: The three R’s of next-generation land-surface modelling. Atmos. Chem. Phys. 15, 5987–6005 (2015).

[43] R. A. Fisher, C. D. Koven, Perspectives on the future of land surface models and the challenges of representing complex terrestrial systems. J. Adv. Model. Earth Syst. 12, e2018MS001453 (2020).

[44] Y. Luo, T. F. Keenan, M. Smith, Predictability of the terrestrial carbon cycle. Glob. Chang. Biol. 21, 1737–1751 (2015). 25327167 10.1111/gcb.12766

[45] S. C. Reed, X. Yang, P. E. Thornton, Incorporating phosphorus cycling into global modeling efforts: A worthwhile, tractable endeavor. New Phytol. 208, 324–329 (2015). 26115197 10.1111/nph.13521

[46] D. L. Achat, L. Augusto, A. Gallet-Budynek, D. Loustau, Future challenges in coupled C–N–P cycle models for terrestrial ecosystems under global change: A review. Biogeochemistry 131, 173–202 (2016).

[47] M. Jiang, S. Caldararu, S. Zaehle, D. S. Ellsworth, B. E. Medlyn, Towards a more physiological representation of vegetation phosphorus processes in land surface models. New Phytol. 222, 1223–1229 (2019). 30659603 10.1111/nph.15688

[48] R. J. Norby, M. G. De Kauwe, T. F. Domingues, R. A. Duursma, D. S. Ellsworth, D. S. Goll, D. M. Lapola, K. A. Luus, A. R. MacKenzie, B. E. Medlyn, R. Pavlick, A. Rammig, B. Smith, R. Thomas, K. Thonicke, A. P. Walker, X. Yang, S. Zaehle, Model–data synthesis for the next generation of forest free-air CO_2_ enrichment (FACE) experiments. New Phytol. 209, 17–28 (2016). 26249015 10.1111/nph.13593

[49] A. P. Walker, P. J. Hanson, M. G. De Kauwe, B. E. Medlyn, S. Zaehle, S. Asao, M. Dietze, T. Hickler, C. Huntingford, C. M. Iversen, A. Jain, M. Lomas, Y. Luo, H. McCarthy, W. J. Parton, I. C. Prentice, P. E. Thornton, S. Wang, Y.-P. Wang, D. Warlind, E. Weng, J. M. Warren, F. I. Woodward, R. Oren, R. J. Norby, Comprehensive ecosystem model-data synthesis using multiple data sets at two temperate forest free-air CO_2_ enrichment experiments: Model performance at ambient CO_2_ concentration. J. Geophys. Res. Biogeo. 119, 937–964 (2014).

[50] M. G. De Kauwe, B. E. Medlyn, S. Zaehle, A. P. Walker, M. C. Dietze, Y. Wang, Y. Luo, A. K. Jain, B. El-Masri, T. Hickler, D. Wårlind, E. Weng, W. J. Parton, P. E. Thornton, S. Wang, I. C. Prentice, S. Asao, B. Smith, H. R. McCarthy, C. M. Iversen, P. J. Hanson, J. M. Warren, R. Oren, R. J. Norby, Where does the carbon go? A model-data intercomparison of vegetation carbon allocation and turnover processes at two temperate forest free-air CO_2_ enrichment sites. New Phytol. 203, 883–899 (2014). 24844873 10.1111/nph.12847 PMC4260117

[51] S. Zaehle, B. E. Medlyn, M. G. De Kauwe, A. P. Walker, M. C. Dietze, T. Hickler, Y. Luo, Y. Wang, B. El-Masri, P. Thornton, A. Jain, S. Wang, D. Warlind, E. Weng, W. Parton, C. M. Iversen, A. Gallet-Budynek, H. McCarthy, A. Finzi, P. J. Hanson, I. C. Prentice, R. Oren, R. J. Norby, Evaluation of 11 terrestrial carbon–nitrogen cycle models against observations from two temperate Free-Air CO_2_ Enrichment studies. New Phytol. 202, 803–822 (2014). 24467623 10.1111/nph.12697 PMC4288990

[52] A. Rogers, B. E. Medlyn, J. S. Dukes, G. Bonan, S. Caemmerer, M. C. Dietze, J. Kattge, A. D. B. Leakey, L. M. Mercado, Ü. Niinemets, I. C. Prentice, S. P. Serbin, S. Sitch, D. A. Way, S. Zaehle, A roadmap for improving the representation of photosynthesis in Earth system models. New Phytol. 213, 22–42 (2017). 27891647 10.1111/nph.14283

[53] D. S. Ellsworth, K. Y. Crous, M. G. De Kauwe, L. T. Verryckt, D. Goll, S. Zaehle, K. J. Bloomfield, P. Ciais, L. A. Cernusak, T. F. Domingues, M. E. Dusenge, S. Garcia, R. Guerrieri, F. Y. Ishida, I. A. Janssens, T. Kenzo, T. Ichie, B. E. Medlyn, P. Meir, R. J. Norby, P. B. Reich, L. Rowland, L. S. Santiago, Y. Sun, J. Uddling, A. P. Walker, K. W. L. K. Weerasinghe, M. J. van de Weg, Y.-B. Zhang, J.-L. Zhang, I. J. Wright, Convergence in phosphorus constraints to photosynthesis in forests around the world. Nat. Commun. 13, 5005 (2022). 36008385 10.1038/s41467-022-32545-0 PMC9411118

[54] R. E. Mcmurtrie, H. N. Comins, The temporal response of forest ecosystems to doubled atmospheric CO_2_ concentration. Glob. Chang. Biol. 2, 49–57 (1996).

[55] S. Manzoni, P. Taylor, A. Richter, A. Porporato, G. I. Ågren, Environmental and stoichiometric controls on microbial carbon-use efficiency in soils. New Phytol. 196, 79–91 (2012). 22924405 10.1111/j.1469-8137.2012.04225.x

[56] R. E. McMurtrie, R. C. Dewar, B. E. Medlyn, M. P. Jeffreys, Effects of elevated CO_2_ on forest growth and carbon storage: A modelling analysis of the consequences of changes in litter quality/quantity and root exudation. Plant Soil 224, 135–152 (2000).

[57] X. He, L. Augusto, D. S. Goll, B. Ringeval, Y.-P. Wang, J. Helfenstein, Y. Huang, E. Hou, Global patterns and drivers of phosphorus pools in natural soils. Biogeosciences 20, 4147–4163 (2023).

[58] K. Allen, J. B. Fisher, R. P. Phillips, J. S. Powers, E. R. Brzostek, Modeling the carbon cost of plant nitrogen and phosphorus uptake across temperate and tropical forests. Front. For. Glob. Change 3, 00043 (2020).

[59] R. K. Braghiere, J. B. Fisher, K. Allen, E. Brzostek, M. Shi, X. Yang, D. M. Ricciuto, R. A. Fisher, Q. Zhu, R. P. Phillips, Modeling global carbon costs of plant nitrogen and phosphorus acquisition. J. Adv. Model. Earth Syst. 14, e2022MS003204 (2022). 10.1029/2022MS003204 PMC9539603 36245670

[60] S. Piao, S. Sitch, P. Ciais, P. Friedlingstein, P. Peylin, X. Wang, A. Ahlström, A. Anav, J. G. Canadell, N. Cong, C. Huntingford, M. Jung, S. Levis, P. E. Levy, J. Li, X. Lin, M. R. Lomas, M. Lu, Y. Luo, Y. Ma, R. B. Myneni, B. Poulter, Z. Sun, T. Wang, N. Viovy, S. Zaehle, N. Zeng, Evaluation of terrestrial carbon cycle models for their response to climate variability and to CO_2_ trends. Glob. Chang. Biol. 19, 2117–2132 (2013). 23504870 10.1111/gcb.12187

[61] N. Collier, F. M. Hoffman, D. M. Lawrence, G. Keppel-Aleks, C. D. Koven, W. J. Riley, M. Mu, J. T. Randerson, The International Land Model Benchmarking (ILAMB) System: Design, theory, and implementation. J. Adv. Model. Earth Syst. 10, 2731–2754 (2018).

[62] W. R. Wieder, A. S. Grandy, C. M. Kallenbach, P. G. Taylor, G. B. Bonan, Representing life in the Earth system with soil microbial functional traits in the MIMICS model. Geosci. Model Dev. 8, 1789–1808 (2015).

[63] G. D. Farquhar, S. von Caemmerer, J. A. Berry, A biochemical model of photosynthetic CO_2_ assimilation in leaves of C3 species. Planta 149, 78–90 (1980). 24306196 10.1007/BF00386231

[64] G. J. Collatz, J. T. Ball, C. Grivet, J. A. Berry, Physiological and environmental regulation of stomatal conductance, photosynthesis and transpiration: A model that includes a laminar boundary layer. Agric. For. Meteorol. 54, 107–136 (1991).

[65] O. Kull, B. Kruijt, Leaf photosynthetic light response: A mechanistic model for scaling photosynthesis to leaves and canopies. Funct. Ecol. 12, 767–777 (1998).

[66] S. Fatichi, S. Leuzinger, C. Körner, Moving beyond photosynthesis: From carbon source to sink-driven vegetation modeling. New Phytol. 201, 1086–1095 (2014). 24261587 10.1111/nph.12614

[67] A. P. Walker, A. P. Beckerman, L. Gu, J. Kattge, L. A. Cernusak, T. F. Domingues, J. C. Scales, G. Wohlfahrt, S. D. Wullschleger, F. I. Woodward, The relationship of leaf photosynthetic traits–V_cmax_ and J_max_–to leaf nitrogen, leaf phosphorus, and specific leaf area: A meta-analysis and modeling study. Ecol. Evol. 4, 3218–3235 (2014). 25473475 10.1002/ece3.1173 PMC4222209

[68] M. J. Hedley, J. W. B. Stewart, Method to measure microbial phosphate in soils. Soil Biol. Biochem. 14, 377–385 (1982).

[69] R. Wang, D. Goll, Y. Balkanski, D. Hauglustaine, O. Boucher, P. Ciais, I. Janssens, J. Penuelas, B. Guenet, J. Sardans, L. Bopp, N. Vuichard, F. Zhou, B. Li, S. Piao, S. Peng, Y. Huang, S. Tao, Global forest carbon uptake due to nitrogen and phosphorus deposition from 1850 to 2100. Glob. Chang. Biol. 23, 4854–4872 (2017). 28513916 10.1111/gcb.13766

[70] P. E. Thornton, N. E. Zimmermann, An improved canopy integration scheme for a land surface model with prognostic canopy structure. J. Clim. 20, 3902–3923 (2007).

[71] H. Hartmann, S. Trumbore, Understanding the roles of nonstructural carbohydrates in forest trees—From what we can measure to what we want to know. New Phytol. 211, 386–403 (2016). 27061438 10.1111/nph.13955

[72] Y. Sun, D. S. Goll, J. Chang, P. Ciais, B. Guenet, J. Helfenstein, Y. Huang, R. Lauerwald, F. Maignan, V. Naipal, Y. Wang, H. Yang, H. Zhang, Global evaluation of the nutrient-enabled version of the land surface model ORCHIDEE-CNP v1.2 (r5986). Geosci. Model Dev. 14, 1987–2010 (2021).

[73] V. Haverd, B. Smith, L. P. Nieradzik, P. R. Briggs, A stand-alone tree demography and landscape structure module for Earth system models: Integration with inventory data from temperate and boreal forests. Biogeosciences 11, 4039–4055 (2014).

[74] V. Haverd, M. Cuntz, L. P. Nieradzik, I. N. Harman, Improved representations of coupled soil–canopy processes in the CABLE land surface model (Subversion revision 3432). Geosci. Model Dev. 9, 3111–3122 (2016).

[75] B. Smith, I. C. Prentice, M. T. Sykes, Representation of vegetation dynamics in the modelling of terrestrial ecosystems: Comparing two contrasting approaches within European climate space. Glob. Ecol. Biogeogr. 10, 621–637 (2001).

[76] D. Ricciuto, K. Sargsyan, P. Thornton, The impact of parametric uncertainties on biogeochemistry in the E3SM land model. J. Adv. Model. Earth Syst. 10, 297–319 (2018).

[77] K. Oleson, D. Lawrence, G. Bonan, B. Drewniak, M. Huang, C. Koven, S. Levis, F. Li, W. Riley, Z. Subin, S. Swenson, P. Thornton, A. Bozbiyik, R. Fisher, C. Heald, E. Kluzek, J.-F. Lamarque, P. Lawrence, L. Leung, W. Lipscomb, S. Muszala, D. Ricciuto, W. Sacks, Y. Sun, J. Tang, Z.-L. Yang, *Technical description of version 4.5 of the Community Land Model (CLM)* (UCAR/NCAR, 2013); 10.5065/D6RR1W7M.

[78] X. Yang, W. M. Post, Phosphorus transformations as a function of pedogenesis: A synthesis of soil phosphorus data using Hedley fractionation method. Biogeosciences 8, 2907–2916 (2011).

[79] A. Haxeltine, I. C. Prentice, BIOME3: An equilibrium terrestrial biosphere model based on ecophysiological constraints, resource availability, and competition among plant functional types. Global Biogeochem. Cycles 10, 693–709 (1996).

[80] T. Hickler, B. Smith, M. T. Sykes, M. B. Davis, S. Sugita, K. Walker, Using a generalized vegetation model to simulate vegetation dynamics in northeastern USA. Ecology 85, 519–530 (2004).

[81] S. Zaehle, A. D. Friend, Carbon and nitrogen cycle dynamics in the O-CN land surface model: 1. Model description, site-scale evaluation, and sensitivity to parameter estimates. Global Biogeochem. Cycles 24, GB1005 (2010).

[82] A. D. Friend, A. K. Stevens, R. G. Knox, M. G. R. Cannell, A process-based, terrestrial biosphere model of ecosystem dynamics (Hybrid v3.0). Ecol. Model. 95, 249–287 (1997).

[83] V. S. Pathare, K. Y. Crous, J. Cooke, D. Creek, O. Ghannoum, D. S. Ellsworth, Water availability affects seasonal CO_2_-induced photosynthetic enhancement in herbaceous species in a periodically dry woodland. Glob. Chang. Biol. 23, 5164–5178 (2017). 28691268 10.1111/gcb.13778

[84] S. J. Cork, I. D. Hume, T. J. Dawson, Digestion and metabolism of a natural foliar diet (Eucalyptus punctata) by an arboreal marsupial, the koala (Phascolarctos cinereus). J. Comp. Physiol. B 153, 181–190 (1983).

[85] J. E. Drake, C. A. Macdonald, M. G. Tjoelker, P. B. Reich, B. K. Singh, I. C. Anderson, D. S. Ellsworth, Three years of soil respiration in a mature eucalypt woodland exposed to atmospheric CO_2_ enrichment. Biogeochemistry 139, 85–101 (2018).

[86] B. E. Medlyn, R. A. Duursma, D. Eamus, D. S. Ellsworth, I. C. Prentice, C. V. M. Barton, K. Y. Crous, P. De Angelis, M. Freeman, L. Wingate, Reconciling the optimal and empirical approaches to modelling stomatal conductance. Glob. Chang. Biol. 17, 2134–2144 (2011).

[87] Y. P. Wang, R. M. Law, B. Pak, A global model of carbon, nitrogen and phosphorus cycles for the terrestrial biosphere. Biogeosciences 7, 2261–2282 (2010).

[88] P. E. Thornton, B. E. Law, H. L. Gholz, K. L. Clark, E. Falge, D. S. Ellsworth, A. H. Goldstein, R. K. Monson, D. Hollinger, M. Falk, J. Chen, J. P. Sparks, Modeling and measuring the effects of disturbance history and climate on carbon and water budgets in evergreen needleleaf forests. Agric. For. Meteorol. 113, 185–222 (2002).

