# Supplementary material for: Carbon-phosphorus cycle models overestimate CO2 enrichment response in a mature Eucalyptus forest
Source: Sci Adv. 2024 Jul 3;10(27):eadl5822. doi: 10.1126/sciadv.adl5822 (PMC11221523; doi:10.1126/sciadv.adl5822)
Supplement: Supplementary file 1 — Supplementary Text Figs. S1 to S17 Tables S1 to S19 References [file sciadv.adl5822_sm.pdf]

Science Advances
AAAS

Supplementary Materials for

Carbon-phosphorus cycle models overestimate CO_2 enrichment response in a mature *Eucalyptus* forest

Mingkai Jiang *et al.*

Corresponding author: Mingkai Jiang, jiangmingkai@zju.edu.cn

Sci. Adv. **10**, eadl5822 (2024)
DOI: 10.1126/sciadv.adl5822

This PDF file includes:

Supplementary Text
Figs. S1 to S17
Tables S1 to S19
References

Supplementary Text

Supplementary Information 1: Model Description

This model-data model intercomparison includes the following models (in alphabetical order; Table S1): the Community Atmosphere Biosphere Land Emulator with the ability to couple with the Populations-Order-Physiology module (1.1. CABLE-POP; (*10*)), the Energy Exascale Earth System Model (E3SM) land model (1.2. ELM; (*20*)); the Generic Decomposition And Yield model (1.3. GDAY; (*28*)); the Lund-Potsdam-Jena General Ecosystem Simulator (1.4. LPJ-GUESS; (*29*)); the Organizing Carbon and Hydrology in Dynamic Ecosystems (1.5. ORCHIDEE; (*11, 72*)), which is also coupled to a microbial-explicit sub-module (1.5.2. ORCHIDEE-MIC; (*30*)); and the Quantifying the effects of interacting nutrient cycles on terrestrial biosphere dynamics and their climate feedbacks (1.6. QUINCY; (*13*)), which is coupled to the Jena Soil Model (JSM) that has explicit microbial processes (1.6.2. QUINCY-JSM; (*19*)). All models included in this study has explicit biogeochemical representation of carbon (C), nitrogen (N) and phosphorus (P) processes. Below we provide a detailed description of the model structure and their key assumptions.

1.1. CABLE-POP

CABLE-POP is a land surface model with a vegetation demography module (POP) that is capable of simulating the biophysical, biogeochemical and vegetation demographic processes at the landscape scale ((*10, 73, 74*); Figure S10). The model has four vegetation (i.e. leaf, stem, fineroot, labile), three litter (metabolic, structural, coarse woody debris) and three soil organic matter pools (microbial, slow, passive). There is one soil inorganic N pool and three soil inorganic P pools of different bioavailability (labile, sorbed, and occluded). CABLE-POP implements the Farquhar formulation to simulate leaf-level photosynthesis (*63*). Nutrient limitation on photosynthesis is realized via a relationship between leaf N concentration and Rubisco-limited photosynthetic rate (i.e. V_{cmax}), and is further modulated by a PFT-specific leaf P/N ratio for P limitation (*73*). Additionally, the model implements the coordination hypothesis to represent the co-limitation of Rubisco- and electron transport-limited photosynthesis ((*10*); Table S2). CABLE-POP assumes fixed C allocation to leaf, wood and fineroot. Plant tissue nutrient retranslocation is calculated based on fixed coefficients. Growth is downregulated through increased autotrophic respiration if plant is nutrient stressed. Plant nutrient uptake is a function of the relative plant nutrient demand and the availability of inorganic N and inorganic labile P pool. In addition to biological P mineralization, the model also simulates a biochemical P mineralization flux,

following (*8*). Soil organic matter decomposition is controlled by maximum decay coefficients and soil texture, temperature, moisture and nutrient availability (Table S3; (*18*)).

Vegetation demography is realized via the integration of the POP module. The POP module simulates the allometric growth of cohorts of trees that compete for light and soil resources within a patch (*10*). Patch parameterizations of tree growth, allometry, recruitment and mortality are broadly based on the approach of the LPJ-GUESS model (*75*). Annual grid-scale stem biomass increment are calculated in the land surface model, and then partitioned among cohorts and patches in proportion to the current net primary production of the given cohort through the coupling of the POP module (*10*).

In this MIP, CABLE-POP simulation assumes broadleaf evergreen trees as the only vegetation type in the landscape.

1.2. ELM-v1

ELM (v1) is a land surface model that is capable of simulating C, water, N and P cycle processes at the stand-to-globe scale (*9*). The model includes a prognostic P cycle and CNP interactions, non-structural vegetation storage pools of C, N and P, and vertically resolved soil biogeochemistry (Figure S11; (*9, 20, 76*)). Photosynthesis in ELM is based on the Farquhar formulation for C3 plants (*63, 77*). Nutrient limitation on photosynthesis is realized via leaf N content dependence of photosynthetic capacity (*70*). Plant P uptake is a function of plant P demand and soil labile P availability (*18*). Plant nutrient retranslocation is calculated based on fixed coefficients (*18*). Plant C allocation is resource dependent (i.e. dynamic allocation based on light, nutrient availability, and water stress; Table S2). The relative limitation of N and P on growth follows the Liebig's law of minimum (*9*). To represent soil inorganic P of different bioavailability as characterized by the Hedley fractionation method (*78*), the model implements a five-pool structure for soil inorganic P pools (*8*). Biochemical P mineralization follows the (*8*) implementation. Soil organic matter decomposition is controlled by maximum decay coefficients and soil temperature, moisture and nutrient availability (*18*).

1.3. GDAY

GDAY is an ecosystem model developed for simulating C, water, N and P cycle at the stand-scale (*18*, *27*, *28*). The model includes four vegetation and three organic soil compartments,

each with its respective C, N and P pools (Figure S12). The model includes one inorganic soil nitrogen pool and five inorganic soil phosphorus pools (parent material, labile, sorbed, strongly sorbed, and occluded). The model implements the Farquhar formular for photosynthesis (*63*). Nutrient limitation on photosynthesis is realized via leaf N-dependence of V_{cmax} and leaf P-dependence of J_{max} (*67*). Carbon allocation in the model follows a functional allometric relationship and is also resource dependent (i.e. increased belowground allocation if nutrient stressed; Table S2). The relative limitation of N and P on growth follows the Liebig's law of minimum. Plant nutrient retranslocation is calculated based on fixed coefficients. Plant nutrient uptake depends on soil nutrient availability, plant nutrient demand and root biomass. Soil organic matter decomposition is controlled by maximum decay coefficients and soil temperature, moisture and nutrient availability. Additionally, GDAY implements an exudation scheme to allow newly assimilated plant C to be allocated directly into the soil (Figure S12), which in turn can stimulate soil organic matter decomposition and therefore the turnover of soil nutrients (more details below; (*28*)). Biochemical P mineralization follows the (*8*) implementation.

In GDAY, plant rhizosphere C allocation via root exudate that enters the active soil organic matter pool is represented as:

$$C_{rhizo} = NPP * a_r * a_{rhizo} * f_{cue,rhizo}$$

where *NPP* represents net primary production, a_r represents root C allocation coefficient, a_{rhizo} represents the fraction of root C allocation that is partitioned to rhizosphere deposition via root exudates, and $f_{cue,rhizo}$ is the microbial use efficiency parameter (0.3). The increased active soil organic matter nutrient demand is associated with the revised decomposition rate of the slow soil organic matter pool, expressed as:

$$k_{slow,new} = k_{slow} * (1 + k_m) \frac{C_{rhizo}}{C_{rhizo} + k_m}$$

Where k_{slow} is the original decomposition rate of the slow soil organic matter pool, and k_m is a sensitivity parameter. The decomposition rate of the slow soil organic matter pool affects the amount of nutrient released from the slow organic matter pool, expressed as:

$$N_{Rs} = k_{slow,new} * C_{slow}[n_s (1 - \Omega_{ss}) - n_p \Omega_{ps}]$$

Where C_{slow} is the slow soil organic matter pool, and Ω_{ss} and Ω_{ps} represent the proportion of C released through the decomposition of the slow and passive soil organic matter pools that subsequently enters the slow soil organic matter pool, respectively.

In this MIP, we included two set of simulations from GDAY, namely the model with CNP biogeochemistry (GDAYP), and the model with CN biogeochemistry (GDAYN). The difference between the two versions of the model is the inclusion of the phosphorus cycle processes, with all parameters model setting and forcing data being the same. Plant nutrient stress effect on photosynthesis follows a leaf N relationship developed by (*67*). The comparison of the two model simulation results therefore identifies the apparent effect of the phosphorus cycle.

1.4. LPJ-GUESS

LPJ-GUESS is a process-based dynamic global vegetation model (DGVM) capable of simulating stand-to-globe vegetation biophysics, biogeochemistry and demography (*29, 75*). The model has four vegetation carbon pools (foliage, sapwood, heartwood, fineroot), a surface layer consists of metabolic, structural, active and humus organic pools and multiple soil layers each consist of metabolic, structural, active, slow and passive soil organic pools (Figure S13). The model also includes non-structural C and labile N and P pools in the vegetation compartment (Table S2). Photosynthesis in the model assumes the (*64, 79*) formulations. Nutrient limitation on photosynthesis follows a relationship developed based on tropical and subtropical plants for the fully coupled CNP version (*53*), and for the CN version (*79*). Allocation follows functional allometric relationship and is also resource dependent (Table S2). Plant nutrient retranslocation is bound by a maximum resorption coefficient and is further determined by plant labile nutrient pool. Plant nutrient uptake is a function of plant nutrient demand and the size of the inorganic N and labile P pools. Soil organic matter decomposition is controlled by maximum decay coefficients and soil texture, temperature, moisture and nutrient availability.

Vegetation dynamics in LPJ-GUESS is an emerging feature of plant growth and competition for light, space and soil resources among woody plant individuals and a herbaceous understorey in each of a number of replicate patches for each simulated site or grid cell (*73*). Individuals for woody PFTs are identical within a cohort (age/size class) and patch. Population dynamic (establishment and mortality) are represented as stochastic processes, influenced by current resource status, demography and the life-history characteristics of each PFT (*29, 80*).

There are two sets of model simulations based on LPJ-GUESS, namely LPJGP and LPJGN. The LPJGP set of simulation is based on the CNP version of the model, whereas LPJGN is the CN simulation. Overstorey PFT is evergreen broadleaf in the model.

1.5. ORCHIDEE

There are two versions of ORCHIDEE included in this study, namely the CNP version (i.e. ORCHIDEE-CNP, v1.2) and its coupling to a microbial module (i.e. ORCHIDEE coupled with an explicit microbial module, ORCHIDEE-MIC). Below we describe the land surface model first (section 1.5.1), followed by the description of the microbial module (section 1.5.2).

1.5.1. ORCHIDEE-CNP

ORCHIDEE-CNP (v1.2) simulates the biophysical and biogeochemical dynamics at the stand-to-globe scale (Figure S14) (*15, 72*). The model includes non-structural vegetation storage pools of C, N and P. Plant labile P pool is dynamic with short- and long-term storage to buffer the imbalance between P supply and demand (Table S5). Photosynthesis in the model follows the Farquhar implementation (*63*), and is downregulated via the relationships between leaf nutrient concentration (N and P) and photosynthetic capacity parameters (V_{cmax} and J_{max}), following ref. (*53*). Plant C allocation is based on a functional allometric relationship that is resource dependent (Table S2). The model has a detailed representation of root uptake of dissolved N and P, in that N uptake is a function of plant N demand, root biomass, root uptake capacity and the inorganic N availability, and P uptake is a function of plant P demand, root biomass, root uptake capacity, dissolved labile P pool, and soil diffusivity ((*72*); Table S5). Plant nutrient retranslocation is calculated based on fixed coefficients. Soil organic matter decomposition is controlled by maximum decay coefficients and soil texture, temperature, moisture and nutrient availability (Table S3). The model implements a simple soil inorganic P representation with two pools (*15*). Soil P occlusion rate is a fixed fraction of the labile sorbed P pool. Soil P biochemical mineralization is a dynamic function of leaf N:P imbalance and substrate availability (Table S5).

1.5.2. ORCHIDEE-MIC

The microbial module for ORCHIDEE has vertically resolved soil biogeochemistry (Figure S15; Table S2; (*30*)). The model (i.e. OCHDX) implemented a MIMICS-type microbial scheme (*62*) that splits soil microbes into two different strategy groups that compete for resources with varying carbon use efficiency dynamics. It assumes non-linear decomposition rates of organic matter, which are regulated by the microbial biomass. The microbial growth is limited by the

availability of C, N, and P. Microbes can adjust their CUE in response to changes of available C or nutrients.

1.6.QUINCY

There are two versions of QUINCY included in this study, namely the land surface model with its default soil structure and processes (QUINCY; section 1.6.1), and its coupling to the Jena Soil Model that provides more explicit and advanced representation of soil biological and biogeochemical processes (i.e. QUINCY-JSM; section 1.6.2).

1.6.1. QUINCY

QUINCY is a terrestrial ecosystem model with fully coupled and seamless integration of the C, N and P cycles and their interaction with the terrestrial water and energy balance (*13*). The model includes three fast structural tissue pools (leaves, fine roots and fruits), one respiring non-structural pool (labile), one seasonal, non-respiring and non-structural storage pool (reserve), and three longer-lived structural tissue pools (sapwood, heartwood, and coarse roots; Figure S16). The model calculates photosynthesis based on a two-leaf approximation, with leaf N taken into account ((*65, 81, 82*); Table S4). Photosynthesis is downregulated by sink limitation when nutrient or water availability or low temperature limit growth and cause accumulation of photosynthates in the labile pool (*66, 71*). Plant growth is modelled as the balance of source processes (photosynthesis, nutrient uptake) and the capacity of the plant to create sinks (production of new biomass tissue, respiration, and storage). Effect of P limitation is realized via leaf N:P ratio effect on photosynthesis and sink limitation of plant labile pool (Table S2). Plant retranslocation is modelled based on a fixed coefficient approach. The response of plant nutrient uptake to plant demand is modelled as a lagged response (of a few days) to balance short-term fluctuations in photosynthesis and soil nutrient availability and to represent memory effects in the plant's control of its nutrient uptake (*13, 81*). The model has an explicit representation of vertical soil processes to separate litter and soil organic matter dynamics. Plant nutrient uptake is assumed linearly dependent on fine root biomass density for each soil layer and follows a Michaelis-Mention parameterization to simulation the effects of soil soluble NH_4, NO_3, and PO_4 concentrations (*13*, *79*). The model has five soil inorganic P pools. Decomposition of soil organic matter is a function of maximum decay coefficients and soil texture, temperature, moisture and nutrient availability.

1.6.2. QUINCY-JSM

QUINCY is coupled with the Jena Soil Model (JSM) to explicitly simulate the non-linear dynamics of the microbial community and their influence on soil biogeochemistry and plant growth. JSM is a soil model that is microbially explicit, vertically resolved and integrated with the N and P cycles ((*19*); Figure S17). JSM includes the representation of enzyme allocation to different depolymerization sources based on the microbial adaptation approach as well as of nutrient acquisition competition based on the equilibrium chemistry approximation approach.

Supplementary Information 2: Simulation protocols

2.1. Overview

Overarching objectives of this multi-model intercomparison project:

1) Use models to assess existing modelling skills in capturing the observed elevated CO_2 responses at the nutrient-limited EucFACE (2012 – 2019),
2) Use models to evaluate existing modelling skills in simulating drought x CO_2 interaction at EucFACE (2012 – 2019),
3) Use models to predict forest response to elevated CO_2 and phosphorus fertilization at EucFACE (2020 – 2069).

Below is the modelling protocol description.

2.2. Current model list

The list of models involved in this intercomparison project is provided in Table S6, together with their 5-character abbreviations.

2.3. Forcing data

Meteorological forcing files can be obtained from the link below via the link (download only):

https://doi.org/10.6084/m9.figshare.25853455.v1

2.4. Model simulation description

1) Spin up models using a standard PFT (i.e. one that would represent the subtropical/temperate evergreen broadleaf *Eucalyptus* trees in your model) and the provided forcing data. We provide a 50-year meteorological forcing for model spin-up purpose (at both daily and half-hourly timestep, choose based on your need), and this file should be recycled until equilibrium is reached. The site should be modelled as a forest (with C_3 grass understorey if possible), site parameters can be used to adjust the model to the site conditions. A document detailing various site-level parameters is available. The spin-up, pre-industrial CO_2 (year 1750) should be set to 276.84 μmol mol^{-1} and the nitrogen deposition is 2.25 kg N hectare^{-1} year^{-1}. Atmospheric

phosphorus deposition is set at 0.093 kg P hectare^{-1} year^{-1} for spin-up, historic, observed, and future period. For N fixation, we ask models to use their own function to estimate rate of N fixation for the site, and report this variable as model output (as indicated in the output protocol). We would recommend using a minimal rate of N fixation, as explained in the parameter file.

2) Models should then be run for the historic period from pre-industrial to present-day, i.e. 1750-2011. Simulations should use the same PFT parameterization and the provided meteorological forcing for the historic period. We provide both daily and half-hourly timestep forcing files. Note that CO_2 and N deposition data vary at annual timestep, as in the provided files.

3) Four simulations should then be run covering the period of 2012-2019 (7-years), which we define as the observed period:

   a) A set of simulations that uses the observed variable meteorological forcing (OBS-VAR), which includes period of wet and dry years, to provide a site baseline response at:

      i) ambient CO_2 (AMB);

      ii) elevated CO_2 (ELE).

   b) A set of simulations that repeats the wet-year meteorological forcing to represent a wet response scenario (OBS-FIX), at:

      i) ambient CO_2 (AMB);

      ii) elevated CO_2 (ELE).

4) Twelve simulations should then be run covering the period of 2020 – 2069 (50-years into the future), to predict the likely long-term consequence of elevated CO_2 and phosphorus fertilization treatment:

   a) A set of simulations that repeats the observed 7-year meteorological forcing data into the future (PRD-VAR), at:

      i) Ambient CO_2 (AMB), with three levels of phosphorus fertilization treatments:

         (1) No additional phosphorus addition (NOP);

         (2) Moderate phosphorus addition at a rate of 1.5 g P m^{-2} yr^{-1} addition for the first three years of the simulation (MDP), with a turnover rate of 1-yr;

(3) High phosphorus addition at a rate of 3.0 g P m^{-2} yr^{-1} addition for the first three years of the simulation (HIP), with a turnover rate of 1-yr;

ii) Elevated CO_2 (ELE), with the same three levels of phosphorus fertilization rate:

(1) No additional phosphorus addition (NOP);

(2) Moderate phosphorus addition at a rate of 1.5 g P m^{-2} yr^{-1} addition for the first three years of the simulation (MDP), with a turnover rate of 1-yr;

(3) High phosphorus addition at a rate of 3.0 g P m^{-2} yr^{-1} addition for the first three years of the simulation (HIP), with a turnover rate of 1-yr;

b) A set of simulations that repeats the wet-year meteorological forcing to represent a wet response scenario (PRD-FIX), at:

i) Ambient CO_2 (AMB), with three levels of phosphorus intervention:

(1) No additional phosphorus addition (NOP);

(2) Moderate phosphorus addition at a rate of 1.5 g P m^{-2} yr^{-1} addition for the first three years of the simulation (MDP), with a turnover rate of 1-yr;

(3) High phosphorus addition at a rate of 3.0 g P m^{-2} yr^{-1} addition for the first three years of the simulation (HIP), with a turnover rate of 1-yr;

ii) Elevated CO_2 (ELE), with three levels of phosphorus intervention:

(1) No additional phosphorus addition (NOP);

(2) Moderate phosphorus addition at a rate of 1.5 g P m^{-2} yr^{-1} addition for the first three years of the simulation (MDP), with a turnover rate of 1-yr;

(3) High phosphorus addition at a rate of 3.0 g P m^{-2} yr^{-1} addition for the first three years of the simulation (HIP), with a turnover rate of 1-yr.

Ambient CO_2 concentration for the future period follows the current trajectory of CO_2 rise (i.e. +3 ppm yr^{-1}). Thus, by the end of the simulation (i.e. 2069) the ambient CO_2 treatment would have CO_2 concentration equivalent to current elevated CO_2 treatment at the site. Elevated CO_2 treatment for the first 10-year of the future period (i.e. 2020 – 2029) follows the ambient + 150 ppm treatment. Beyond year 2029, elevated CO_2 treatment is fixed at the 2029 level.

For all simulations, the meteorological forcing at a 30-minute or daily timestep is provided.

An overview table of the 16 observed-to-future simulations is provided in Table S7.

2.5. Outputs

A document has been provided outlining the expected organization of simulation output files. These should be provided as comma separated (CSV) files at either an hourly or daily time-step (or both if you can).

It is important that output files contain the headings exactly as outlined. Where an output is not generated by the model the column should be filled with "-9999.9".

Simulation output should be labelled:

EUC_MMMMM_SSS_XXX_YYY_ZZZ_T.csv

where

- EUC is the 3-character label for EucFACE;
- MMMMM is the 5-character model abbreviations, e.g. GDAYP or GDAYN;
- SSS is the 3-character simulation timeframe, either OBS = observed, or PRD = predicted;
- XXX is either VAR = 7-year variable meteorology data with real drought, or FIX = repeated wet-year meteorology;
- YYY is either AMB = ambient or ELE = elevated CO_2 treatment runs;
- ZZZ is the phosphorus treatment, either NOP = no P intervention, MDP = moderate P intervention, or HIP = high P intervention;
- T is either H or D, depending on whether the time-step is hourly (H) or daily (D).

Supplementary Information 3: EucFACE parameter list

3.1. Overview

This parameter list is provided based on data collected from EucFACE. It serves as a look-up table to parameterize and evaluate model simulations. Hence, included within are parameter variables as well as evaluation datasets. Unless otherwise stated, data provided below are initial values (year 2012) based on ambient CO_2 plots only.

3.2. General site description

A mature *Eucalyptus* woodland, located in western Sydney, Australia. Site elevation: 22 m above sea level. Six plots at EucFACE, with three subject to elevated CO_2 treatment (ambient +150 ppm). These plots are hexadecagons that are 25 m in diameter, with 32 vertical stand pipes for CO_2 release. Plot area = ~490 m^2. EucFACE longitude and latitude is: -33.61°, and 150.73°.

The dominant species is *Eucalyptus tereticornis* Sm., accounting for > 98% of the stem area within the plots. The understorey is a diverse mixture of C_3 and C_4 vegetation, but *Microlaena stipoides* – a C_3 perennial grass – dominates the understorey (~ accounting for 70% of the herbaceous biomass). More detailed site description can be obtained from ref. (*23*).

3.3. Major ecosystem carbon pools

A 4-year averaged (2013 – 2016), ecosystem-scale carbon budget is provided in Figure 1 in ref. (*23*). Some earlier data is provided in (*21*) and some other papers included in the paper folder. More specifically, a summary table for evaluation or initialization purpose is provided in Table S8.

3.4. Soil physical properties

Surface soil (upper 45 cm) for Clarenden sand: 80 ± 8% sand, 9 ± 5% silt, 11 ± 3% clay.

Table S9 is the soil texture and bulk density profile across depth at EucFACE. Note that the soil texture information is taken directly from (*17*). Soil bulk density data for the top 30 cm of the soil is taken from data measured in 2017 at the plot-level at EucFACE. Soil bulk density data for depth below top 30 cm of the soil is taken from (*17*), which is pre-treatment measurement.

3.5. Soil water properties

Field capacity was estimated from actual total storage estimates based on neutron probe measurements. This is high because the soil drains poorly/slowly, so more water is available to the plant. Notes: "effective" because it averages over the different soil layers (considering differences in texture, bulk density, wilting points estimated from release curves). A time-series soil volumetric water content dataset at different soil depths for both the ambient and elevated treatment plots is provided in evaluation dataset folder. Soil water properties are provided in Table S10.

3.6. Soil chemical properties

Table S11 summarizes the key soil chemical properties at EucFACE. For soils beyond the top 10 cm, we have limited data so these values are averaged across different dates and treatments to maximize sample size. Soil labile inorganic P pool and labile organic P pool were determined based on Hedley fractionation method, which refer to the "bioavailable" P pool that plants can take up. The temporally-dynamic soil N and P mineralization data result is available in (*34*).

3.7. Stoichiometry

An earlier publication is available that reports C:P ratios for overstorey leaf and sapwood (*40*). C:N ratio for understorey green leaf is available in (*83*). Soil depth is top 10 cm. Table S12 summarizes major plant and soil CNP ratios under ambient CO_2 treatment.

3.8. Plant nutrient retranslocation coefficients

An earlier publication is available based on partial data (*40*), and Table S13 provides a complete summary. Note that some values are assumed.

3.9. Canopy properties

A time-series canopy leaf area index is available in (*32*) and (*23*), and we also provide this time-series dataset in the evaluation dataset folder for model evaluation purpose. LAI is a representation of plant leaf area, which includes contribution from woody components (~0.8 m^2 m^{-2}). Leaf length is available from Ben Moore. Leaf lignin information is sourced from (*84*), based on *Eucalyptus punctata*. More details about leaf lifespan is available in (*32*).

Upper-canopy biochemical parameters (i.e. V_{cmax} and J_{max}) are estimated based on mature leaves across all measurement campaigns (i.e. Feb 2013 – Feb 2020, 433 A-C_i curves over 15 campaigns), normalized to 25 °C. The corresponding mature leaf mass per area, leaf nitrogen and phosphorus concentrations are: 191.6 ± 10.7 g m^{-2}, 1.63 ± 0.08 % and 0.071 ± 0.004 %, respectively. These values differed to the values provided below slightly, because they are only based on leaves used in the A-C_i curve analyses. For models that require leaf nutrient concentration and biochemical parameter relationships, we suggest using the existing relationship in the models and evaluate the model simulations based on the data provided below. Table S14 provides the canopy properties for model parameterization or evaluation purpose.

3.10. Understorey vegetation properties

The understorey is a diverse mixture of C_3 and C_4 vegetation, but *Microlaena stipoides* – a C_3 perennial grass – dominates the understorey (~ accounting for 70% of the herbaceous biomass). Understorey specific leaf area is estimated to be 130.79 ± 20.16 cm^2 g^{-1} for ambient plots (mean ± SD). Understorey aboveground biomass is estimated as 156 ± 20 g C m^{-2}.

3.11. Wood property

Wood density is the average value at the start of the experiment (basic density; mean ± SD, n =34), taken directly from (*17*). Sapwood turnover rate is estimated based on sapwood depth and average tree diameter. Stem diameter is measured at breast height of 1.3 m (DBH). Only trees with diameter > 10 cm at the start of the experiment (i.e. 2012) are included in Table S15. Also, all dead trees (considered dead in 2019) and shrubs are excluded in the table so that we have the same number of trees over time.

3.12. Root profile

Fine root lifespan: 1.5 year. Source is ref. (*38*).

3.13. NPP and allocation coefficient

Ecosystem carbon use efficiency (NPP/GPP) is 0.31 ± 0.03, which includes both overstorey and understorey vegetation. We can't differentiate overstorey and understorey roots, so we can't provide CUE for overstorey and understorey separately. Allocation coefficients are provided in Table S16. Note that allocation to leaf includes allocation to overstorey and understorey leaves. Allocation to root includes allocation to fine (< 2 mm in diameter) intermediate (2 – 3 mm) and coarse root (> 3 mm). Allocation flux to mycorrhizae was inferred based on data assimilation, as in Jiang *et al.* (*23*).

3.14. Soil respiration fluxes

We have a continuous and high-quality soil respiration dataset at EucFACE. The chamber-based soil respiration measurements were scaled-up to the EucFACE plot-level to obtain annual fluxes using the DAMM model (*85*). This dataset can be used as an independent evaluation for the model simulation results over the 2013 - 2015 period. Annual rate of soil respiration is provided in Table S17.

3.15. Nitrogen fixation

N fixation has not been measured on site, but we anticipate it would be low. There is a small number of N-fixing plants at the site, but they are low in abundance and sparsely distributed. Also, an initial survey of the lichen species shows that the species we have on site do not contain N-fixing symbionts. There could potentially be N fixation from biocrusts and free-living organisms, but these have not yet been investigated.

We would recommend using a minimal rate of N fixation.

Supplementary Information 4: Simulation output protocol

Please provide comma-delimited files (csv), at daily timestep if possible. For models that run at hourly timestep, we ask you to convert your output into daily timestep, but if possible, please also provide hourly output on the variables specified in the table below (Table A). Columns that you can't provide data for should be filled with -9999.9.

Definitions:

We need you to check your mass balance in your model output (i.e. C, N and/or P). Script for checking will be provided separately. If any variables are missing in the list below to achieve this in your model, please add them as new columns after the mandatory output data columns, and document them with proper definition and unit.

- NEP = GPP – Reco – Offsite C losses.
- N uptake = N in new wood growth + (new leaf N – N from internal pools) + N for new root production + (N deposition – N in throughfall – i.e., canopy uptake of atmospheric N.
- Mean Vapor Pressure Deficit in kPa.
- Wood and branch litterfall should include mortality of aboveground plants.
- Betah and Betad (soil moisture stress) is a factor between 0 and 1 that represents how much soil moisture stress has reduced GPP below its unstressed value.
- Plant available soil water (mm). i.e. soil water above wilting point and for the rooting depth (for models with an exponential root depth distribution this should be for the soil volume that contains >90-95% of roots).

Model Abbreviations:

Please use the dedicated 5-letter abbreviation for your model, as specified in the modelling protocol.

A. Hourly output -- Provide 16 files containing the following 38 columns in the specified order.

Output files should be called:

EUC_MMMMM_OBS_XXX_YYY_NOP_H.csv

where

- EUC is the 3-charater label for EucFACE;
- MMMMM is the 5-character model abbreviations, e.g. GDAYP or GDAYN;
- OBS is the 3-character simulation timeframe for 2013-2019 only;
- XXX is either VAR = 7-year variable meteorology data with real drought, or FIX = repeated wet-year meteorology;
- YYY is either AMB = ambient or ELE = elevated CO_2 treatment runs;
- NOP is the no P intervention treatment;
- H is hourly (H) model timestep.

Format of the hourly output file is available in Table S18.

B. Daily Output -- Provide 16 files containing the following 122 columns in the specified order.

Output files should be called:

EUC_MMMMM_SSS_XXX_YYY_ZZZ_D.csv

where

- EUC is the 3-charater label for EucFACE;
- MMMMM is the 5-character model abbreviations, e.g. GDAYP or GDAYN;
- SSS is the 3-character simulation timeframe, either OBS = observed, or PRD = predicted;
- XXX is either VAR = 7-year variable meteorology data with real drought, or FIX = repeated wet-year meteorology;
- YYY is either AMB = ambient or ELE = elevated CO_2 treatment runs;
- ZZZ is the phosphorus treatment, either NOP = no P intervention, MDP = moderate P intervention, or HIP = high P intervention;
- D is daily (D) model timestep.

Format of the daily output file is available in Table S19.

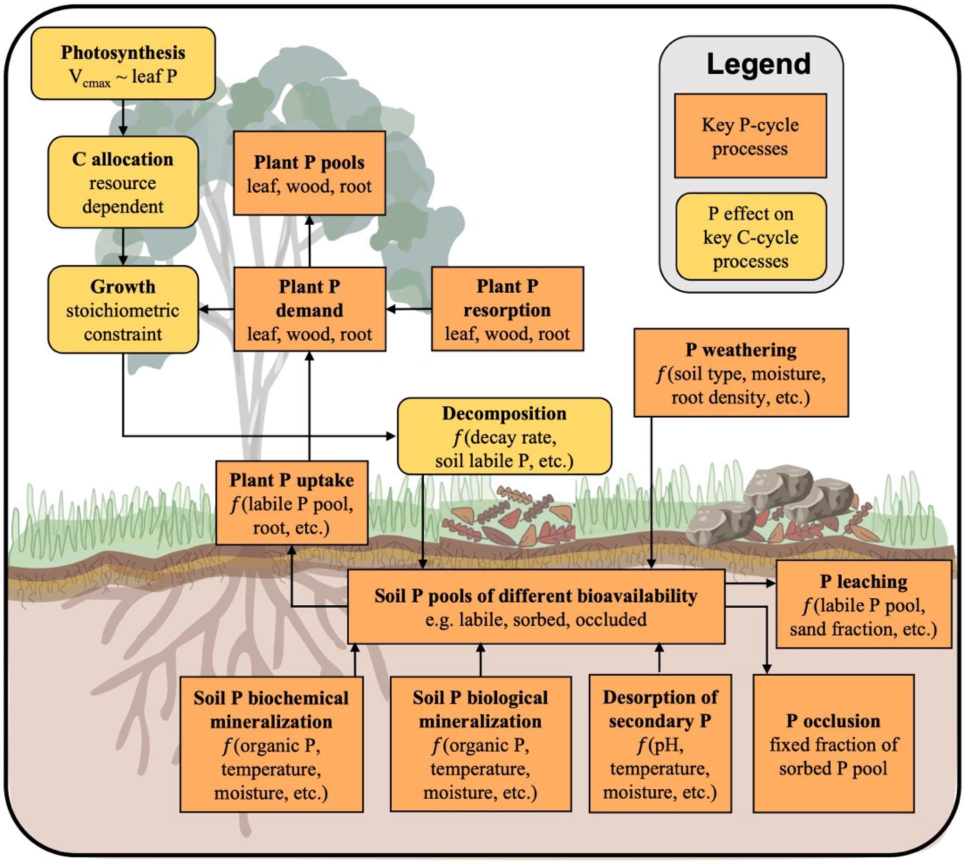

Fig. S1.

Schematic diagrams of the key phosphorus (P) cycle processes represented by P-enabled models, including common model features of the P-cycle processes, and P-cycle effect on carbon cycle processes. The details on how these processes vary across models are summarized in Table 1.

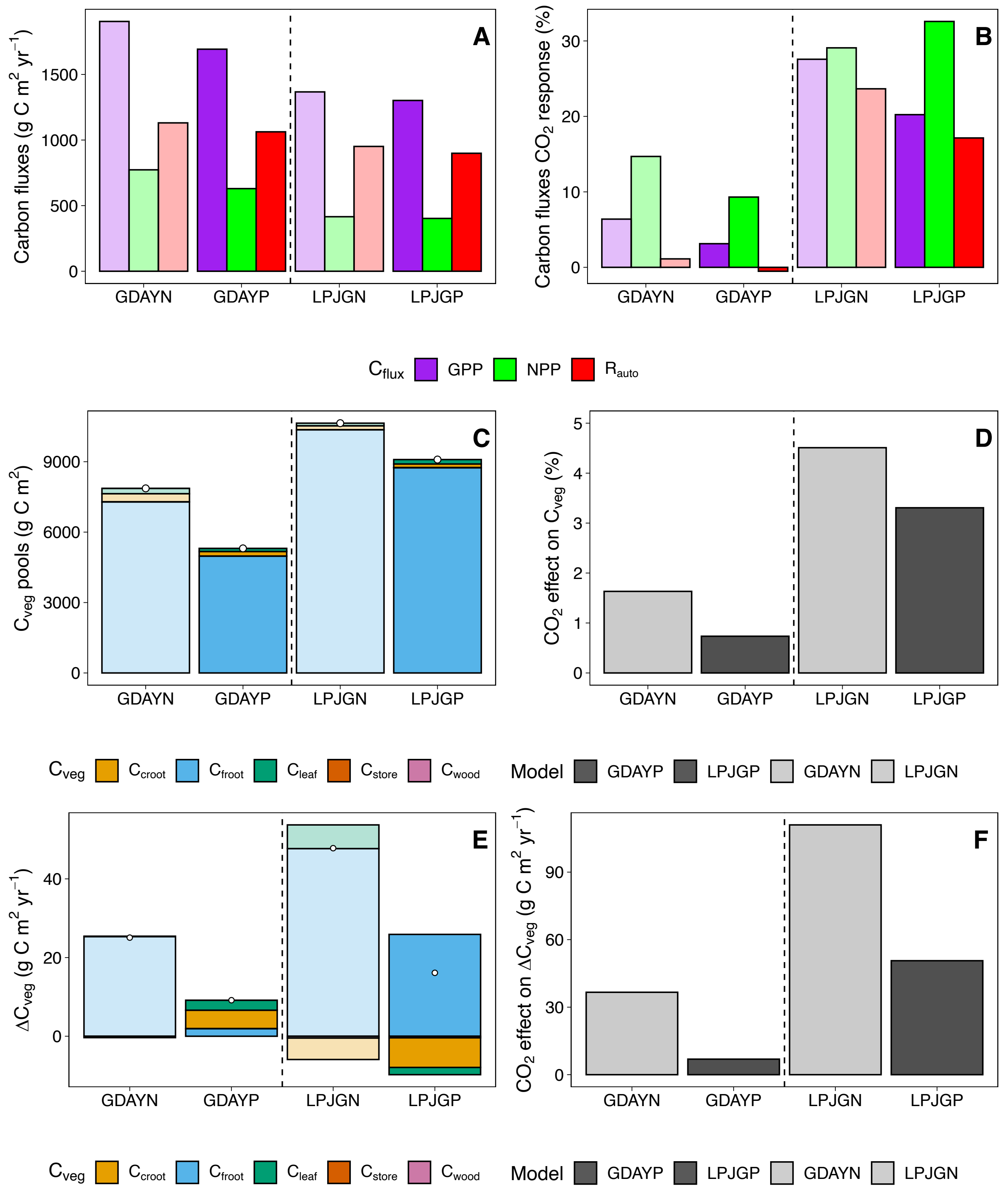

Fig. S2.

Comparison of the phosphorus (P) cycle effect in two models with and without P-cycle switched on. (A) major carbon fluxes, including gross primary production (GPP), net primary

production (NPP), and autotrophic respiration (R_{auto}); **(B)** the CO_2 responses for the major carbon fluxes reported in (a), calculated as (elevated – ambient) / ambient * 100; **(C)** major vegetation carbon pools (C_{veg}), as the sum of leaf, wood, fine root, coarse root, and plant storage C pools (C_{leaf}, C_{wood}, C_{froot}, C_{croot} and $C_{store,}$ respectively); **(D)** the CO_2 effect on C_{veg}; **(E)** annual incremental change in C_{veg} (ΔC_{veg}); and **(F)** the CO_2 effects on ΔC_{veg}. The two models included here are the stand-scale ecosystem model GDAY, and the dynamic global vegetation model LPJ-GUESS. GDAYN and LPJGN denote the CN models and GDAYP and LPJGP denote the CNP models, respectively. Results were averaged over the period of 2013-2018, under variable climate forcing. Error bars indicating the multi-year standard deviation.

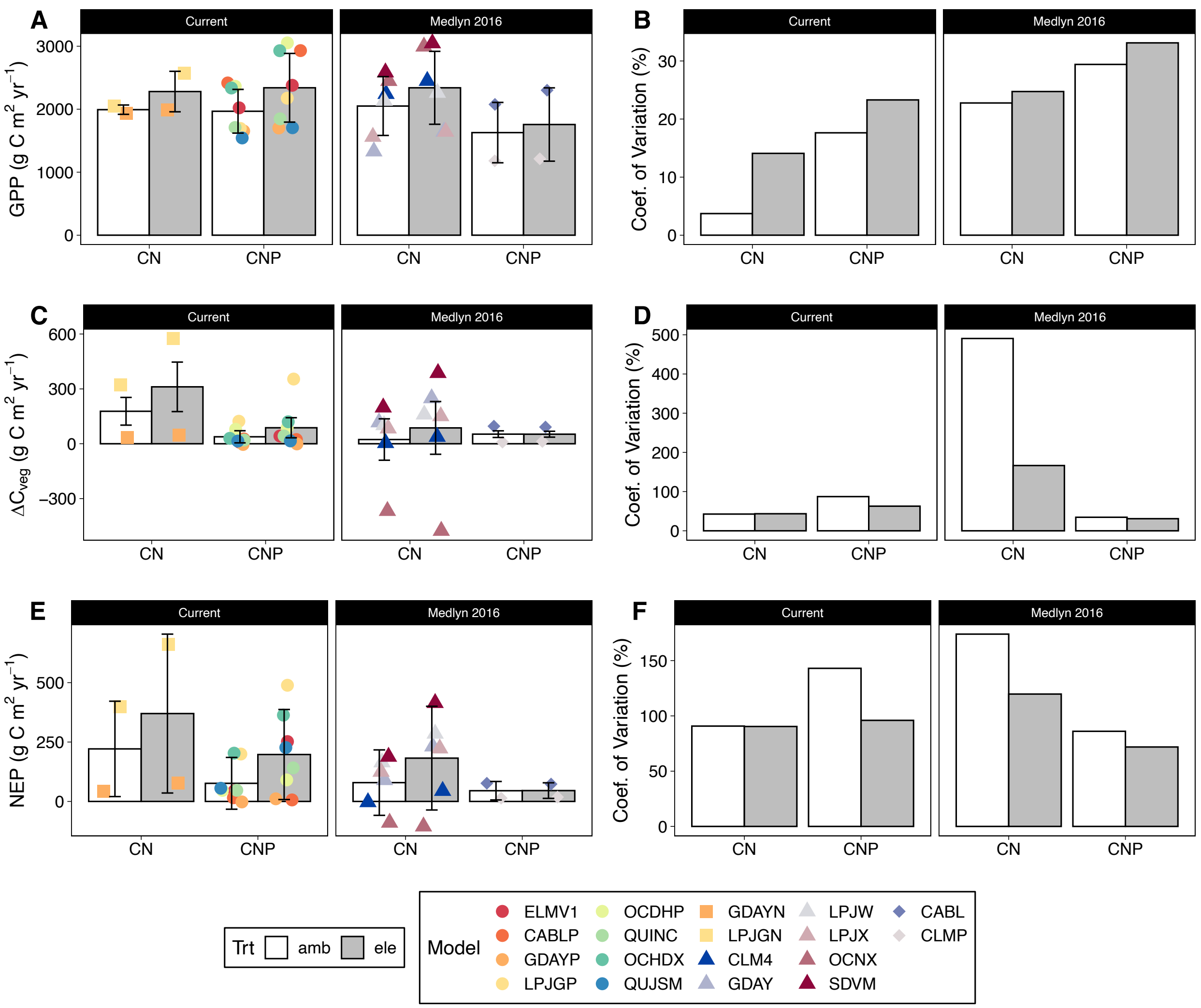

Fig. S3.

Comparison of the simulation spread among models included in this study and those reported previously based on *a priori* assumptions at EucFACE (*17*), for **(A, C and E)** gross primary production (GPP), annual incremental change in plant biomass (ΔC_{veg}), defined as the incremental change of carbon in plant biomass (including leaf, wood, and root only), and net ecosystem production (NEP), respectively, under ambient (white bar) and elevated CO_2 treatment (grey bar); and **(B, D and F)** the coefficient of variation among models for the respective model simulations for GPP, BP and NEP. The current work included two carbon-nitrogen (CN) coupled models and eight phosphorus-models (CNP), whereas ref. (*17*) included six CN models and two CNP models. Both sets of simulations were performed under a hypothetical, wet-year fixed climate, but with different daily variation. Results reported here were averaged over the 2013-2016

simulation period. Error bars indicate standard deviation of the multi-model means. Individual points indicate the multi-year averages for each model.

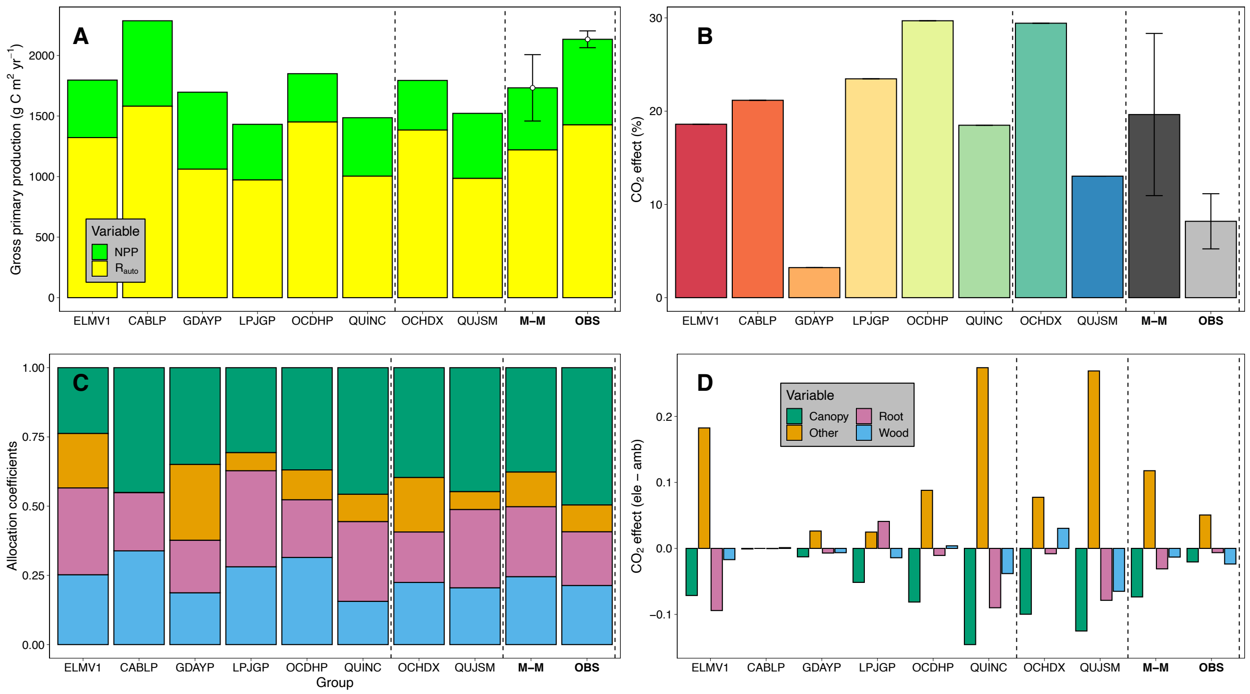

Fig. S4.

Data-model intercomparisons of the gross primary production (GPP) and carbon allocation coefficients (A and C) under ambient CO_2 treatment, and (B and D) the CO_2 effect. M-M indicates multi-model mean, whereas OBS indicates observation. Error bars indicate the standard deviations of the multi-model means and observations, respectively. GPP can be partitioned into net primary production (NPP) and autotrophic respiration (R_{auto}). Allocation coefficient is unitless.

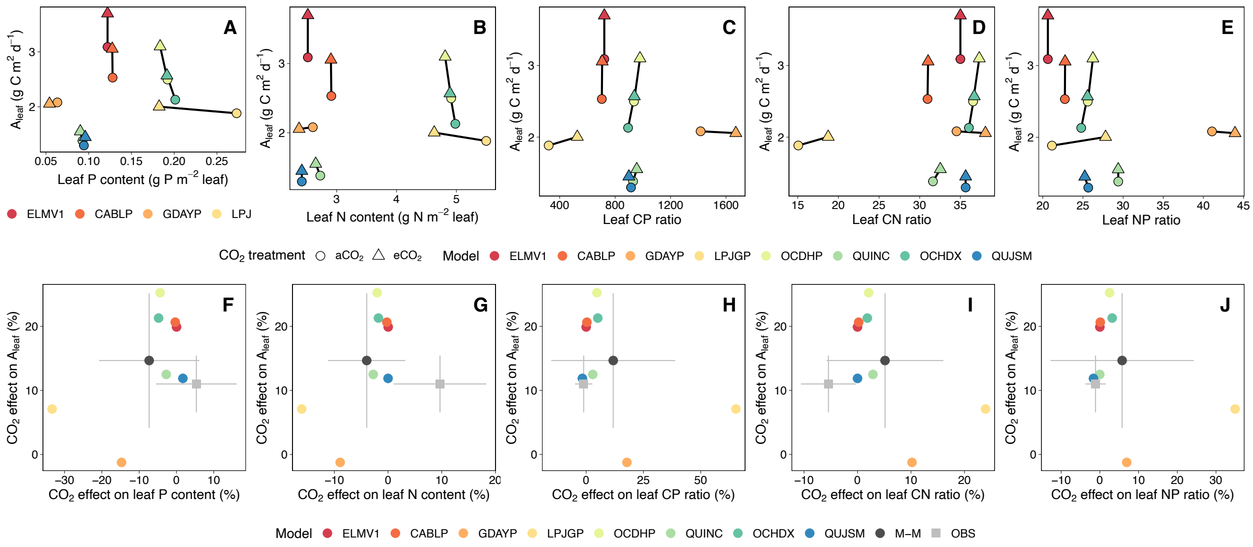

Fig. S5.

Data-model intercomparisons of the leaf nutrient relationship with leaf-level photosynthesis (A_{leaf}). (A-E) Leaf P content (g P m^{-2} leaf), leaf N content (g N m^{-2} leaf), leaf C:P ratio, leaf C:N ratio, and leaf N:P ratio relationships with A_{leaf} (g C m^{-2} leaf d^{-1}) under ambient and elevated CO_2 treatment, respectively. **(F-J)** The emergent constraint of the CO_2 effects (%) on leaf P content, leaf N content, leaf C:P ratio, leaf C:N ratio, and leaf N:P ratio and the CO_2 effect on A_{leaf} (%), respectively. M-M indicates multi-model mean, whereas OBS indicates observation. Error bars indicate the standard deviations of the multi-model means and observations, respectively.

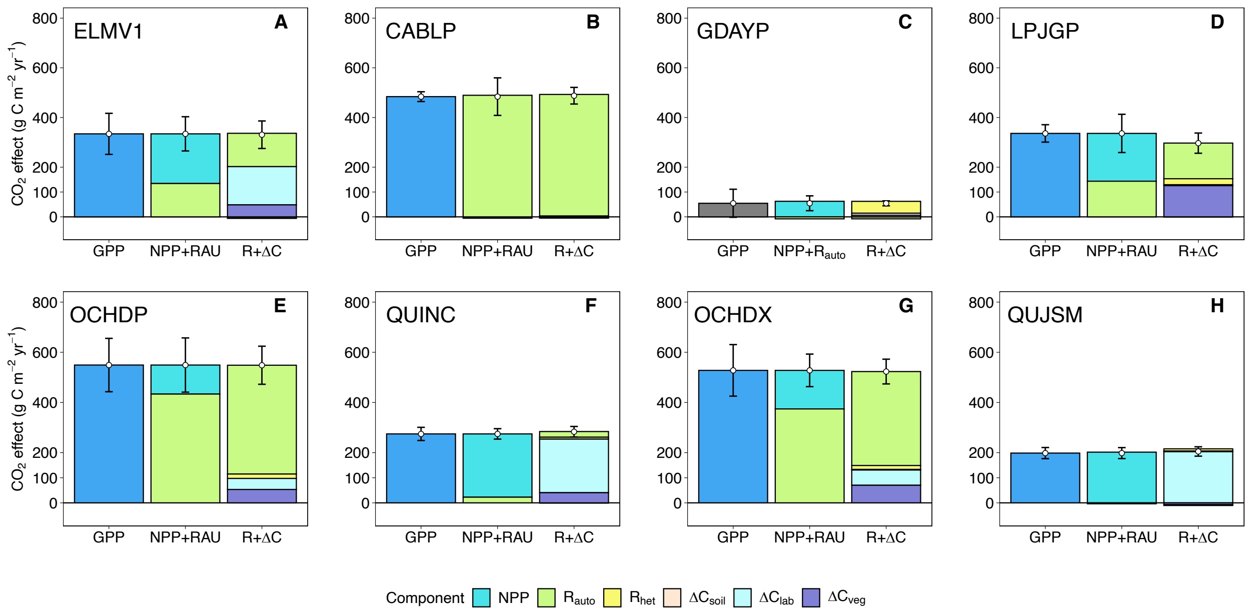

Fig. S6.

Individual model simulations of the CO_2 effect on major carbon (C) fluxes to track the fate of C along the plant-soil continuum in the ecosystem. (**A-H**): models ELMV1, CABLP, GDAYP, LPJGP, OCHDP, QUINC, OCHDX, QUJSM, respectively. The CO_2 effect on GPP can be explained by either the CO_2 effects on net primary production (NPP) and autotrophic respiration (R_{auto}), or the CO_2 effects on ecosystem respiration (R_{eco}) and annual incremental changes in C pools (ΔC). R_{eco} includes contributions from R_{auto} and heterotrophic respiration (R_{het}), whereas ΔC includes contributions from changes in structural and non-structural plant pools and soil C pool (ΔC_{veg}, ΔC_{store} and ΔC_{soil}, respectively). Results were averaged over 2013-16, under variable climate forcing. The error bars indicate standard deviation of the interannual variability.

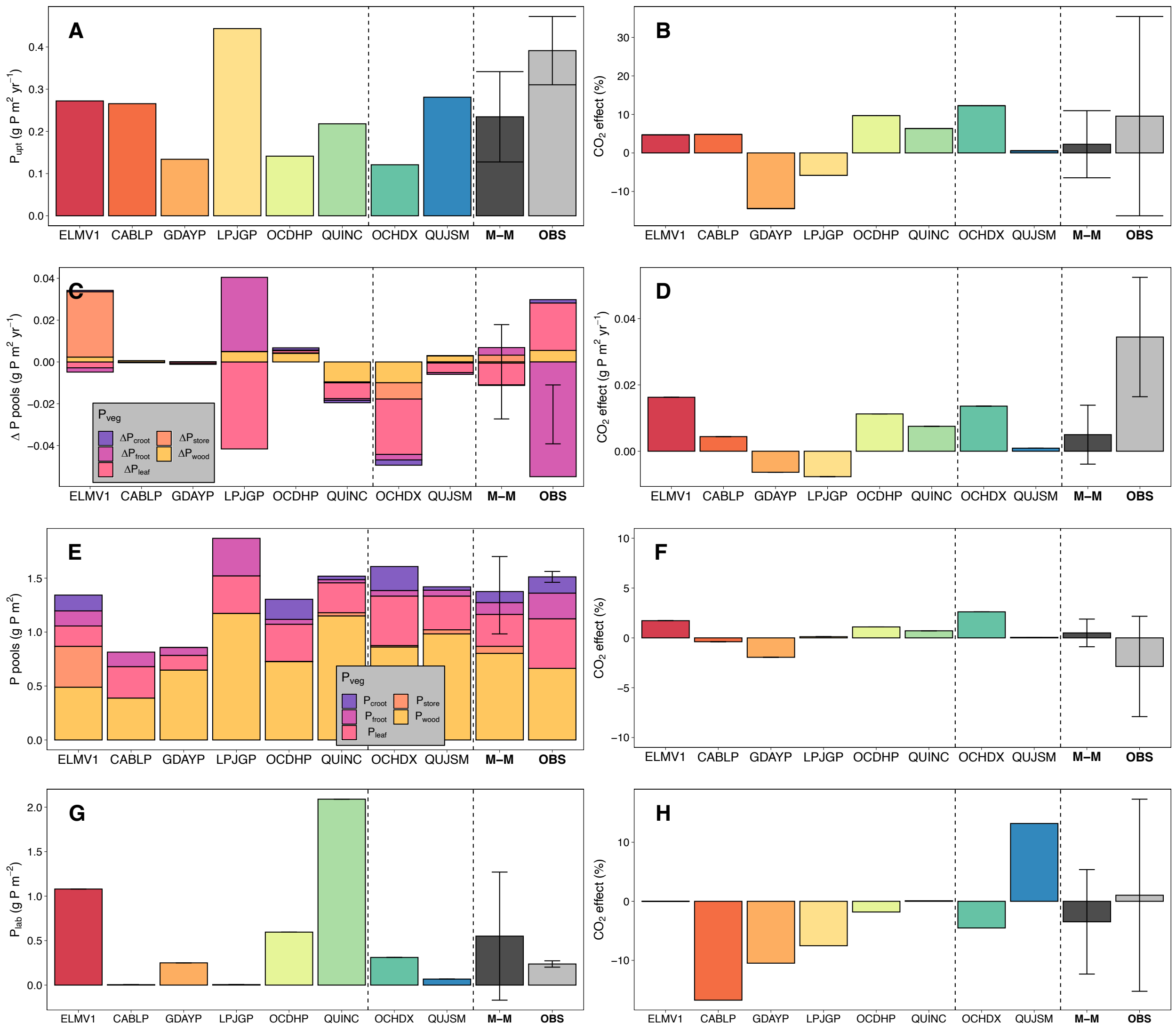

Fig. S7.

Data-model intercomparisons on key phosphorus-cycle variables under ambient CO_2 treatment and their responses to elevated CO_2. (A, C, E and G) plant P uptake flux, annual incremental changes in plant P pools, plant P pools and soil labile P pool (P_{upt}, ΔP_{veg}, P_{veg} and P_{lab} respectively) under ambient CO_2 treatment; **(B, D, F and H)** the CO_2 effect on P_{upt}, ΔP_{veg}, P_{veg} and P_{lab}. M-M indicates multi-model mean, whereas OBS indicates observation. The error bars indicate standard deviation of the interannual variability.

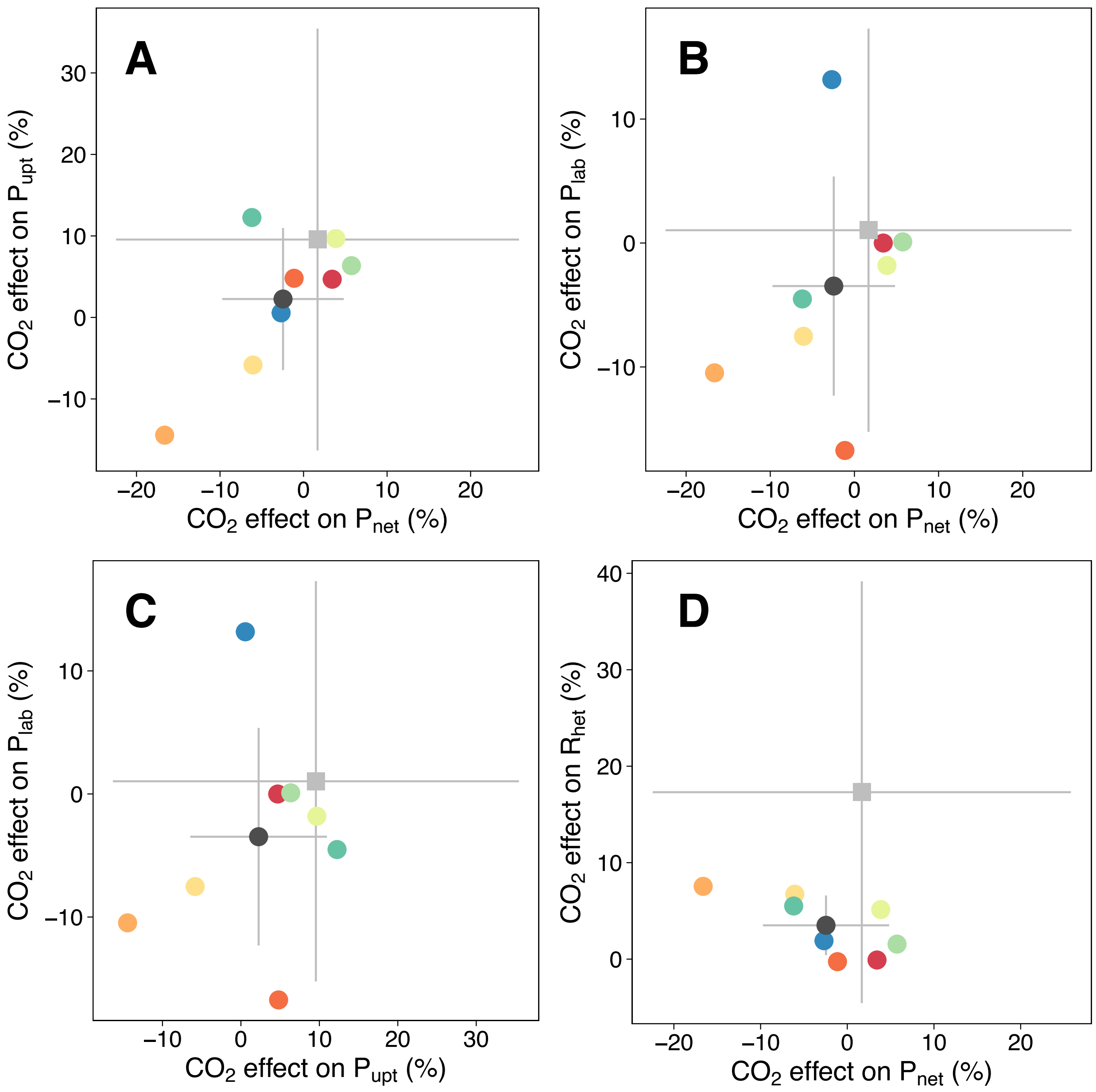

Fig. S8.

Emergent constraints on the CO_2 effects on key soil phosphorus-cycle processes. **(A)** The emergent constraint of CO_2 effects (%) on soil net P mineralization flux (P_{net}) and plant P uptake flux (P_{upt}). **(B)** The CO_2 effects (%) on soil net P mineralization flux (P_{net}) and soil labile P pool (P_{lab}). **(C)** The emergent constraint of the CO_2 effects (%) on plant P uptake (P_{upt}) and soil labile P pool (P_{lab}). **(D)** The CO_2 effects (%) on soil net P mineralization flux (P_{net}) and soil heterotrophic

respiration (R_{het}). M-M indicates multi-model mean, whereas OBS indicates observation. Error bars indicate the standard deviations of the multi-model means and observations, respectively.

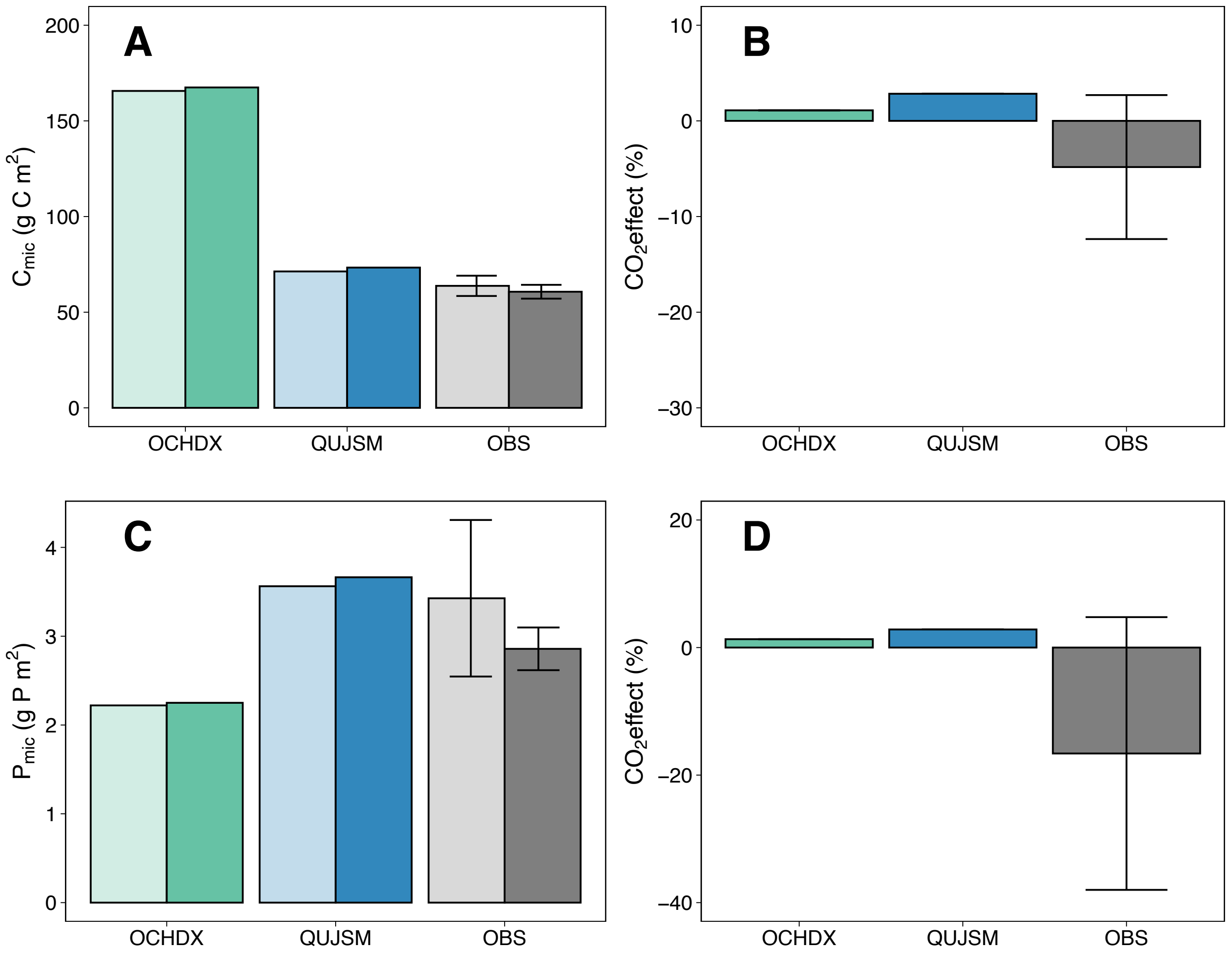

Fig. S9.

Data-model intercomparisons of the microbial carbon, nitrogen and phosphorus pools for the top 60 cm of the soil (C_{mic} and P_{mic}, respectively), (A) and (C) under both ambient and elevated CO_2 treatment, and (B) and (D) the CO_2 effect. OCHDX did not output microbial C pool.

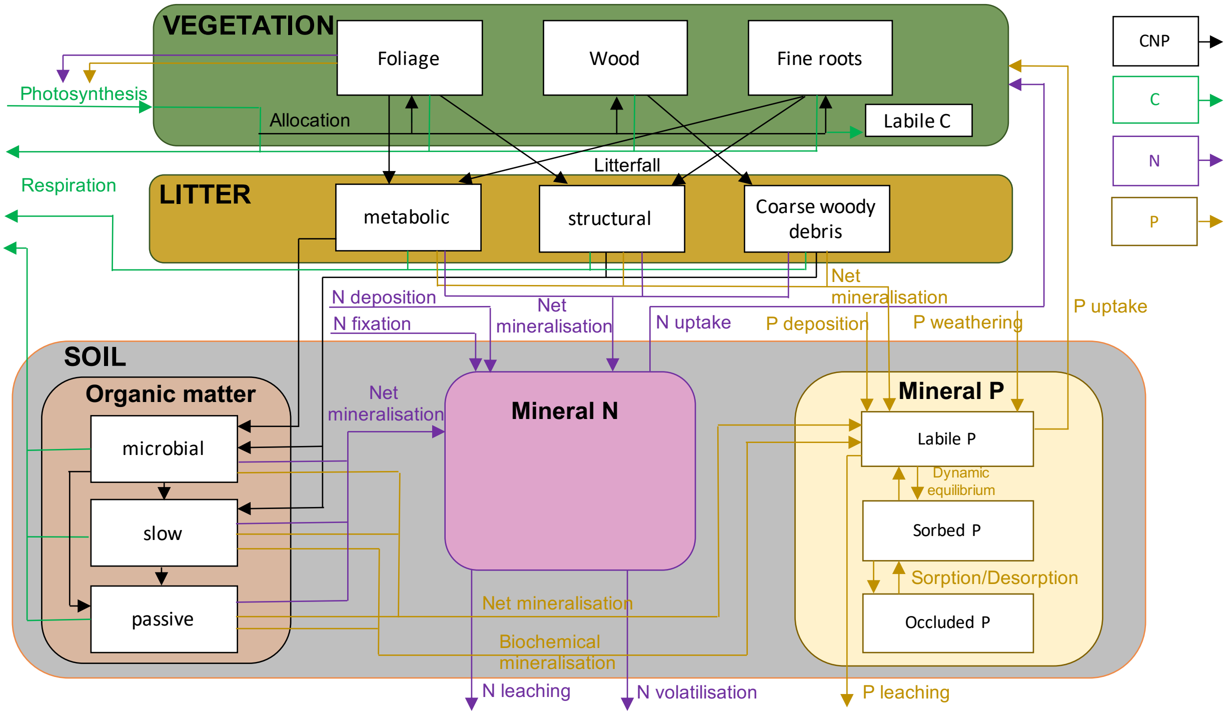

Fig. S10.

Structural diagram of CABLE-POP. Boxes indicate pools and arrowed lines indicate fluxes.

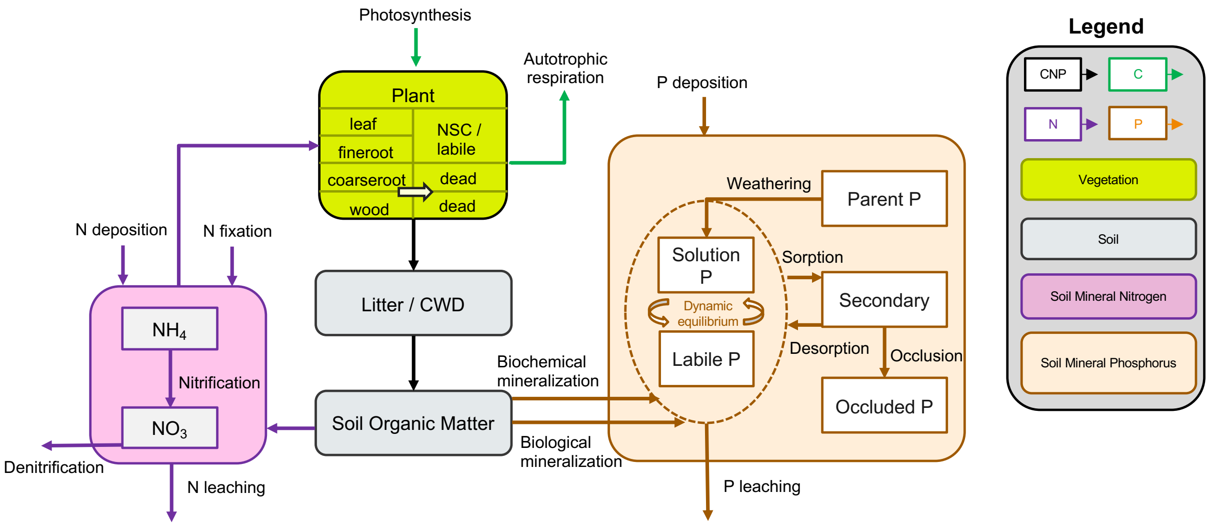

Fig. S11.

Structural diagram of ELM (v1). Boxes indicate pools and arrowed lines indicate fluxes.

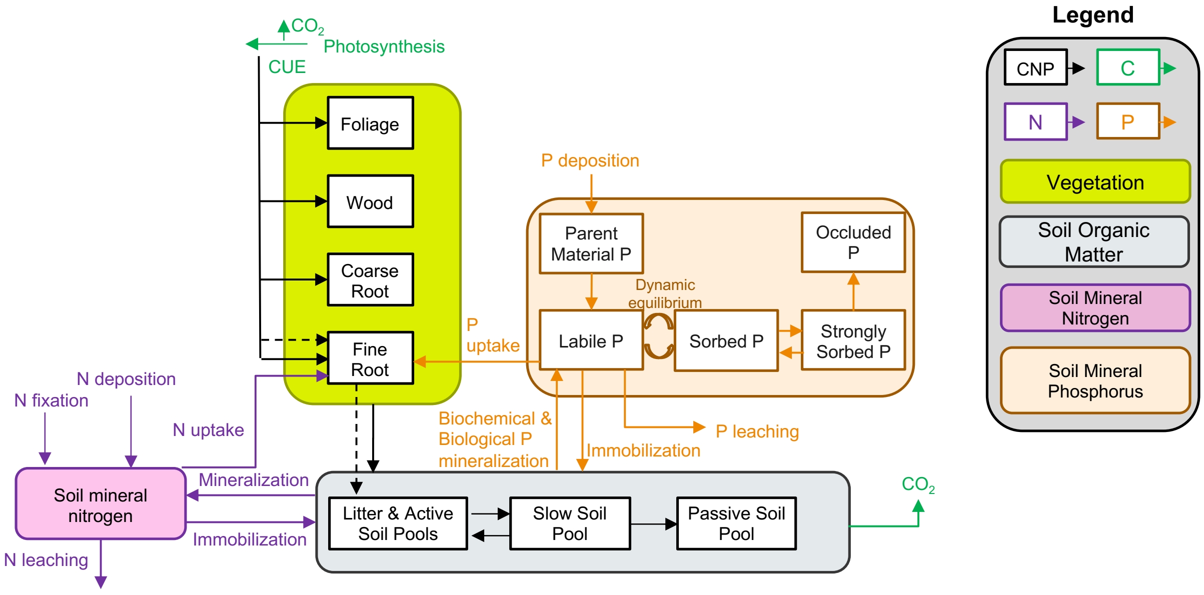

Fig. S12.

Structural diagram of GDAY-CNP. Boxes indicate pools and arrowed lines indicate fluxes. Dotted line indicates exudation flux that allocate labile carbon directly into the soil via fine root.

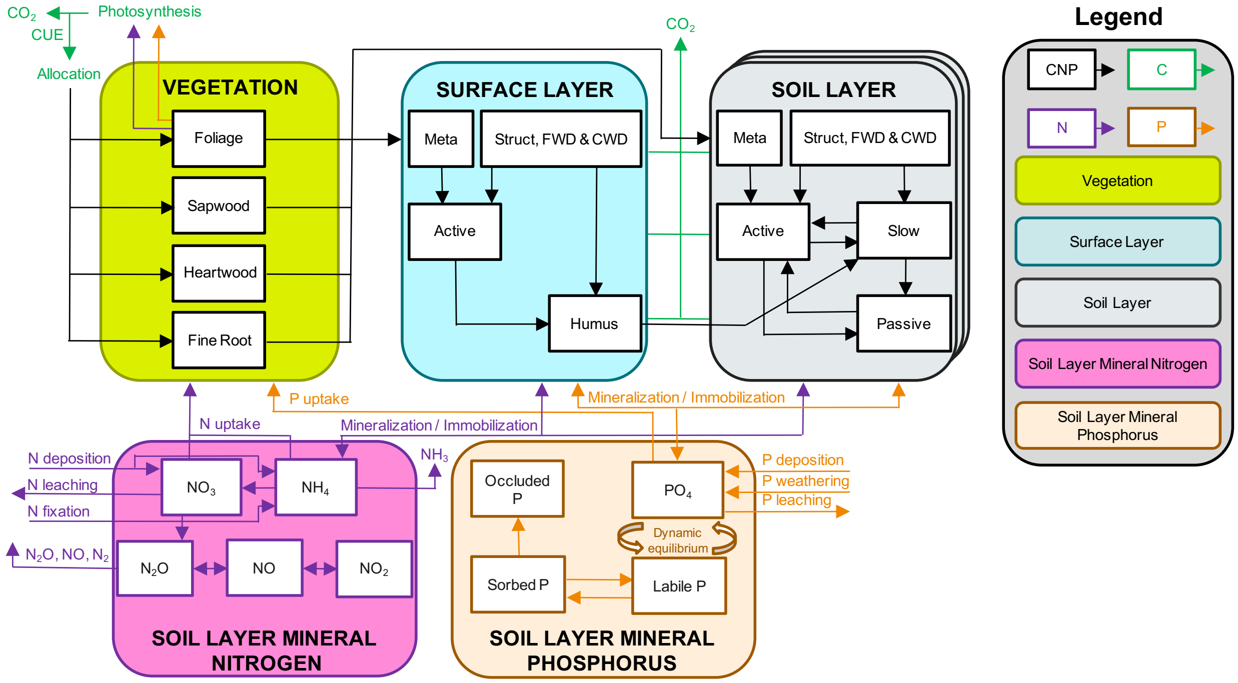

Fig. S13.

Structural diagram of LPJ-GUESS. Boxes indicate pools and arrowed lines indicate fluxes.

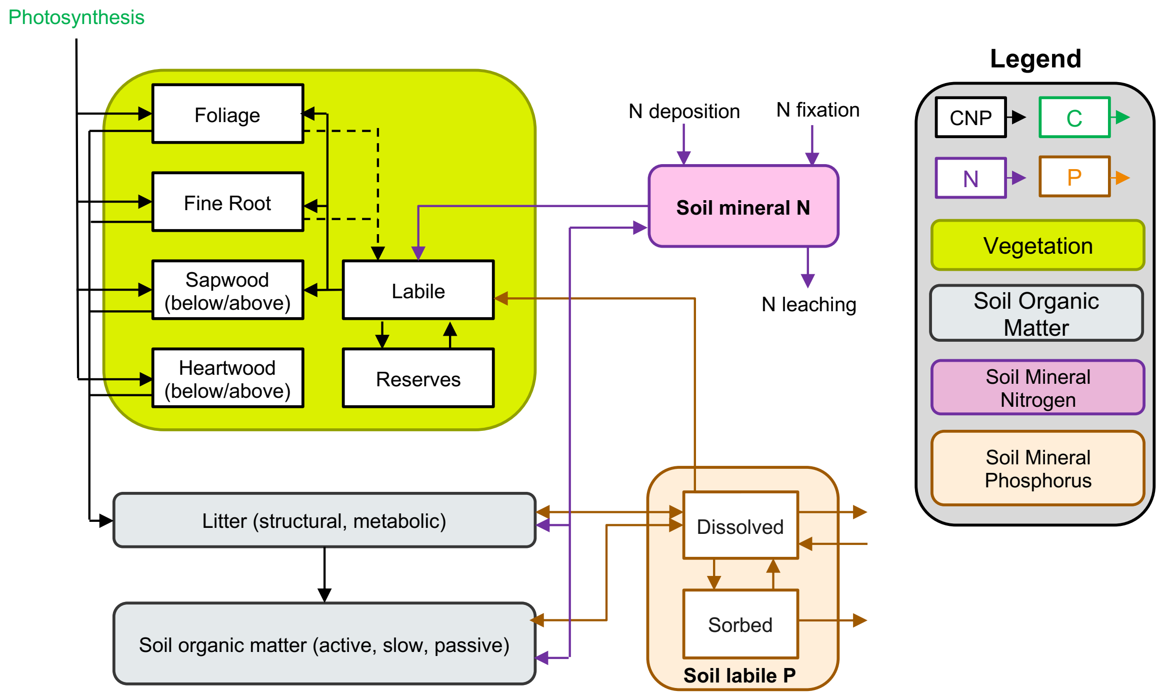

Fig. S14.

Structural diagram of ORCHIDEE-CNP. Boxes indicate pools and arrowed lines indicate fluxes.

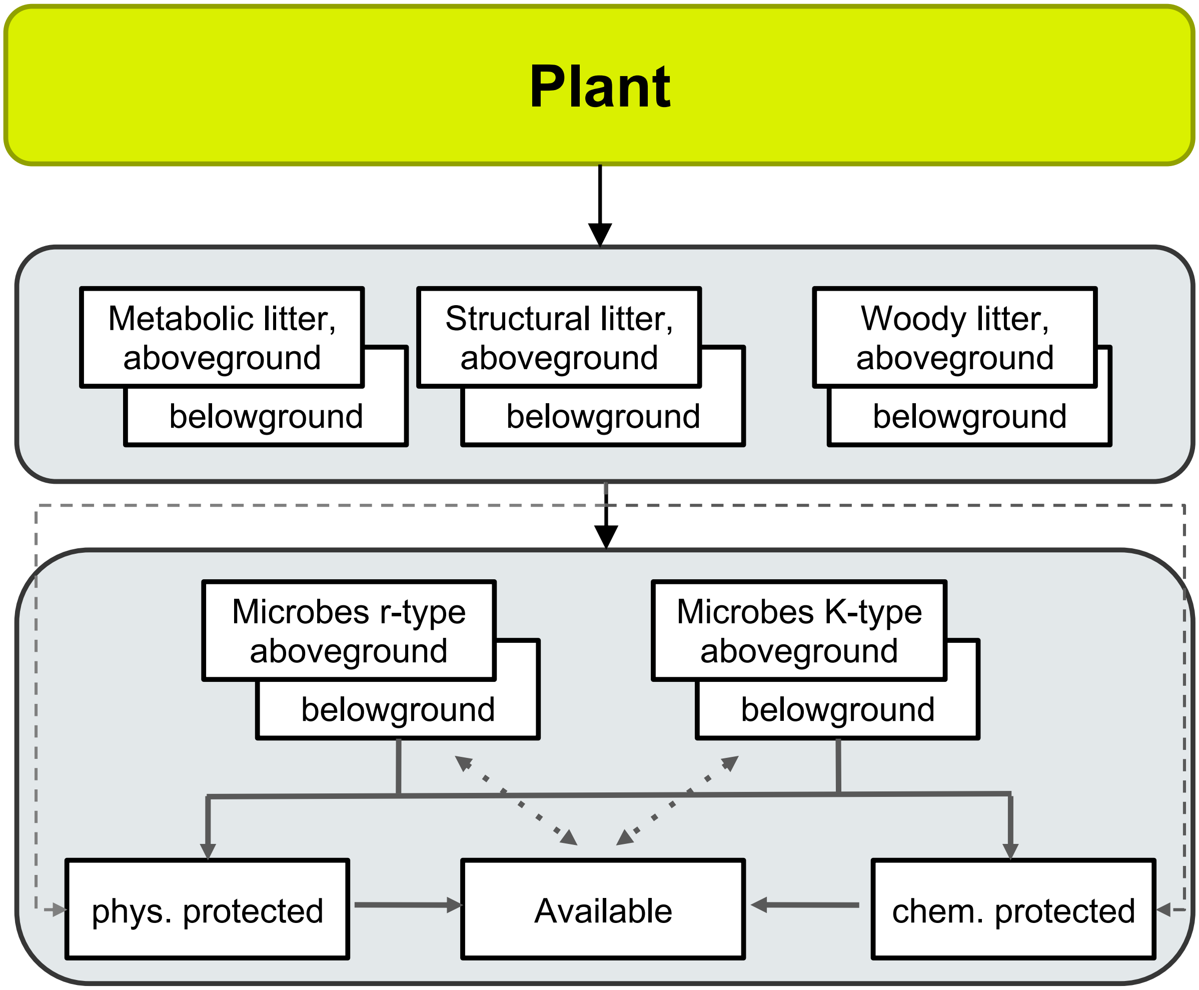

Fig. S15.

Structural diagram of ORCHIDEE-MIC. Boxes indicate pools and arrowed lines indicate fluxes. Plant pools and fluxes are simplified in this diagram.

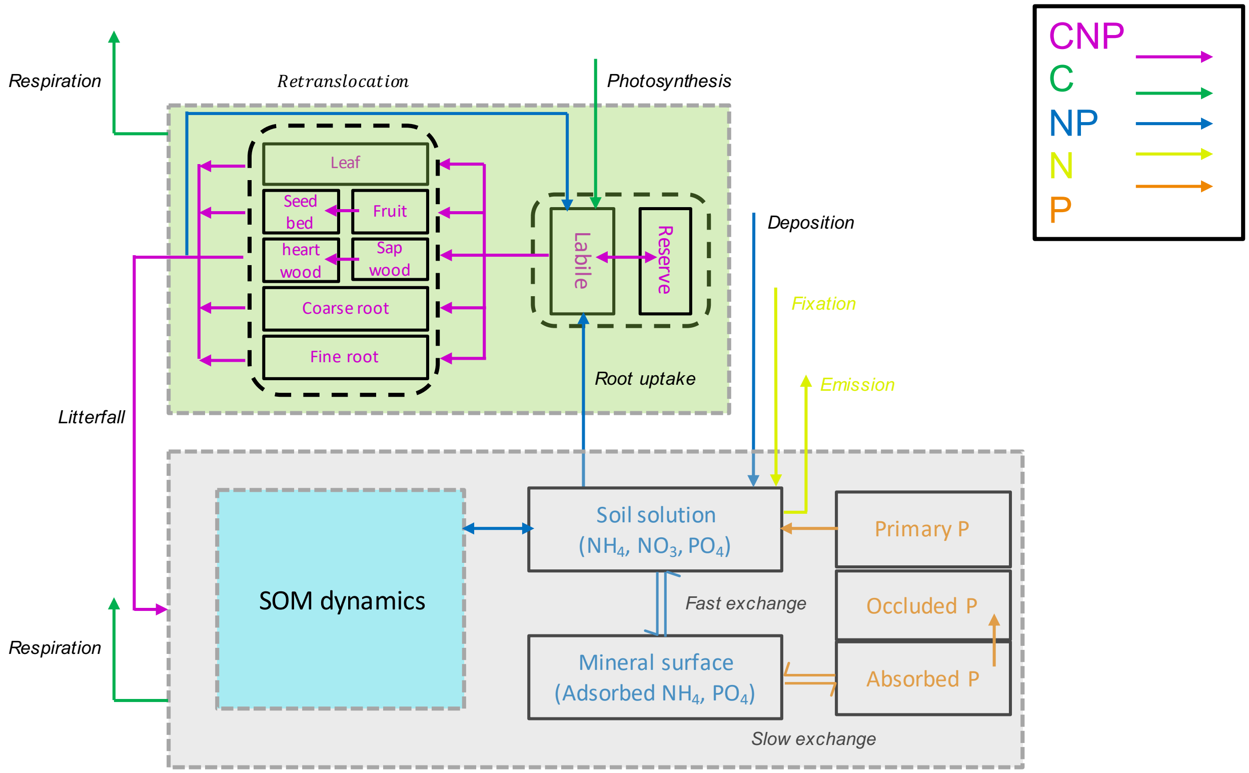

Fig. S16.

Structural diagram of QUINCY. Boxes indicate pools and arrowed lines indicate fluxes. SOM refers to soil organic matter.

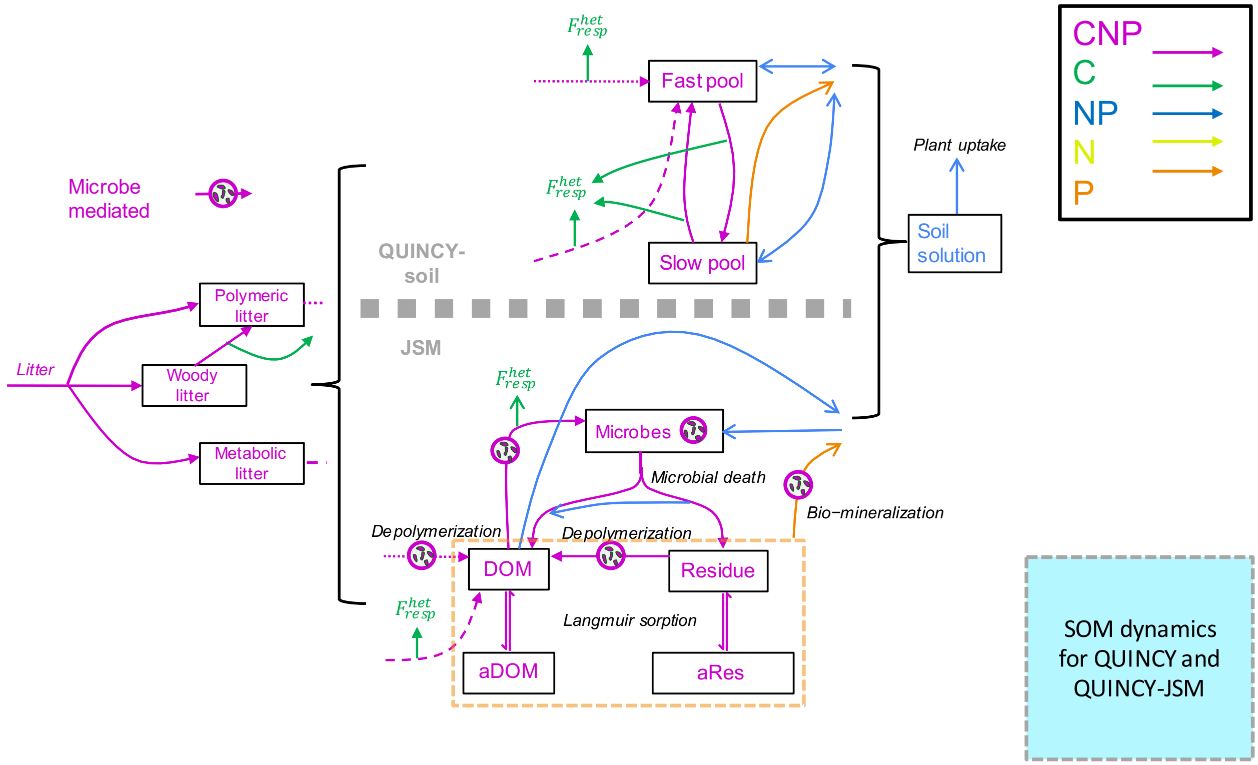

Fig. S17.

Structural diagram of JSM and its coupling with QUINCY. Boxes indicate pools and arrowed lines indicate fluxes.

Table S1.

Summary of key model structure and assumptions in this study – basic model information.

Model	CABLE-POP	ELM	GDAY-CNP	LPJ-GUESS-CNP	ORCHIDEE-CNP (V1.2)	QUINCY	ORCHIDEE-MIC	QUINCY-JSM	GDAY-CN	LPJ-GUESS-CN
**Abbreviation**	CABLP	ELMV1	GDAYP	LPJGP	OCHDP	QUINC	OCHDX	QUJSM	GDAYN	LPJGN
**Model type**	Land surface model with a dynamic vegetation module	Land surface model	Stand-scale model	Global dynamic vegetation model	Land surface model	Land surface model	Land surface model	Land surface model	Stand-scale model	Global dynamic vegetation model
**Timestep (Biogeochemistry & Biophysics)**	daily (biogeochemistry); 30 minutes (biophysics)	30 minutes	Daily	Daily	30 minutes / daily	30 minutes	30 minutes / daily	30 minutes	Daily	Daily
**Coupled biogeochemistry**	CNP	CNP	CNP	CNP	CNP	CNP	CNP	CNP	CN	CN
**Coupled microbial module**	No	No	No	No	No	No	Yes	Yes	No	No

Table S2.

Summary of key model structure and assumptions in this study – model assumptions on plant physiology.

**Abbreviation**	**CABLP**	**ELMV1**	**GDAYP**	**LPJGP**	**OCHDP**	**QUINC**	**OCHDX**	**QUJSM**	**GDAYN**	**LPJGN**
**Photosynthesis and stomatal conductance**	Farquhar and coordination of photosynthesis (10, 63, 86)	Farquhar and Collatz mixture (*63, 64*)	Farquhar (*63*)	Collatz, Haxeltine and Prentice (*63, 79*)	Farquhar (*63*)	Kull and Kruijt, Medlyn (*65, 86*)	same as OCHDP	same as QUINC	same as GDAYP	same as LPJGP
**Nutrient limitation on photosynthesis**	Wang et al. (87)	Photosynthetic capacity function of leaf N content (70)	Walker et al. (*67*)	Haxeltine and Prentice (79) for N and Ellsworth et al. (53) for NP	Ellsworth et al. (53)	Photosynthetic and Rubisco N content (*65*), and sink limitation (*66, 71*)	same as OCHDP	same as QUINC	Walker et al. (*65*)	Haxeltine and Prentice (79)
**C allocation**	Fixed fractions to leaves, wood, and fine roots	Dynamic allocation (allocation to wood increases with NPP)	Functional allometric relationship based on the pipe model and resource dependency	Functional allometric relationship based on the pipe model and resource dependency	Functional allometric relationship based on the pipe model and resource dependency	Functional allometric relationship based on the pipe model and resource dependency	same as OCHDP	same as QUINC	same as GDAYP, but without P dependent	same as LPJGP
**Maintenance respiration**	Function of Vcmax for leaves. Function of tissue N for roots and sapwood, and air/soil T (temperature)	Function of tissue N concentration and T	Fixed fraction of GPP	Function of tissue N and T	Function of tissue N and T	Function of tissue N, temperature, and water stress for leaves. Function of tissue N and autotrophic respiration T acclimation for roots and sapwood	same as OCHDP	same as QUINC	same as GDAYP	same as LPJGP

Table S3.

Summary of key model structure and assumptions in this study – model assumptions on biomass, litter and soil.

Abbreviation	CABLP	ELMV1	GDAYP	LPJGP	OCHDP	QUINC	OCHDX	QUJSM	GDAYN	LPJGN
**SOM dynamics**	CASA-CNP model	Converging trophic cascade (CTC) structure (88)	CENTURY-type model	CENTURY-type model	CENTURY-type model	CENTURY-type model	MIMICS-type model	Jena Soil Model	CENTURY-type model	CENTURY-type model
**SOM decomposition**	Decay rates controlled by max decay rate, soil T, moisture & texture	Decay rates controlled by max decay rate, soil T, moisture & nutrient availability, soil depth	Decay rates controlled by max decay rate, soil T, moisture & nutrient availability	Decay rates controlled by max decay rate, soil T, moisture, texture & nutrient	Decay rates controlled by max decay rate, soil T, moisture, texture & nutrient	First order kinetics decay rates, controlled by max rate, soil T, texture, moisture & nutrient	Decay rates controlled by microbial biomass, soil T, texture, moisture & nutrient	Michaelis-Menten kinetics, decay rates controlled by microbial biomass, soil T, moisture, texture & nutrient	Same as GDAYP	Same as LPJGP
**Soil and litter pool stoichiometry**	Varying with bounds (metabolic and coarse woody debris), and fixed (soil pools and structural litter pool)	Fixed for soil SOM pools, litter pool stoichiometry varies with PFT	Vary with bounds	Varying with bounds (soil microbial, surface microbial + hummus, slow SOM), prognostic (surface litter, woody debris & soil litter) & fixed (passive SOM)	Vary with bounds		Vary with bounds	Fixed for soil microbes; varying with bounds (metabolic and polymeric litter), and fixed (woody litter). flexible (dissolved organic matter, microbial residue, mineral-associated OC)	Same as GDAYP	Same as LPJGP
**Biomass pool stoichiometry**	Varying with bounds	Fixed	Vary without bounds	Vary without bounds	Vary without bounds	Varying with bounds	Vary without bounds	Varying with bounds	Vary without bounds	Vary without bounds
**Biomass turnover**	Fixed daily turnover; wood turnover dynamic due to changes in mortality based	Fixed annual tissue turnover rate	Fixed daily turnover rate	Fixed annual tissue turnover rate	Fixed daily turnover rate, dependent on critical leaf age	Fixed annual tissue turnover rate	Fixed daily turnover rate, dependent on critical leaf age	Fixed annual tissue turnover rate	Fixed daily turnover rate	Fixed annual tissue turnover rate

	on structural dynamics									
**Soil priming effect**	None	None	Explicit exudation to stimulate soil decomposition	Fresh litter with high nutrient content will relieve any nutrient limitation on decomposition	None	None	Fresh litter input stimulates soil C decomposition by resulting in increase of soil microbial biomass.	Through the stimulation of microbial biomass	Same as GDAYP	Same as LPJGP

Table S4.

Summary of key model structure and assumptions in this study – nitrogen cycle.

Abbreviation	CABLP	ELMV1	GDAYP	LPJGP	OCHDP	QUINC	OCHDX	QUJSM	GDAYN	LPJGN
**N fixation**	Proportional to ET	Functional of annual NPP	Proportional to ET	Proportional to AET, limited by available soil N	Dynamic function of NPP and soil inorganic N	Function of max BNF rate, soil inorganic N, & rooting depth	same as OCHDP	same as QUINC	Same as GDAYP	Same as LPJGP
**N gaseous loss**	Proportional to net N mineralization	Nitrification and denitrification based on functions of soil T, moisture, pH, and soil anoxic fraction	None	Nitrification & denitrification based on a function of soil layer microbial respiration, pH, T and anoxic fraction	Nitrification & Denitrification based on a function of microbial respiration, NO3, pH, and soil anoxic fraction		same as OCHDP	same as QUINC	Same as GDAYP	Same as LPJGP
**N leaching**	Fixed proportion of soil inorganic N	Function of NO3, drainage and runoff	Fixed proportion of soil inorganic N pool	Function of NO3 pool, percolation, and soil sand fraction	Function of soil inorganic N pool (NO3, non-sorbed NH4), deep soil water drainage, & runoff	Function of soil inorganic N pool (NO3, non-sorbed NH4), deep soil water drainage, and runoff	same as OCHDP	same as QUINC	Same as GDAYP	Same as LPJGP
**Labile plant N pool**	None	Yes	None	Max size depend on sapwood		Yes		same as QUINC	Same as GDAYP	Same as LPJGP
**Plant N retranslocation**	Constant resorption coefficients for leaves, wood and fine roots.	Constant resorption coefficient for leaf only	Constant resorption coefficient for leaf only	Max resorption coefficients for leaf, sapwood and root. Actual resorption depend on labile N pool	Constant resorption coefficient for leaf and root	Constant resorption coefficients for leaves, wood and fine roots	same as OCHDP	same as QUINC	Same as GDAYP	Same as LPJGP
**Plant N uptake**	Function of plant N demand (given by NPP) and inorganic soil N pool	Function of plant N demand and inorganic soil N pool	Function of plant N demand, root biomass and soil inorganic N pool	Function of plant N demand and status, root biomass, soil inorganic N pool & T. Cohort partitioning based on fine root surface	Function of plant N demand, root biomass, root uptake capacity and inorganic N pool	Function of plant N demand scalar, root biomass, root uptake capacity, inorganic N pool, regulated by soil temperature and moisture	same as OCHDP	similar as QUINC, further regulated by competition between soil microbes and mineral surface	Same as GDAYP	Same as LPJGP

								(for NH4)		
**Plant N demand**	Function of growth rates and tissue C:N ratios	Function of growth rates and tissue C:N ratios	Function of growth rate and tissue CN ratios	Function to optimization Vcmax in leaves (optimal C:N ratio). Affects growth rates and all tissues C:N ratios	Function of growth rates and tissue CN ratios	Function of growth rates and target growth CN ratio, which is dependent on the plant labile C&N pool	same as OCHDP	same as QUINC	Same as GDAYP	Same as LPJGP
**Plant N limitation**	Downregulation of V_{cmax} & J_{max} via leaf N, reduction of growth efficiency (GPP/LAI) & direct downregulation of NPP (excess C is stored and lost via autotrophic respiration)	Direct downregulation of NPP (excess C enters C storage pool and lost via turnover of C storage pool)	Downregulation of V_{cmax} and J_{max} via leaf N	Downregulation of V_{cmax} via leaf N	Downregulation of V_{cmax} & J_{max} via leaf N and leaf P & direct downregulation of growth taking the min of plant labile C, N & P at a given timestep (excess elements are stored)	Downregulation of V_{cmax} and J_{max} via leaf N and sink limitation of plant labile pool, and direct downregulation of NPP via autotrophic respiration	same as OCHDP	same as QUINC	Same as GDAYP	Same as LPJGP
**N effect on soil decomposition**	Litter and soil decomposition is constrained by inorganic soil N	Decomposition is constrained by soil available N	Litter and soil decomposition constrained by inorganic soil N and lignin: N ratio	Litter and SOM decomposition is constrained (reduced decay rate and changed respiration fraction) by inorganic soil N pool	Litter decomposition is constrained by inorganic soil N pool	litter and SOM decomposition is constrained by inorganic soil N pool	Yes, soil mineral N affects microbial carbon use efficiency	Soil mineral N affects microbial carbon use efficiency, microbial enzyme allocation, & competition for soluble N	Same as GDAYP	Same as LPJGP

Table S5.

Summary of key model structure and assumptions in this study – phosphorus cycle.

Abbreviation	CABLP	ELMV1	GDAYP	LPJGP	OCHDP	QUINC	OCHDX	QUJSM
**P weathering**	Prescribed parameter (soil type specific)	function of soil primary mineral P pool and soil order	Prescribed parameter (fraction of parent material P pool)	Depend on soil layer mineral to organic fraction, T, moisture, and root density	set to zero for this site	function of soil primary P pool, temperature, moisture, and root density	same as OCHDP	similar as QUINC, with additional control of microbial biomass
**P leaching**	Function of size of inorganic labile P pool	function of solution P pool, drainage and runoff	Function of size of soil inorganic labile P pool	Function of PO4 pool, percolation, and soil sand fraction	Function of size of solution P pool, drainage and runoff	Function of size of solution P pool, drainage and runoff	same as OCHDP	similar as QUINC, with additional P leaching from DOM
**Labile plant P pool**	None	Yes	None	Yes, maximum size depend on sapwood (roots for grasses)	Dynamic: Short-term and long term store buffers imbalance between P supply and demand	Yes	same as OCHDP	Yes
**Soil P pools specific to P cycle**	3 pools (labile, sorbed and strongly sorbed)	solution, labile, secondary mineral, occluded, primary mineral	5 (parent, labile, sorbed, strongly sorbed, occluded)	4 (PO4, labile, sorbed, occluded)	2 pools (labile dissolved, labile sorbed)	5 (soluble, adsorbed, absorbed, occluded, primary)	same as OCHDP	same as QUINC
**Plant P retranslocation**	Constant resorption coefficients for leaves, wood, and fine roots	Constant resorption coefficient for leaf only	Constant resorption coefficient for leaf only	Max resorption coefficients for leaf, sapwood & root. Actual depend on labile P pool	Constant resorption coefficient for leaf and root	Constant resorption coefficients for leaves, wood, and fine roots	same as OCHDP	same as QUINC
**Plant P uptake**	Function of plant P demand and soil labile P	function of plant P demand and soil solution P	Function of plant P demand, root biomass and inorganic labile P pool	Function of plant P demand and status, root biomass, soil mineral P pool & T. Cohort partitioning based on fine root surface	Function of plant P demand, root biomass, root uptake capacity, dissolved labile P pool, soil diffusivity	Function of plant P demand scalar, root biomass, root uptake capacity, soluble P pool, regulated by soil T and moisture	same as OCHDP	similar as QUINC, further regulated by competition between soil microbes and mineral surface
**Plant P demand**	Function of growth rates and tissue C:P ratios	function of growth rate of tissue CP ratios	Function of growth and tissue CP ratios	Function to optimization V_{cmax} in leaves (optimal C:P ratio). Affects growth rates and all tissues C:P ratios	Function of growth rates and tissue CP ratios	Function of growth rates and target growth NP ratio, which is dependent on the plant labile N&P pool	same as OCHDP	same as QUINC

**Plant P limitation**	Downregulation of V_{cmax} and J_{max} via leaf N:P, reduction of growth efficiency and direct downregulation of NPP (excess C is stored and lost via autotrophic respiration)	direct downregulation of NPP (excess carbon enters carbon storage pool and lost via turnover of carbon storage pool	Downregulation of V_{cmax} and J_{max} via leaf P	Downregulation of V_{cmax} via leaf P	Downregulation of V_{cmax} and J_{max} via leaf N and leaf P & direct downregulation of growth taking the min of plant labile C, N & P at a given timestep (excess elements are stored)	Downregulation of V_{cmax} and J_{max} via leaf N:P and sink limitation of plant labile pool	same as OCHDP	same as QUINC
**P effect on soil decomposition**	Litter and soil decomposition is constrained by soil labile P pool	decomposition is constrained by soil solution P		Litter and SOM decomposition is constrained (reduced decay rate and changed respiration fraction) by inorganic soil P pool	Litter decomposition is constrained by dissolved labile P pool.	None	Yes, soil mineral P affects microbial carbon use efficiency	Yes, soil mineral P affects microbial carbon use efficiency, microbial enzyme allocation, and competition for soluble P
**Soil P biochemical mineralization**	Dynamic function of soil organic P turnover rate (slow, passive pool)	function of soil organic P, the extent of N limitation and P limitation	Function of soil organic P turnover rate (slow and passive pool)	Function of soil layer organic P pool (slow pool), PO4, temperature, moisture and root density	Dynamic function of leaf N:P imbalance and substrate availability	Function of soil layer organic P pool (slow pool), temperature, and moisture	same as OCHDP	function of P in soil layer organic pool (microbial residue, mineral associated OC), microbial phosphatase abundance, soil organic pool C:P ratio, T, & moisture
**P desorption of secondary P**	None	Fixed desorption rate	Function of soil pH	Function of soil layer temperature	None	function of soil temperature and moisture	same as OCHDP	same as QUINC
**P occlusion**	Fixed fraction of strongly sorbed P pool	Fixed occlusion rate	Fixed fraction of strongly sorbed P pool	Fixed fraction of sorbed P pool	Fixed fraction of labile sorbed P	Fixed fraction of strongly sorbed P pool	same as OCHDP	same as QUINC

Table S6.

Models included in this data-model intercomparison, and their 5-character abbreviations.

**Model and version**	**5-character abbreviations**
G’DAY CN version	GDAYN
G’DAY CNP version	GDAYP
QUINCY	QUINC
ORCHIDEE CNP version	OCHDP
LPJ-Guess CN version	LPJGN
LPJ-Guess CNP version	LPJGP
CABLE-POP CNP version	CABLP
ELM CNP version	ELMXX

Table S7.

The 16 observed-to-future simulations performed in this data-model intercomparison.

ID	Scenario	Period	CO_2 concentration	Meteorological forcing	Phosphorus fertilization
01	OBS_FIX_AMB_NOP	2012-2019	Ambient (AMB)	Repeated wet-year (FIX)	No P intervention (NOP)
02	OBS_FIX_ELE_NOP	2012-2019	Elevated (ELE)	Repeated wet-year (FIX)	No P intervention (NOP)
03	OBS_VAR_AMB_NOP	2012-2019	Ambient (AMB)	Observed variable data (VAR)	No P intervention (NOP)
04	OBS_VAR_ELE_NOP	2012-2019	Elevated (ELE)	Observed variable data (VAR)	No P intervention (NOP)
05	PRD_FIX_AMB_NOP	2020-2069	Ambient (AMB)	Repeated wet-year (FIX)	No P intervention (NOP)
06	PRD_FIX_ELE_NOP	2020-2069	Elevated (ELE)	Repeated wet-year (FIX)	No P intervention (NOP)
07	PRD_FIX_AMB_MDP	2020-2069	Ambient (AMB)	Repeated wet-year (FIX)	Moderate P addition at 1.5 g P m^{-2} yr^{-1} for 2020-2022 (MDP)
08	PRD_FIX_ELE_MDP	2020-2069	Elevated (ELE)	Repeated wet-year (FIX)	Moderate P addition at 1.5 g P m^{-2} yr^{-1} for 2020-2022 (MDP)
09	PRD_FIX_AMB_HIP	2020-2069	Ambient (AMB)	Repeated wet-year (FIX)	High P addition at 3.0 g P m^{-2} yr^{-1} for 2020-2022 (MDP)
10	PRD_FIX_ELE_HIP	2020-2069	Elevated (ELE)	Repeated wet-year (FIX)	High P addition at 3.0 g P m^{-2} yr^{-1} for 2020-2022 (MDP)
11	PRD_VAR_AMB_NOP	2020-2069	Ambient (AMB)	Repeated observed data (VAR)	No P intervention (NOP)
12	PRD_VAR_ELE_NOP	2020-2069	Elevated (ELE)	Repeated observed data (VAR)	No P intervention (NOP)
13	PRD_VAR_AMB_MDP	2020-2069	Ambient (AMB)	Repeated observed data (VAR)	Moderate P addition at 1.5 g P m^{-2} yr^{-1} for 2020-2022 (MDP)

14	PRD_VAR_ELE_MDP	2020-2069	Elevated (ELE)	Repeated observed data (VAR)	Moderate P addition at 1.5 g P m^{-2} yr^{-1} for 2020-2022 (MDP)
15	PRD_VAR_AMB_HIP	2020-2069	Ambient (AMB)	Repeated observed data (VAR)	High P addition at 3.0 g P m^{-2} yr^{-1} for 2020-2022 (MDP)
16	PRD_VAR_ELE_HIP	2020-2069	Elevated (ELE)	Repeated observed data (VAR)	High P addition at 3.0 g P m^{-2} yr^{-1} for 2020-2022 (MDP)

Table S8.

Major carbon pools at EucFACE, under ambient and elevated CO_2 treatments. Unit for the pools is g C m^{-2} (ground area).

**Carbon pool**	**Ambient CO_2 treatment (mean ± SD, n = 3)**
Overstorey leaf	151 ± 14
Overstorey stem	4558 ± 321
Understorey aboveground	156 ± 20
Fine root (< 2 mm in root diameter)	76 ± 5.0
Intermediate root (2-3 mm)	151 ± 1.0
Coarse root (> 3 mm)	606 ± 60
Mycorrhizae (0 – 10 cm depth)	7.4 ± 1.6
Microbes (0 – 10 cm depth)	64 ± 5.3
Forest floor leaf litter	93 ± 18
Soil (0 – 10 cm depth)	2183 ± 280

Table S9.

Soil texture and bulk density (mean ± SD) at each specific soil depth for ambient CO_2 treatment plots at EucFACE.

Depth (cm)	Class	Ambient CO_2 treatment (g cm^{-3})
0 – 10	Loamy sand	1.41± 0.06
10 – 20	Loamy sand	1.70 ± 0.04
20 – 30	Loamy sand	1.77 ± 0.09
30-45	Loamy sand	1.70 ± 0.05
45-60	Sandy clay loam	1.77 ± 0.05
100-140	Sandy clay loam	1.82 ± 0.09
135-180	Sandy clay loam	1.69 ± 0.15
200-215	Sandy clay loam	1.82 ± 0.04
250-270	Sandy clay loam	1.76 ± 0.05
300-315	Sandy clay loam	1.74 ± 0.03
350-400	Clay	1.62 ± 0.15
400-415	Clay	1.70 ± 0.05
450-465	Clay	1.58 ± 0.02

Table S10. Soil water properties.

Variable	Unit	Ambient CO_2 treatment
Soil water table	m	~12
Total plant extractable soil water	mm	300
Root depth	m	3
Effective field capacity	mm	700
Effective wilting point	mm	400

Table S11. Soil chemical properties at each specific soil depth (mean ± SD), averaged over the data period (i.e. 2012 – 2017) across ambient plots.

**Variable**	**Depth**		
	**0 - 10 cm**	**10 - 20 cm**	**20 - 30 cm**
pH	5.52 ± 0.26	5.39 ± 0.22	5.28 ± 0.27
Total C content (%)	1.55 ± 0.45	0.71 ± 0.24	0.42 ± 0.19
Total soil N conc. (%)	0.11 ± 0.03	0.06 ± 0.02	0.03 ± 0.01
Total soil P conc. (ppm)	74.7 ± 18.0	44.8 ± 9.2	51.8 ± 17.0
Soil phosphate pool (g P m^{-2})	0.21 ± 0.03	-	-
Soil labile inorganic P pool (g P m-2)	0.17 ± 0.05	-	-
Soil labile organic P pool (g P m-2)	0.51 ± 0.02	-	-
Net N mineralization rate (g N m^{-2} yr^{-1})	8.81 ± 1.65	-	-
Net P mineralization rate (g P m^{-2} yr^{-1})	0.3 ± 0.08	-	-

Table S12. Major plant and soil C:N:P ratios under ambient CO_2 treatment (mean ± SD).

Variable	C:N ratio	C:P ratio	N:P ratio
Overstorey green mature leaf	35.5 ± 2.7	722 ± 33	22.9 ± 0.1
Overstorey leaflitter	47.8 ± 4.3	1582 ± 210	35.1 ± 2.7
Sapwood	101.6 ± 14.7	3705 ± 702	35.6 ± 2.1
Wood	110.2 ± 30.3	7696 ± 982	33.7 ± 2.7
Fine root	56.9 ± 4.6	1626 ± 81	28.7 ± 3.3
Understorey green leaf	60.0 ± 6.8	1136 ± 189	18.9 ± 1.0
Soil	13.8 ± 1.0	224 ± 39	16.4 ± 3.4
Microbes	10.0 ± 3.4	22 ± 6.1	2.0 ± 0.3

Table S13. Vegetation nitrogen and phosphorus retranslocation under ambient CO_2 treatment (mean ± SD).

Component	Nitrogen	Phosphorus
Canopy leaf	0.31 ± 0.04	0.53 ± 0.04
Stem	0.3 (assumed)	0.82 ± 0.06
Fine root	0.3 (assumed)	0.5 (assumed)
Coarse root	0.3 (assumed)	0.71 ± 0.06 (assumed)
Understorey	0.3 (assumed)	0.42 ± 0.11

Table S14. Overstorey canopy property under ambient CO_2 treatment (mean ± SD), averaged over experimental period (2012 – 2019).

Variable	Unit	Ambient CO_2 treatment
Canopy specific leaf area (SLA)	$cm^2\ g^{-1}$	55.7 ± 8.1
Up-canopy new leaf SLA	$cm^2\ g^{-1}$	64.78 ± 12.25
Low-canopy new leaf SLA	$cm^2\ g^{-1}$	73.54 ± 11.88
Up-canopy old leaf SLA	$cm^2\ g^{-1}$	48.85 ± 7.18
Low-canopy old leaf SLA	$cm^2\ g^{-1}$	57.81 ± 8.91
Canopy leaf area index (LAI)	unitless	1.72 ± 0.27
New leaf N conc.	%	1.71 ± 0.33
New leaf P conc.	%	0.106 ± 0.028
Mature leaf N conc.	%	1.6 ± 0.21
Mature leaf P conc.	%	0.071 ± 0.013
Leaf length	mm	267 (SE = 14)
Leaf lignin	%	13 – 16% dry mass
Leaf lifespan	Year	1.18
V_{cmax} at at 25 °C	$umol\ m^{-2}\ s^{-1}$	87.6 ± 4.2
J_{max} at 25 °C	$umol\ m^{-2}\ s^{-1}$	142.6 ± 7.1
Parameter g_1	unitless	3.04 ± 1.31
Parameter g_0	$mol\ m^{-2}\ s^{-1}$	0 (assumed)

Table S15. Wood property summary table.

Variable	Unit	Ambient CO_2 treatment
Wood density	g cm^{-3}	0.766 ± 0.06
Tree density	tree ha^{-1}	492 ± 85
Basal area	m^2 ha^{-1}	22.3 ± 3.7
Mean diameter	cm	23.1 ± 2.4
Average tree height	m	22.4 ± 1.8
Max. tree height of all trees	m	25.51
Average height of dominant and co-dominant trees	m	18.4
Sapwood turnover rate	yr^{-1}	1/15

Table S16. Carbon allocation coefficients to different plant components under ambient CO_2 treatment.

Allocation coefficient to	Coefficient
Leaf	0.48 ± 0.04
Stem	0.20 ± 0.03
Root	0.22 ± 0.05
Mycorrhizae	0.10 ± 0.07

Table S17. Annual soil respiration flux (g C m^{-2} yr^{-1}) for ambient CO_2 plot.

Year	Ambient
2013	1166 ± 177
2014	1065 ± 164
2015	975 ± 133

Table S18. Hourly model simulation output format.

Col	Variable	Units	Variable Name	Variable Type
1	Year	2012 to 2069	YEAR	Time
2	Day of year	1 to 365 or 366	DOY	Time
3	Hour of the day	0.5 to 24.0	HOD	Time
4	CO_2	Mean ppm	CO2h	Environ
5	Precipitation	kgH2O m-2 h-1**	PRECh	Environ
6	PAR	µmol m-2 s-1	PARh	Environ
7	Long wave radiation	W m-2	LWh	Environ
8	Air temp canopy	Mean °C	TAIRh	Environ
9	Soil temp average	Mean °C	TSOILh	Environ
10	Vapor Pres Def Mean	kPa	VPDh	Environ
11	Total soil water content	mm	SWh	Environ
12	Total plant available soil water content	mm	SWPAh	Environ
13	Net Eco Prod	gC m-2 h-1	NEPh	Flux
14	Gross Prim Prod	gC m-2 h-1	GPPh	Flux
15	Net Prim Prod	gC m-2 h-1	NPPh	Flux
16	C exudation	gC m-2 h-1	CEXh	Flux
17	C VOC Flux	gC m-2 h-1	CVOCh	Flux
18	Resp ecosystem	gC m-2 h-1	RECOh	Flux
19	Resp autotroph	gC m-2 h-1	RAUh	Flux
20	Resp leaves (maint)	gC m-2 h-1	RLh	Flux
21	Resp Wood (maint)	gC m-2 h-1	RWh	Flux
22	Resp coarse root (maint)	gC m-2 h-1	RCRh	Flux
23	Resp fine root (maint)	gC m-2 h-1	RFRh	Flux
24	Resp Growth	gC m-2 h-1	RGRh	Flux
25	Resp hetero	gC m-2 h-1	RHETh	Flux
26	Evapotranspiration	kgH2O m-2 h-1	ETh	Flux
27	Transpiration	kgH2O m-2 h-1	Th	Flux
28	Soil evaporation	kgH2O m-2 h-1	ESh	Flux
29	Canopy evaporation	kgH2O m-2 h-1	ECh	Flux
30	Soil surface runoff	kgH2O m-2 h-1	ROh	Flux
31	Drainage	kgH2O m-2 h-1	DRAINh	Flux
32	Latent Energy	W m-2	LEh	Flux
33	Sensible Heat	W m-2	SHh	Flux
34	Absorbed PAR	umol m-2 s-1	APARh	Flux
35	Canopy conductance	mol H_2O m^{-2} s^{-1}	GCh	Charac.
36	Aero. conductance	mol H_2O m^{-2} s^{-1}	GAh	Charac.
37	Leaf bound. conduct	mol H_2O m^{-2} s^{-1}	GBh	Charac.
38	Soil moisture stress	0 – 1	Betah	Charac.

Average daytime aerodynamic conductance
** $kgH_2O\ m^{-2}\ h^{-1} = mm\ h^{-1}$

Table S19. Daily model simulation output format.

Data in each daily file:

Col	Variable	Units	VariableName	Variable Type
1	Year	2013 to 2069	YEAR	Time
2	Day of the year	1 to 365 or 366	DOY	Time
3	CO2	Mean ppm	CO2	Environ
4	Precipitation	mm d-1	PREC	Environ
5	PAR	mol m-2	PAR	Environ
6	Air temp canopy	Mean °C	TAIR	Environ
7	Soil temp average	Mean °C	TSOIL	Environ
8	Vapor Pres Def	kPa h	VPD	Environ
9	Soil water content	mm	SW	Environ
10	Plant available soil water content	mm	SWPA	Environ
11	N deposition	gN m-2 d-1	NDEP	Environ
12	Net Eco Prod	gC m-2 d-1	NEP	Flux
13	Gross Prim Prod	gC m-2 d-1	GPP	Flux
14	Net Prim Prod	gC m-2 d-1	NPP	Flux
15	C exudation	gC m-2 d-1	CEX	Flux
16	C VOC Flux	gC m-2 d-1	CVOC	Flux
17	Resp ecosystem	gC m-2 d-1	RECO	Flux
18	Resp autotrophic	gC m-2 d-1	RAU	Flux
19	Resp leaves (maint)	gC m-2 d-1	RL	Flux
20	Resp wood (maint)	gC m-2 d-1	RW	Flux
21	Resp coarse root (maint)	gC m-2 d-1	RCR	Flux
22	Resp fine root (maint)	gC m-2 d-1	RFR	Flux
23	Resp growth	gC m-2 d-1	RGR	Flux
24	Resp heterotrophic	gC m-2 d-1	RHET	Flux
25	Evapotranspiration	kgH2O m-2 d-1***	ET	Flux
26	Transpiration	kgH2O m-2 d-1	TRANS	Flux
27	Soil evaporation	kgH2O m-2 d-1	ES	Flux
28	Canopy evaporation	kgH2O m-2 d-1	EC	Flux
29	Runoff	kgH2O m-2 d-1	RO	Flux
30	Drainage	kgH2O m-2 d-1	DRAIN	Flux
31	Latent energy	MJ m-2	LE	Flux
32	Sensible heat	MJ m-2	SH	Flux
33	C Leaf mass	gC m-2	CL	Pool
34	C Wood mass	gC m-2	CW	Pool
35	C Coarse root mass	gC m-2	CCR	Pool
36	C Fine root mass	gC m-2	CFR	Pool
37	C Storage (NSC)	gC m-2	CSTOR	Pool
38	C Fine litter total	gC m-2	CFLIT	Pool
39	C Fine litter above	gC m-2	CFLITA	Pool
40	C Fine litter below	gC m-2	CFLITB	Pool
41	C Coarse litter	gC m-2	CCLITB	Pool
42	C Soil total	gC m-2 0 to 30 cm	CSOIL	Pool
43	C Leaf growth	gC m-2 d-1	CGL	Flux
44	C Wood growth	gC m-2 d-1	CGW	Flux
45	C Coarse root growth	gC m-2 d-1	CGCR	Flux
46	C Fine root growth	gC m-2 d-1	CGFR	Flux
47	C reproduction growth	gC m-2 d-1	CREPR	Flux

48	C Leaf litterfall	gC m-2 d-1	CLITIN	Flux	
49	C Coarse root litter inputs	gC m-2 d-1	CCRLIN	Flux	
50	C Fine root litter input	gC m-2 d-1	CFRLIN	Flux	
51	C Wood litter fall	gC m-2 d-1	CWLIN	Flux	
52	LAI projected	m2 m-2	LAI	Characteristic	
53	Leaf gC/leaf area	gC m-2	LMA	Characteristic	
54	N Conc leaves	gN gd.m.-1	NCON	Characteristic	
55	N Mass leaves	gN m-2	NL	Pool	
56	N Mass wood	gN m-2	NW	Pool	
57	N Mass coarse roots	gN m-2	NCR	Pool	
58	N Mass fine roots	gN m-2	NFR	Pool	
59	N Storage	gN m-2	NSTOR	Pool	
60	N Fine litter total	gN m-2	NFLIT	Pool	
61	N Fine litter above	gN m-2	NFLITA	Pool	
62	N Fine litter below	gN m-2	NFLITB	Pool	
63	N Coarse litter	gN m-2	NCLITB	Pool	
64	N Soil Total	gN m-2 0 to 30cm	NSOIL	Pool	
65	N in Mineral form	gN m-2 0 to 30 cm	NPMIN	Pool	
66	N in Organic form	gN m-2 0 to 30 cm	NPORG	Pool	
67	N fixation	gN m-2 d-1	NFIX	Flux	
68	N Leaf growth	gN m-2 d-1	NGL	Flux	
69	N Wood growth	gN m-2 d-1	NGW	Flux	
70	N Coarse root growth	gN m-2 d-1	NGCR	Flux	
71	N Fine root growth	gN m-2 d-1	NGFR	Flux	
72	N Leaf litterfall	gN m-2 d-1	NLITIN	Flux	
73	N Coarse root litter input	gN m-2 d-1	NCRLIN	Flux	
74	N Fine root litter input	gN m-2 d-1	NFRLIN	Flux	
75	N Wood litterfall	gN m-2 d-1	NWLIN	Flux	
76	N Biomass Uptake	gN m-2 d-1	NUP	Flux	
77	N Gross Mineralization	gN m-2 d-1	NGMIN	Flux	
78	N Net mineralization	gN m-2 d-1	NMIN	Flux	
79	N Volatilization	gN m-2 d-1	NVOL	Flux	
80	N Leaching	gN m-2 d-1	NLEACH	Flux	
81	N Leaf retranslocation	gN m-2 d-1	NLRETR	Flux	
82	N Wood retrans .	gN m-2 d-1	NWRETR	Flux	
83	N Coarse root retrans.	gN m-2 d-1	NCRRETR	Flux	
84	N Fine root retrans.	gN m-2 d-1	NFRRETR	Flux	
85	Absorbed PAR	MJ m-2 d-1	APARd	Flux	
86	Average daytime canopy conductance		mol H_2O m-2 s-1	GCd	Charac.
87	Average daytime aerodynamic conductance		mol H_2O m-2 s-1	GAd	Charac.
88	Average daytime leaf boundary conductance		mol H_2O m-2 s-1	GBd	Charac.
89	Soil moisture stress	0 – 1	Betad	Charac.	
90	P Mass leaves	gP m-2	PL	Pool	
91	P Mass wood	gP m-2	PW	Pool	
92	P Mass coarse roots	gP m-2	PCR	Pool	
93	P Mass fine roots	gP m-2	PFR	Pool	
94	P Storage	gP m-2	PSTOR	Pool	
95	P Fine litter total	gP m-2	PFLIT	Pool	
96	P Fine litter above	gP m-2	PFLITA	Pool	
97	P Fine litter below	gP m-2	PFLITB	Pool	
98	P Coarse litter	gP m-2	PCLITB	Pool	

99	P Soil total	gP m-2 0 to 30cm	PSOIL	Pool
100	P in Labile form	gP m-2 0 to 30 cm	PLAB	Pool
101	P in Secondary form	gP m-2 0 to 30 cm	PSEC	Pool
102	P in Occluded form	gP m-2 0 to 30 cm	POCC	Pool
103	P in Parent mat. form	gP m-2 0 to 30 cm	PPAR	Pool
104	P in Mineral form	gP m-2 0 to 30 cm	PPMIN	Pool
105	P in Organic form	gP m-2 0 to 30 cm	PPORG	Pool
106	P Leaf litterfall	gP m-2 d-1	PLITIN	Flux
107	P Coarse root litter input	gP m-2 d-1	PCRLIN	Flux
108	P Fine root litter input	gP m-2 d-1	PFRLIN	Flux
109	P Wood litterfall	gP m-2 d-1	PWLIN	Flux
110	P Biomass uptake	gP m-2 d-1	PUP	Flux
111	P Gross mineralization	gP m-2 d-1	PGMIN	Flux
112	P Net mineralization	gP m-2 d-1	PMIN	Flux
113	P Biochem. mineral.	gP mo2 d-1	PBIOCHMIN	Flux
114	P Leaching	gP m-2 d-1	PLEACH	Flux
115	P Leaf growth	gP m-2 d-1	PGL	Flux
116	P Wood growth	gP m-2 d-1	PGW	Flux
117	P Coarse root growth	gP m-2 d-1	PGCR	Flux
118	P Fine root growth	gP m-2 d-1	PGFR	Flux
119	P Leaf retranslocation	gP m-2 d-1	PLRETR	Flux
120	P Wood retrans.	gP m-2 d-1	PWRETR	Flux
121	P Coarse root retrans.	gP m-2 d-1	PCRRETR	Flux
122	P Fine root retrans.	gP m-2 d-1	PFRRETR	Flux
123	P weathering rate.	gP m-2 d-1	PWEA	Flux
124	P deposition rate.	gP m-2 d-1	PDEP	Flux

** $kgH_2O\ m^{-2}\ d^{-1} = mm\ d^{-1}$

124+n Please add additional variables that are unique to your model but could be important (e.g. potential GPP).

REFERENCES AND NOTES

1. V. K. Arora, A. Katavouta, R. G. Williams, C. D. Jones, V. Brovkin, P. Friedlingstein, J. Schwinger, L. Bopp, O. Boucher, P. Cadule, M. A. Chamberlain, J. R. Christian, C. Delire, R. A. Fisher, T. Hajima, T. Ilyina, E. Joetzjer, M. Kawamiya, C. D. Koven, J. P. Krasting, R. M. Law, D. M. Lawrence, A. Lenton, K. Lindsay, J. Pongratz, T. Raddatz, R. Séférian, K. Tachiiri, J. F. Tjiputra, A. Wiltshire, T. Wu, T. Ziehn, Carbon–concentration and carbon–climate feedbacks in CMIP6 models and their comparison to CMIP5 models. *Biogeosciences* **17**, 4173–4222 (2020).

2. P. Friedlingstein, M. W. Jones, M. O'Sullivan, R. M. Andrew, D. C. E. Bakker, J. Hauck, C. Le Quéré, G. P. Peters, W. Peters, J. Pongratz, S. Sitch, J. G. Canadell, P. Ciais, R. B. Jackson, S. R. Alin, P. Anthoni, N. R. Bates, M. Becker, N. Bellouin, L. Bopp, T. T. T. Chau, F. Chevallier, L. P. Chini, M. Cronin, K. I. Currie, B. Decharme, L. M. Djeutchouang, X. Dou, W. Evans, R. A. Feely, L. Feng, T. Gasser, D. Gilfillan, T. Gkritzalis, G. Grassi, L. Gregor, N. Gruber, Ö. Gürses, I. Harris, R. A. Houghton, G. C. Hurtt, Y. Iida, T. Ilyina, I. T. Luijkx, A. Jain, S. D. Jones, E. Kato, D. Kennedy, K. Klein Goldewijk, J. Knauer, J. I. Korsbakken, A. Körtzinger, P. Landschützer, S. K. Lauvset, N. Lefèvre, S. Lienert, J. Liu, G. Marland, P. C. McGuire, J. R. Melton, D. R. Munro, J. E. M. S. Nabel, S.-I. Nakaoka, Y. Niwa, T. Ono, D. Pierrot, B. Poulter, G. Rehder, L. Resplandy, E. Robertson, C. Rödenbeck, T. M. Rosan, J. Schwinger, C. Schwingshackl, R. Séférian, A. J. Sutton, C. Sweeney, T. Tanhua, P. P. Tans, H. Tian, B. Tilbrook, F. Tubiello, G. R. van der Werf, N. Vuichard, C. Wada, R. Wanninkhof, A. J. Watson, D. Willis, A. J. Wiltshire, W. Yuan, C. Yue, X. Yue, S. Zaehle, J. Zeng, Global Carbon Budget 2021. *Earth Syst. Sci. Data*. **14**, 1917–2005 (2022)

3. W. R. Wieder, C. C. Cleveland, W. K. Smith, K. Todd-Brown, Future productivity and carbon storage limited by terrestrial nutrient availability. *Nat. Geosci.* **8**, 441–444 (2015).

4. Q. Zhang, Y. P. Wang, R. J. Matear, A. J. Pitman, Y. J. Dai, Nitrogen and phosphorous limitations significantly reduce future allowable CO_2 emissions. *Geophys. Res. Lett.* **41**, 632–637 (2014).

5. M. Hawkesford, I. Cakmak, D. Coskun, L. J. De Kok, H. Lambers, J. K. Schjoerring, P. J. White, "Chapter 6: Functions of macronutrients" in *Marschner's Mineral Nutrition of Plants* (Elsevier, ed. 4, 2023), pp. 201–228.

6. E. Hou, Y. Luo, Y. Kuang, C. Chen, X. Lu, L. Jiang, X. Luo, D. Wen, Global meta-analysis shows pervasive phosphorus limitation of aboveground plant production in natural terrestrial ecosystems. *Nat. Commun.* **11**, 637 (2020).

7. E. Du, C. Terrer, A. F. A. Pellegrini, A. Ahlström, C. J. van Lissa, X. Zhao, N. Xia, X. Wu, R. B. Jackson, Global patterns of terrestrial nitrogen and phosphorus limitation. *Nat. Geosci.* **13**, 221–226 (2020).

8. Y.-P. Wang, B. Z. Houlton, C. B. Field, A model of biogeochemical cycles of carbon, nitrogen, and phosphorus including symbiotic nitrogen fixation and phosphatase production. *Global Biogeochem. Cycles* **21**, GB1018 (2007).

9. X. Yang, P. E. Thornton, D. M. Ricciuto, W. M. Post, The role of phosphorus dynamics in tropical forests—A modeling study using CLM-CNP. *Biogeosciences* **11**, 1667–1681 (2014).

10. V. Haverd, B. Smith, L. Nieradzik, P. R. Briggs, W. Woodgate, C. M. Trudinger, J. G. Canadell, M. Cuntz, A new version of the CABLE land surface model (Subversion revision r4601) incorporating land use and land cover change, woody vegetation demography, and a novel optimisation-based approach to plant coordination of photosynthesis. *Geosci. Model Dev.* **11**, 2995–3026 (2018).

11. D. S. Goll, N. Vuichard, F. Maignan, A. Jornet-Puig, J. Sardans, A. Violette, S. Peng, Y. Sun, M. Kvakic, M. Guimberteau, B. Guenet, S. Zaehle, J. Penuelas, I. Janssens, P. Ciais, A representation of the phosphorus cycle for ORCHIDEE (revision 4520). *Geosci. Model Dev.* **10**, 3745–3770 (2017).

12. Q. Zhu, W. J. Riley, J. Tang, N. Collier, F. M. Hoffman, X. Yang, G. Bisht, Representing nitrogen, phosphorus, and carbon interactions in the E3SM land model: Development and global benchmarking. *J. Adv. Model. Earth Syst.* **11**, 2238–2258 (2019).

13. T. Thum, S. Caldararu, J. Engel, M. Kern, M. Pallandt, R. Schnur, L. Yu, S. Zaehle, A new model of the coupled carbon, nitrogen, and phosphorus cycles in the terrestrial biosphere (QUINCY v1.0; revision 1996). *Geosci. Model Dev.* **12**, 4781–4802 (2019).

14. Q. Zhang, Y. P. Wang, A. J. Pitman, Y. J. Dai, Limitations of nitrogen and phosphorous on the terrestrial carbon uptake in the 20th century. *Geophys. Res. Lett.* **38**, L22701 (2011).

15. D. S. Goll, V. Brovkin, B. R. Parida, C. H. Reick, J. Kattge, P. B. Reich, P. M. van Bodegom, Ü. Niinemets, Nutrient limitation reduces land carbon uptake in simulations with a model of combined carbon, nitrogen and phosphorus cycling. *Biogeosciences* **9**, 3547–3569 (2012).

16. T. Ziehn, Y.-P. Wang, Y. Huang, Land carbon-concentration and carbon-climate feedbacks are significantly reduced by nitrogen and phosphorus limitation. *Environ. Res. Lett.* **16**, 074043 (2021).

17. B. E. Medlyn, M. G. De Kauwe, S. Zaehle, A. P. Walker, R. A. Duursma, K. Luus, M. Mishurov, B. Pak, B. Smith, Y. Wang, X. Yang, K. Y. Crous, J. E. Drake, T. E. Gimeno, C. A. Macdonald, R. J. Norby, S. A. Power, M. G. Tjoelker, D. S. Ellsworth, Using models to guide field experiments: A priori predictions for the CO_2 response of a nutrient- and water-limited native Eucalypt woodland. *Glob. Chang. Biol.* **22**, 2834–2851 (2016).

18. K. Fleischer, A. Rammig, M. G. De Kauwe, A. P. Walker, T. F. Domingues, L. Fuchslueger, S. Garcia, D. S. Goll, A. Grandis, M. Jiang, V. Haverd, F. Hofhansl, J. A. Holm, B. Kruijt, F. Leung, B. E. Medlyn, L. M. Mercado, R. J. Norby, B. Pak, C. von Randow, C. A. Quesada, K. J. Schaap, O. J. Valverde-Barrantes, Y.-P. Wang, X. Yang, S. Zaehle, Q. Zhu, D. M. Lapola, Amazon forest response to CO_2 fertilization dependent on plant phosphorus acquisition. *Nat. Geosci.* **12**, 736–741 (2019).

19. L. Yu, B. Ahrens, T. Wutzler, M. Schrumpf, S. Zaehle, Jena Soil Model (JSM v1.0; revision 1934): A microbial soil organic carbon model integrated with nitrogen and phosphorus processes. *Geosci. Model Dev.* **13**, 783–803 (2020).

20. X. Yang, D. M. Ricciuto, P. E. Thornton, X. Shi, M. Xu, F. Hoffman, R. J. Norby, The effects of phosphorus cycle dynamics on carbon sources and sinks in the Amazon region: A modeling study using ELM v1. *J. Geophys. Res. Biogeo.* **124**, 3686–3698 (2019).

21. D. S. Ellsworth, I. C. Anderson, K. Y. Crous, J. Cooke, J. E. Drake, A. N. Gherlenda, T. E. Gimeno, C. A. Macdonald, B. E. Medlyn, J. R. Powell, M. G. Tjoelker, P. B. Reich, Elevated CO_2 does not increase eucalypt forest productivity on a low-phosphorus soil. *Nat. Clim. Change* **7**, 279–282 (2017).

22. K. Y. Crous, A. Ósvaldsson, D. S. Ellsworth, Is phosphorus limiting in a mature Eucalyptus woodland? Phosphorus fertilisation stimulates stem growth. *Plant Soil* **391**, 293–305 (2015).

23. M. Jiang, B. E. Medlyn, J. E. Drake, R. A. Duursma, I. C. Anderson, C. V. M. Barton, M. M. Boer, Y. Carrillo, L. Castañeda-Gómez, L. Collins, K. Y. Crous, M. G. De Kauwe, B. M. dos Santos, K. M. Emmerson, S. L. Facey, A. N. Gherlenda, T. E. Gimeno, S. Hasegawa, S. N. Johnson, A. Kännaste, C. A. Macdonald, K. Mahmud, B. D. Moore, L. Nazaries, E. H. J. Neilson, U. N. Nielsen, Ü. Niinemets, N. J. Noh, R. Ochoa-Hueso, V. S. Pathare, E. Pendall, J. Pihlblad, J. Piñeiro, J. R. Powell, S. A. Power, P. B. Reich, A. A. Renchon, M. Riegler, R. Rinnan, P. D. Rymer, R. L. Salomón, B. K. Singh, B. Smith, M. G. Tjoelker, J. K. M. Walker, A. Wujeska-Klause, J. Yang, S. Zaehle, D. S. Ellsworth, The fate of carbon in a mature forest under carbon dioxide enrichment. *Nature* **580**, 227–231 (2020).

24. J. Yang, B. E. Medlyn, M. G. De Kauwe, R. A. Duursma, M. Jiang, D. Kumarathunge, K. Y. Crous, T. E. Gimeno, A. Wujeska-Klause, D. S. Ellsworth, Low sensitivity of gross primary production to elevated CO_2 in a mature eucalypt woodland. *Biogeosciences* **17**, 265–279 (2020).

25. C. E. Prescott, S. J. Grayston, H.-S. Helmisaari, E. Kaštovská, C. Körner, H. Lambers, I. C. Meier, P. Millard, I. Ostonen, Surplus carbon drives allocation and plant–soil interactions. *Trends Ecol. Evol.* **35**, 1110–1118 (2020).

26. L. Castañeda-Gómez, J. K. M. Walker, J. R. Powell, D. S. Ellsworth, E. Pendall, Y. Carrillo, Impacts of elevated carbon dioxide on carbon gains and losses from soil and associated microbes in a Eucalyptus woodland. *Soil Biol. Biochem.* **143**, 107734 (2020).

27. H. N. Comins, R. E. McMurtrie, Long-term response of nutrient-limited forests to CO_2 enrichment; equilibrium behavior of plant-soil models. *Ecol. Appl.* **3**, 666–681 (1993).

28. M. Jiang, S. Zaehle, M. G. De Kauwe, A. P. Walker, S. Caldararu, D. S. Ellsworth, B. E. Medlyn, The quasi-equilibrium framework revisited: Analyzing long-term CO_2 enrichment responses in plant–soil models. *Geosci. Model Dev.* **12**, 2069–2089 (2019).

29. B. Smith, D. Wårlind, A. Arneth, T. Hickler, P. Leadley, J. Siltberg, S. Zaehle, Implications of incorporating N cycling and N limitations on primary production in an individual-based dynamic vegetation model. *Biogeosciences* **11**, 2027–2054 (2014).

30. H. Zhang, D. S. Goll, Y. Wang, P. Ciais, W. R. Wieder, R. Abramoff, Y. Huang, B. Guenet, A. Prescher, R. A. Viscarra Rossel, P. Barré, C. Chenu, G. Zhou, X. Tang, Microbial dynamics and soil physicochemical properties explain large-scale variations in soil organic carbon. *Glob. Chang. Biol.* **26**, 2668–2685 (2020).

31. B. E. Medlyn, S. Zaehle, M. G. De Kauwe, A. P. Walker, M. C. Dietze, P. J. Hanson, T. Hickler, A. K. Jain, Y. Luo, W. Parton, I. C. Prentice, P. E. Thornton, S. Wang, Y.-P. Wang, E. Weng, C. M. Iversen, H. R. McCarthy, J. M. Warren, R. Oren, R. J. Norby, Using ecosystem experiments to improve vegetation models. *Nat. Clim. Change* **5**, 528–534 (2015).

32. R. A. Duursma, T. E. Gimeno, M. M. Boer, K. Y. Crous, M. G. Tjoelker, D. S. Ellsworth, Canopy leaf area of a mature evergreen Eucalyptus woodland does not respond to elevated atmospheric [CO_2] but tracks water availability. *Glob. Chang. Biol.* **22**, 1666–1676 (2016).

33. J. E. Drake, C. A. Macdonald, M. G. Tjoelker, K. Y. Crous, T. E. Gimeno, B. K. Singh, P. B. Reich, I. C. Anderson, D. S. Ellsworth, Short-term carbon cycling responses of a mature eucalypt woodland to gradual stepwise enrichment of atmospheric CO_2 concentration. *Glob. Chang. Biol.* **22**, 380–390 (2016).

34. S. Hasegawa, C. A. Macdonald, S. A. Power, Elevated carbon dioxide increases soil nitrogen and phosphorus availability in a phosphorus-limited Eucalyptus woodland. *Glob. Chang. Biol.* **22**, 1628–1643 (2016).

35. R. Ochoa-Hueso, J. Hughes, M. Delgado-Baquerizo, J. E. Drake, M. G. Tjoelker, J. Piñeiro, S. A. Power, Rhizosphere-driven increase in nitrogen and phosphorus availability under elevated atmospheric CO_2 in a mature Eucalyptus woodland. *Plant Soil* **416**, 283–295 (2017).

36. J. E. Drake, A. Gallet-Budynek, K. S. Hofmockel, E. S. Bernhardt, S. A. Billings, R. B. Jackson, K. S. Johnsen, J. Lichter, H. R. McCarthy, M. L. McCormack, D. J. P. Moore, R. Oren, S. Palmroth, R. P. Phillips, J. S. Pippen, S. G. Pritchard, K. K. Treseder, W. H. Schlesinger, E. H. DeLucia, A. C. Finzi, Increases in the flux of carbon belowground stimulate nitrogen uptake and sustain the long-term enhancement of forest productivity under elevated CO_2. *Ecol. Lett.* **14**, 349–357 (2011).

37. R. P. Phillips, I. C. Meier, E. S. Bernhardt, A. S. Grandy, K. Wickings, A. C. Finzi, Roots and fungi accelerate carbon and nitrogen cycling in forests exposed to elevated CO_2. *Ecol. Lett.* **15**, 1042–1049 (2012).

38. J. Piñeiro, R. Ochoa-Hueso, J. E. Drake, M. G. Tjoelker, S. A. Power, Water availability drives fine root dynamics in a Eucalyptus woodland under elevated atmospheric CO_2 concentration. *Funct. Ecol.* **34**, 2389–2402 (2020).

39. M. Jiang, K. Y. Crous, Y. Carrillo et al., Microbial competition for phosphorus limits the CO2 response of a mature forest. *Nature* (2024). https://doi.org/10.1038/s41586-024-07491-0

40. K. Y. Crous, A. Wujeska-Klause, M. Jiang, B. E. Medlyn, D. S. Ellsworth, Nitrogen and phosphorus retranslocation of leaves and stemwood in a mature Eucalyptus forest exposed to 5 years of elevated CO_2. *Front. Plant Sci.* **10**, 664 (2019).

41. T. Reichert, A. Rammig, L. Fuchslueger, L. F. Lugli, C. A. Quesada, K. Fleischer, Plant phosphorus-use and -acquisition strategies in Amazonia. *New Phytol.* **234**, 1126–1143 (2022).

42. I. C. Prentice, X. Liang, B. E. Medlyn, Y.-P. Wang, Reliable, robust and realistic: The three R's of next-generation land-surface modelling. *Atmos. Chem. Phys.* **15**, 5987–6005 (2015).

43. R. A. Fisher, C. D. Koven, Perspectives on the future of land surface models and the challenges of representing complex terrestrial systems. *J. Adv. Model. Earth Syst.* **12**, e2018MS001453 (2020).

44. Y. Luo, T. F. Keenan, M. Smith, Predictability of the terrestrial carbon cycle. *Glob. Chang. Biol.* **21**, 1737–1751 (2015).

45. S. C. Reed, X. Yang, P. E. Thornton, Incorporating phosphorus cycling into global modeling efforts: A worthwhile, tractable endeavor. *New Phytol.* **208**, 324–329 (2015).

46. D. L. Achat, L. Augusto, A. Gallet-Budynek, D. Loustau, Future challenges in coupled C–N–P cycle models for terrestrial ecosystems under global change: A review. *Biogeochemistry* **131**, 173–202 (2016).

47. M. Jiang, S. Caldararu, S. Zaehle, D. S. Ellsworth, B. E. Medlyn, Towards a more physiological representation of vegetation phosphorus processes in land surface models. *New Phytol.* **222**, 1223–1229 (2019).

48. R. J. Norby, M. G. De Kauwe, T. F. Domingues, R. A. Duursma, D. S. Ellsworth, D. S. Goll, D. M. Lapola, K. A. Luus, A. R. MacKenzie, B. E. Medlyn, R. Pavlick, A. Rammig, B. Smith, R. Thomas, K. Thonicke, A. P. Walker, X. Yang, S. Zaehle, Model–data synthesis for the next generation of forest free-air CO_2 enrichment (FACE) experiments. *New Phytol.* **209**, 17–28 (2016).

49. A. P. Walker, P. J. Hanson, M. G. De Kauwe, B. E. Medlyn, S. Zaehle, S. Asao, M. Dietze, T. Hickler, C. Huntingford, C. M. Iversen, A. Jain, M. Lomas, Y. Luo, H. McCarthy, W. J. Parton, I. C. Prentice, P. E. Thornton, S. Wang, Y.-P. Wang, D. Warlind, E. Weng, J. M. Warren, F. I. Woodward, R. Oren, R. J. Norby, Comprehensive ecosystem model-data synthesis using multiple data sets at two temperate forest free-air CO_2 enrichment experiments: Model performance at ambient CO_2 concentration. *J. Geophys. Res. Biogeo.* **119**, 937–964 (2014).

50. M. G. De Kauwe, B. E. Medlyn, S. Zaehle, A. P. Walker, M. C. Dietze, Y. Wang, Y. Luo, A. K. Jain, B. El-Masri, T. Hickler, D. Wårlind, E. Weng, W. J. Parton, P. E. Thornton, S. Wang, I. C. Prentice, S. Asao, B. Smith, H. R. McCarthy, C. M. Iversen, P. J. Hanson, J. M. Warren, R. Oren, R. J. Norby, Where does the carbon go? A model-data intercomparison of vegetation carbon allocation and turnover processes at two temperate forest free-air CO_2 enrichment sites. *New Phytol.* **203**, 883–899 (2014).

51. S. Zaehle, B. E. Medlyn, M. G. De Kauwe, A. P. Walker, M. C. Dietze, T. Hickler, Y. Luo, Y. Wang, B. El-Masri, P. Thornton, A. Jain, S. Wang, D. Warlind, E. Weng, W. Parton, C. M. Iversen, A. Gallet-Budynek, H. McCarthy, A. Finzi, P. J. Hanson, I. C. Prentice, R. Oren, R. J. Norby, Evaluation of 11 terrestrial carbon–nitrogen cycle models against observations from two temperate Free-Air CO_2 Enrichment studies. *New Phytol.* **202**, 803–822 (2014).

52. A. Rogers, B. E. Medlyn, J. S. Dukes, G. Bonan, S. Caemmerer, M. C. Dietze, J. Kattge, A. D. B. Leakey, L. M. Mercado, Ü. Niinemets, I. C. Prentice, S. P. Serbin, S. Sitch, D. A. Way, S. Zaehle, A

roadmap for improving the representation of photosynthesis in Earth system models. *New Phytol.* **213**, 22–42 (2017).

53. D. S. Ellsworth, K. Y. Crous, M. G. De Kauwe, L. T. Verryckt, D. Goll, S. Zaehle, K. J. Bloomfield, P. Ciais, L. A. Cernusak, T. F. Domingues, M. E. Dusenge, S. Garcia, R. Guerrieri, F. Y. Ishida, I. A. Janssens, T. Kenzo, T. Ichie, B. E. Medlyn, P. Meir, R. J. Norby, P. B. Reich, L. Rowland, L. S. Santiago, Y. Sun, J. Uddling, A. P. Walker, K. W. L. K. Weerasinghe, M. J. van de Weg, Y.-B. Zhang, J.-L. Zhang, I. J. Wright, Convergence in phosphorus constraints to photosynthesis in forests around the world. *Nat. Commun.* **13**, 5005 (2022).

54. R. E. Mcmurtrie, H. N. Comins, The temporal response of forest ecosystems to doubled atmospheric CO_2 concentration. *Glob. Chang. Biol.* **2**, 49–57 (1996).

55. S. Manzoni, P. Taylor, A. Richter, A. Porporato, G. I. Ågren, Environmental and stoichiometric controls on microbial carbon-use efficiency in soils. *New Phytol.* **196**, 79–91 (2012).

56. R. E. McMurtrie, R. C. Dewar, B. E. Medlyn, M. P. Jeffreys, Effects of elevated CO_2 on forest growth and carbon storage: A modelling analysis of the consequences of changes in litter quality/quantity and root exudation. *Plant Soil*. **224**, 135–152 (2000).

57. X. He, L. Augusto, D. S. Goll, B. Ringeval, Y.-P. Wang, J. Helfenstein, Y. Huang, E. Hou, Global patterns and drivers of phosphorus pools in natural soils. *Biogeosciences* **20**, 4147–4163 (2023).

58. K. Allen, J. B. Fisher, R. P. Phillips, J. S. Powers, E. R. Brzostek, Modeling the carbon cost of plant nitrogen and phosphorus uptake across temperate and tropical forests. *Front. For. Glob. Change* **3**, 00043 (2020).

59. R. K. Braghiere, J. B. Fisher, K. Allen, E. Brzostek, M. Shi, X. Yang, D. M. Ricciuto, R. A. Fisher, Q. Zhu, R. P. Phillips, Modeling global carbon costs of plant nitrogen and phosphorus acquisition. *J. Adv. Model. Earth Syst.*, **14,** e2022MS003204 (2022).

60. S. Piao, S. Sitch, P. Ciais, P. Friedlingstein, P. Peylin, X. Wang, A. Ahlström, A. Anav, J. G. Canadell, N. Cong, C. Huntingford, M. Jung, S. Levis, P. E. Levy, J. Li, X. Lin, M. R. Lomas, M. Lu, Y. Luo, Y. Ma, R. B. Myneni, B. Poulter, Z. Sun, T. Wang, N. Viovy, S. Zaehle, N. Zeng, Evaluation of terrestrial carbon cycle models for their response to climate variability and to CO_2 trends. *Glob. Chang. Biol.* **19**, 2117–2132 (2013).

61. N. Collier, F. M. Hoffman, D. M. Lawrence, G. Keppel-Aleks, C. D. Koven, W. J. Riley, M. Mu, J. T. Randerson, The International Land Model Benchmarking (ILAMB) System: Design, theory, and implementation. *J. Adv. Model. Earth Syst.* **10**, 2731–2754 (2018).

62. W. R. Wieder, A. S. Grandy, C. M. Kallenbach, P. G. Taylor, G. B. Bonan, Representing life in the Earth system with soil microbial functional traits in the MIMICS model. *Geosci. Model Dev.* **8**, 1789–1808 (2015).

63. G. D. Farquhar, S. von Caemmerer, J. A. Berry, A biochemical model of photosynthetic CO_2 assimilation in leaves of C3 species. *Planta* **149**, 78–90 (1980).

64. G. J. Collatz, J. T. Ball, C. Grivet, J. A. Berry, Physiological and environmental regulation of stomatal conductance, photosynthesis and transpiration: A model that includes a laminar boundary layer. *Agric. For. Meteorol.* **54**, 107–136 (1991).

65. O. Kull, B. Kruijt, Leaf photosynthetic light response: A mechanistic model for scaling photosynthesis to leaves and canopies. *Funct. Ecol.* **12**, 767–777 (1998).

66. S. Fatichi, S. Leuzinger, C. Körner, Moving beyond photosynthesis: From carbon source to sink-driven vegetation modeling. *New Phytol.* **201**, 1086–1095 (2014).

67. A. P. Walker, A. P. Beckerman, L. Gu, J. Kattge, L. A. Cernusak, T. F. Domingues, J. C. Scales, G. Wohlfahrt, S. D. Wullschleger, F. I. Woodward, The relationship of leaf photosynthetic traits–V_{cmax} and J_{max}–to leaf nitrogen, leaf phosphorus, and specific leaf area: A meta-analysis and modeling study. *Ecol. Evol.* **4**, 3218–3235 (2014).

68. M. J. Hedley, J. W. B. Stewart, Method to measure microbial phosphate in soils. *Soil Biol. Biochem.* **14**, 377–385 (1982).

69. R. Wang, D. Goll, Y. Balkanski, D. Hauglustaine, O. Boucher, P. Ciais, I. Janssens, J. Penuelas, B. Guenet, J. Sardans, L. Bopp, N. Vuichard, F. Zhou, B. Li, S. Piao, S. Peng, Y. Huang, S. Tao, Global forest carbon uptake due to nitrogen and phosphorus deposition from 1850 to 2100. *Glob. Chang. Biol.* **23**, 4854–4872 (2017).

70. P. E. Thornton, N. E. Zimmermann, An improved canopy integration scheme for a land surface model with prognostic canopy structure. *J. Clim.* **20**, 3902–3923 (2007).

71. H. Hartmann, S. Trumbore, Understanding the roles of nonstructural carbohydrates in forest trees—From what we can measure to what we want to know. *New Phytol.* **211**, 386–403 (2016).

72. Y. Sun, D. S. Goll, J. Chang, P. Ciais, B. Guenet, J. Helfenstein, Y. Huang, R. Lauerwald, F. Maignan, V. Naipal, Y. Wang, H. Yang, H. Zhang, Global evaluation of the nutrient-enabled version of the land surface model ORCHIDEE-CNP v1.2 (r5986). *Geosci. Model Dev.* **14**, 1987–2010 (2021).

73. V. Haverd, B. Smith, L. P. Nieradzik, P. R. Briggs, A stand-alone tree demography and landscape structure module for Earth system models: Integration with inventory data from temperate and boreal forests. *Biogeosciences* **11**, 4039–4055 (2014).

74. V. Haverd, M. Cuntz, L. P. Nieradzik, I. N. Harman, Improved representations of coupled soil–canopy processes in the CABLE land surface model (Subversion revision 3432). *Geosci. Model Dev.* **9**, 3111–3122 (2016).

75. Smith, I. C. Prentice, M. T. Sykes, Representation of vegetation dynamics in the modelling of terrestrial ecosystems: Comparing two contrasting approaches within European climate space. Glob. Ecol. Biogeogr. 10, 621–637 (2001).

76. D. Ricciuto, K. Sargsyan, P. Thornton, The impact of parametric uncertainties on biogeochemistry in the E3SM land model. *J. Adv. Model. Earth Syst.* **10**, 297–319 (2018).

77. K. Oleson, D. Lawrence, G. Bonan, B. Drewniak, M. Huang, C. Koven, S. Levis, F. Li, W. Riley, Z. Subin, S. Swenson, P. Thornton, A. Bozbiyik, R. Fisher, C. Heald, E. Kluzek, J.-F. Lamarque, P. Lawrence, L. Leung, W. Lipscomb, S. Muszala, D. Ricciuto, W. Sacks, Y. Sun, J. Tang, Z.-L. Yang, *Technical description of version 4.5 of the Community Land Model (CLM)* (UCAR/NCAR, 2013); https://doi.org/10.5065/D6RR1W7M.

78. X. Yang, W. M. Post, Phosphorus transformations as a function of pedogenesis: A synthesis of soil phosphorus data using Hedley fractionation method. *Biogeosciences* **8**, 2907–2916 (2011).

79. A. Haxeltine, I. C. Prentice, BIOME3: An equilibrium terrestrial biosphere model based on ecophysiological constraints, resource availability, and competition among plant functional types. *Global Biogeochem. Cycles* **10**, 693–709 (1996).

80. T. Hickler, B. Smith, M. T. Sykes, M. B. Davis, S. Sugita, K. Walker, Using a generalized vegetation model to simulate vegetation dynamics in northeastern USA. *Ecology* **85**, 519–530 (2004).

81. S. Zaehle, A. D. Friend, Carbon and nitrogen cycle dynamics in the O-CN land surface model: 1. Model description, site-scale evaluation, and sensitivity to parameter estimates. *Global Biogeochem. Cycles* **24**, GB1005 (2010).

82. A. D. Friend, A. K. Stevens, R. G. Knox, M. G. R. Cannell, A process-based, terrestrial biosphere model of ecosystem dynamics (Hybrid v3.0). *Ecol. Model.* **95**, 249–287 (1997).

83. V. S. Pathare, K. Y. Crous, J. Cooke, D. Creek, O. Ghannoum, D. S. Ellsworth, Water availability affects seasonal CO_2-induced photosynthetic enhancement in herbaceous species in a periodically dry woodland. *Glob. Chang. Biol.* **23**, 5164–5178 (2017).

84. S. J. Cork, I. D. Hume, T. J. Dawson, Digestion and metabolism of a natural foliar diet (Eucalyptus punctata) by an arboreal marsupial, the koala (Phascolarctos cinereus). *J. Comp. Physiol. B* **153**, 181–190 (1983).

85. J. E. Drake, C. A. Macdonald, M. G. Tjoelker, P. B. Reich, B. K. Singh, I. C. Anderson, D. S. Ellsworth, Three years of soil respiration in a mature eucalypt woodland exposed to atmospheric CO2 enrichment. *Biogeochemistry* 139, 85–101 (2018).

86. B. E. Medlyn, R. A. Duursma, D. Eamus, D. S. Ellsworth, I. C. Prentice, C. V. M. Barton, K. Y. Crous, P. De Angelis, M. Freeman, L. Wingate, Reconciling the optimal and empirical approaches to modelling stomatal conductance. *Glob. Chang. Biol.* 17, 2134–2144 (2011).

87. Y. P. Wang, R. M. Law, B. Pak, A global model of carbon, nitrogen and phosphorus cycles for the terrestrial biosphere. *Biogeosciences* 7, 2261–2282 (2010).

88. P. E. Thornton, B. E. Law, H. L. Gholz, K. L. Clark, E. Falge, D. S. Ellsworth, A. H. Goldstein, R. K. Monson, D. Hollinger, M. Falk, J. Chen, J. P. Sparks, Modeling and measuring the needleleafforests. *Agric. For. Meteorol.* 113, 185–222 (2002).
